# Supplementary material for: Pathogenic Effects of Mineralocorticoid Pathway Activation in Retinal Pigment Epithelium
Source: Int J Mol Sci. 2021 Sep 5;22(17):9618. doi: 10.3390/ijms22179618 (PMC8431771; doi:10.3390/ijms22179618)
Supplement: Supplementary file 1 [file ijms-22-09618-s001.zip › ijms-1338421-supplementary.pdf]

**Supplementary Table S21.** List of primary and secondary antibodies used in this study.

| Antibody | | Provider | Cat. Nb. | Dilution ICC | Dilution WB | Host |
|---|---|---|---|---|---|---|
| **Primary antibodies** | | | | | | |
| **Albumin** | | Santa Cruz | | 1:100 | - | Goat |
| **BEST1** | Bestrophin 1 Antibody (E6-6) | Novus Biologicals | NB300-164 | 1:250 | - | Mouse |
| **H3F3A** | Anti-Histone H3 Antibody, CT, pan | Sigma-Aldrich | 07-690 | - | 1:5000 | Rabbit |
| **MERTK** | Anti-MERTK antibody [Y323] | Abcam | ab52968 | 1:100 | - | Rabbit |
| **NR3C1** | Glucocorticoid Receptor (D6H2L) XP® Rabbit mAb | Cell Signaling | 12041 | 1:100 | 1:1000 | Rabbit |
| **NR3C2** | Anti-Mineralocorticoid Receptor Antibody, clone 6G1 | MERCK | MABS496 | 1:100 | 1:50 | Mouse |
| **OCLN** | Occludin Polyclonal Antibody | Thermo Fisher SCIENTIFIC | 71-1500 | 1:200 | - | Rabbit |
| **Phalloidin** | Rhodamine Phalloidin | Thermo Fisher SCIENTIFIC | R415 | 1:100 | - | N/A |
| **PTGER2** | Recombinant Anti-Prostaglandin E Receptor EP2/PTGER2 antibody [EPR8030(B)] | Abcam | ab167171 | - | 1:2000 | Rabbit |
| **RLBP1** | Anti-CRALBP antibody [B2] | Abcam | ab15051 | 1:200 | - | Mouse |
| **RPE65** | N/A | Provided by Christian Grimm | (Wenzel *et al*, 2005) | 1:200 | - | Rabbit |
| **S100A2** | S100A2 Polyclonal Antibody | Thermo Fisher SCIENTIFIC | PA5-31861 | 1:100 | 1:1000 | Rabbit |
| **SCNN1A** | alpha-ENaC Polyclonal Antibody | Thermo Fisher SCIENTIFIC | PA1-920A | 1:100 | 1:250 | Rabbit |
| **TJP1** | ZO-1 Polyclonal Antibody | Thermo Fisher SCIENTIFIC | 40-2200 | 1:250 | - | Rabbit |
| **TUBA1A** | Anti-alpha Tubulin antibody [DM1A] | Abcam | ab7291 | - | 1:2000 | Mouse |
| **Secondary antibodies** | | | | | | |
| **GAR** | F(ab')2-Goat anti-Rabbit IgG (H+L) Cross-Adsorbed Secondary Antibody, Alexa Fluor 488 | Thermo Fisher SCIENTIFIC | A-11070 | 1:1000 | - | Goat |
| **GAM** | F(ab')2-Goat anti-Mouse IgG (H+L) Cross-Adsorbed Secondary Antibody, Alexa Fluor 633 | Thermo Fisher SCIENTIFIC | A-21053 | 1:1000 | - | Goat |
| **DAG** | Alexa Fluor 596-coupled donkey anti-goat IgG | | | 1:200 | - | Donkey |
| **AR** | Rabbit IgG HRP Linked Whole Ab | Sigma-Aldrich | GENA934 | - | 1:5000 | Rabbit |
| **AM** | Mouse IgG HRP Linked Whole Ab | Sigma-Aldrich | GENA931 | - | 1:5000 | Mouse |

ICC, immunocytochemistry; WB, western blot; GAR, goat anti-rabbit; GAM, goat anti-mouse; DAG, donkey anti-goat; AR, anti-rabbit; AM, anti-mouse.

**Supplementary Table S20**. List of PCR and qPCR primers used in this study.

| Gene | Primers sequence | | Amplicon length | Design |
|---|---|---|---|---|
| **Human qPCR Primers** | | | | |
| ***ABCC3*** | Forward<br>Reverse | 5'-GAGAAGGGCATTAACCTGTC-3'<br>5'-CGTGGTCAAAGATGTGCTTG-3' | **142** | NM_003786.4 / Exon 17 - Exon 18 junction<br>NM_003786.4 / Exon 18 |
| ***BEST1*** | Forward<br>Reverse | 5'-CCTTGGAAAACAGGGATGAAGC-3'<br>5'-AGCTGTATGGCTGTGACTGG-3' | **120** | NM_004183.3 / Exon 10 - Exon 11 junction<br>NM_004183.3 / Exon 11 |
| ***FKBP5*** | Forward<br>Reverse | 5'-CGGCGACAGGTTCTCTACTT-3'<br>5'-ATCGGCGTTTCCTCACCATT-3' | **171** | NM_004117.3 / Exon 1 - Exon 2 junction<br>NM_004117.3 / Exon 3 |
| ***GAPDH*** | Forward<br>Reverse | 5'-CTCTGCTCCTCCTGTTCGAC-3'<br>5'-ACCAAATCCGTTGACTCCGA-3' | **109** | NM_002046.5 / Exon 1<br>NM_002046.5 / Exon 1 - Exon 2 junction |
| ***HMGA1*** | Forward<br>Reverse | 5'-CTCCAAGAAGGCCATCCCAG-3'<br>5'-TCTCAGTGCCGTCCTTTTCC-3' | **123** | NM_145903.2 / Exon 1 - Exon 2 junction<br>NM_145903.2 / Exon 2 |
| ***HSD11B1*** | Forward<br>Reverse | 5'-CTGCCTGCTTAGGAGGTTGT-3'<br>5'-TCCTTGGAGCATCTCTGGT-3' | **184** | NM_005525.4 / Exon 1<br>NM_005525.4 / Exon 1 - Exon 2 junction |
| ***HSD11B2*** | Forward<br>Reverse | 5'-CTCATCACCGGCTGTGACTC-3'<br>5'-CTCCTGGTTTGGTCAGGTCC-3' | **175** | NM_000196.4 / Exon 1 - Exon 2 junction<br>NM_000196.4 / Exon 2 |
| ***LCN2*** | Forward<br>Reverse | 5'-ACAATGTCACCTCCGTCCTG-3'<br>5'-AGCTCCTTGGTTCTCCCGTA-3' | **241** | NM_005564.4 / Exon 2<br>NM_005564.4 / Exon 4 - Exon 5 junction |
| ***LRAT*** | Forward<br>Reverse | 5'-TACTGCAGATATGGCACCCC-3'<br>5'-CCAAGACTGCTGAAGCAAGA-3' | **106** | NM_004744.4 / Exon 2<br>NM_004744.4 / Exon 3 |
| ***MERTK*** | Forward<br>Reverse | 5'-AAGGCCGCATTGCTAAGAT-3'<br>5'-CGCGTAGCTATTTCCCACAT-3' | **121** | NM_006343.2 / Exon 17<br>NM_006343.2 / Exon 18 |
| ***NR3C1***<br>GR | Forward<br>Reverse | 5’-AAGAGCAGTGGAAGGACAGC-3’<br>5’-CCAGGTTCATTCCAGCCTGA-3’ | **128** | NM_000176.3 / Exon 3 - Exon 4 junction<br>NM_000176.3 / Exon 4 |
| ***NR3C2***<br>MR | Forward<br>Reverse | 5’-AGTGGAAGGGCAACACAACT-3’<br>5’-ACTTCTTTGACTTTCGTGCTCC-3’ | **143** | NM_000901.5 / Exon 3 - Exon 4 junction<br>NM_000901.5 / Exon 4 - Exon 5 junction |
| ***PER1*** | Forward<br>Reverse | 5'-TCATGATGACCTACCAGGTG-3'<br>5'-ACACAGGCCATCACATCAAG-3' | **187** | NM_002616.2 / Exon<br>NM_002616.2 / Exon - Exon junction |
| ***PLAUR*** | Forward<br>Reverse | 5'-TGTGGGAAGAAGGAGAAGAG-3'<br>5'-CCTGGTTGCACAAGTCTAAC-3' | **144** | NM_002659.3 / Exon 2 - Exon 3 junction<br>NM_002659.3 / Exon 3 |
| ***PMEL*** | Forward<br>Reverse | 5'-GCCTGGCAGTGGTCAGCACC-3'<br>5'-CGGGGTAGACGCAGCCAGTGA-3' | **199** | NM_006928.4 / Exon 7<br>NM_006928.4 / Exon 7 |
| ***PTGER2*** | Forward<br>Reverse | 5'-ACAGATGTGCTGACAAGGCACT-3'<br>5'-TGCAGCCGATTGTTCCTCCAAA-3' | **103** | NM_000956.3 / Exon 2<br>NM_000956.3 / Exon 2 |
| ***PTX3*** | Forward<br>Reverse | 5'-CCGGCAGGTTGTGAAACAG-3'<br>5'-CTCCACCCACCACAAACACT-3' | **229** | NM_002852.3 / Exon 2 - Exon 3 junction<br>NM_002852.3 / Exon 3 |
| ***RDH5*** | Forward<br>Reverse | 5'-GGAGGCCTTCTCTGACAGC-3'<br>5'-CCAGACTCTCCAGGTTGGTC-3' | **110** | NM_002905.3 / Exon 3<br>NM_002905.3 / Exon 4 |
| ***RLBP1*** | Forward<br>Reverse | 5'-AGATCTCAGGAAGATGGTGGAC-3'<br>5'-GAAGTGGATGGCTTTGAACC-3' | **67** | NM_000326.4 / Exon 7<br>NM_000326.4 / Exon 8 |
| ***RPE65*** | Forward<br>Reverse | 5'-CAATGGGTTTCTGATTGTGGA-3'<br>5'-CCAGTTCTCACGTAAATTGGCTA-3' | **91** | NM_000329.2 / Exon 9<br>NM_000329.2 / Exon 10 |
| ***S100A2*** | Forward<br>Reverse | 5'-AAGAGGGCGACAAGTTCAAG-3'<br>5'-ACTGTTCTCATCCAGGCTGC-3' | **134** | NM_005978.3 / Exon 2<br>NM_005978.3 / Exon 3 |
| ***SCNN1A***<br>αENaC | Forward<br>Reverse | 5'-CTCTGTCACGATGGTCACCC-3'<br>5'-GACCAGCAGGTCAAAGACGA-3' | **115** | NM_001159576.1 / Exon 11 - Exon 12 junction<br>NM_001159576.1 / Exon 12 |
| ***SERPINA3*** | Forward<br>Reverse | 5'-AAGGACCTTGACTCGCAGAC-3'<br>5'-ATGGGCACCATTACCCACT-3' | **140** | NM_001085.4 / Exon 2<br>NM_001085.4 / Exon 3 |
| ***SGK1*** | Forward<br>Reverse | 5'-CGGAGAAGGACCAGCTGAAG-3'<br>5'-CCCAACAAGCACGTTGGAAG-3' | **108** | NM_5627.3 / Exon 8 - Exon 9 junction<br>NM_5627.3 / Exon 9 - Exon 10 junction |

| | | | | |
|---|---|---|---|---|
| ***TSC22D3*** GILZ | Forward<br>Reverse | 5'-TGGTGGCCATAGACAACAAG-3'<br>5'-CACGCTCTAGCTGGGAGTTC-3' | **138** | NM_004089.3 / Exon 2<br>NM_004089.3 / Exon 3 |
| ***TYR*** | Forward<br>Reverse | 5'-ACTTACTCAGCCCAGCATC-3'<br>5'-GGTTTCCAGGATTACGCC-3' | **129** | NM_000372.4 / Exon 1<br>NM_000372.4 / Exon 2 |
| ***TYRP1*** | Forward<br>Reverse | 5'-CCGAAACACAGTGGAAGGTT-3'<br>5'-TCTGTGAAGGTGTGCAGGA-3' | **159** | NM_000550.2 / Exon 5 - Exon 6 junction<br>NM_000550.2 / Exon 6 |

Rat qPCR Primers

| | | | | |
|---|---|---|---|---|
| ***Plaur*** | Forward<br>Reverse | 5'-CTGAAGTGCTGCAACTTCAC-3'<br>5'-AGCACATCTAAGCCTGTAGC-3' | **188** | NM_017350.1 / Exon 5<br>NM_017350.1 / Exon 8 - Exon 9 junction |
| ***Ptger2*** | Forward<br>Reverse | 5'-TGCGGATTGTCTGGCAGTAG-3'<br>5'-AGCAAAGATTGTGAAAGGCAGG-3' | **150** | NM_031088.1<br>NM_031088.1 / Exon 1 - Exon 2 junction |
| ***Ptx3*** | Forward<br>Reverse | 5'-TACCCGCAGGCTGTGAAAC-3'<br>5'-GGGTTCCACTTGGTGCCATA-3' | **175** | NM_001109536.1 / Exon 2 - Exon 3 junction<br>NM_001109536.1 / Exon 3 |
| ***Rpl8*** | Forward<br>Reverse | 5'-TGCTAACCGAGCTGTTGTTG-3'<br>5'-CTCCACAGGATTCATGGCAA-3' | **145** | NM_001034916.2 / Exon 3<br>NM_001034916.2 / Exon 4 - Exon 5 junction |
| ***Scnn1a*** αENaC | Forward<br>Reverse | 5'-TGCTAACCGAGCTGTTGTTG-3'<br>5'-CTCCACAGGATTCATGGCAA-3' | **145** | NM_001034916.2 / Exon 3<br>NM_001034916.2 / Exon 4 - Exon 5 junction |

Mouse qPCR Primers

| | | | | |
|---|---|---|---|---|
| ***β-Actin*** | Forward<br>Reverse | 5'-AAGTACCCCATTGAACATGGCA-3'<br>5'-CATCTTTTCACGGTTGGCCTTA-3' | **156** | NM_007393.5 / Exon 3<br>NM_007393.5 / Exon 3 |
| ***Nr3c1*** GR | Forward<br>Reverse | **Canonica, Jeremie** Could you please provide me with the primers sequence you used to quantify mouse *Nr3c1* mRNA transcripts relative expression? Many thanks in advance. | | |
| ***Nr3c2*** MR | Forward<br>Reverse | 5'-ATGGAAACCACACGGTGACCT-3'<br>5'-GCCTCATCTCCACACACCAAG-3' | **153** | NM_001083906.2 / Exon 2<br>NM_001083906.2 / Exon 3 |
| ***Human Nr3c2*** hMR | Forward<br>Reverse | 5'-CCCTCTGAACATGACATCTTCG-3'<br>5'-CTGGAGCCTCGATTTTCAAC-3' | **153** | NM_000901.5 / Exon 2<br>NM_000901.5 / Exon 2 |

**Supplementary Table S8.** Specific aldosterone-regulated enriched REACTOME pathways as predicted by bioinformatic enrichment analysis.

**REACTOME pathway** analysis on DEGs

**Aldosterone $10^{-7}$ M *versus* Control**

| REACTOME Pathway Description | Link to REACTOME | Gene name | Description | Regulation | FC | *P*-value |
|---|---|---|---|---|---|---|
| **ECM proteoglycans** | R-HSA-3000178 | **ITGB3** | EF-hand calcium binding domain 13 // integrin subunit beta 3 | up | **1.71** | 3.11E-02 |
| | | **ITGAX** | Integrin subunit alpha X | up | **1.51** | 1.27E-02 |
| | | **FMOD** | Fibromodulin | down | **-2.09** | 3.28E-02 |
| | | **BGN** | Biglycan | down | **-1.57** | 3.68E-03 |
| | | **COL2A1** | Collagen type II alpha 1 chain | down | **-1.52** | 2.59E-03 |
| **Integrin cell surface interactions** | R-HSA-216083 | **ITGB3** | EF-hand calcium binding domain 13 // integrin subunit beta 3 | up | **1.71** | 3.11E-02 |
| | | **ITGA4** | integrin subunit alpha 4 | up | **1.53** | 2.06E-03 |
| | | **ITGAX** | Integrin subunit alpha X | up | **1.51** | 1.27E-02 |
| | | **COL2A1** | Collagen type II alpha 1 chain | down | **-1.52** | 2.59E-03 |
| **Dissolution of Fibrin Clot** | R-HSA-75205 | **PLAUR** | Plasminogen activator, urokinase receptor | up | **2.16** | 6.77E-03 |
| | | **PLAT** | Plasminogen activator, tissue type | up | **1.60** | 1.38E-02 |

Up, up-regulated genes in the REACTOME pathway; down, down-regulated genes in the REACTOME pathway; DEG, differentially expressed gene; FC, fold-change.

**Supplementary Table S9.** Specific cortisol-regulated enriched REACTOME pathways as predicted by bioinformatic enrichment analysis.

**REACTOME pathway** analysis on DEGs

**Cortisol $10^{-7}$ M *versus* Control**

| REACTOME Pathway Description | Link to REACTOME | Gene name | Description | Regulation | FC | *P*-value |
|---|---|---|---|---|---|---|
| **Regulation of Insulin-like Growth Factor transport and uptake by Insulin-like Growth Factor Binding Proteins** | R-HSA-381426 | **IGF1** | Insulin like growth factor 1 | down | **-1.85** | 1.51E-18 |
| | | **PAPPA** | Pappalysin 1 | down | **-1.66** | 1.58E-02 |
| | | **IGFBP3** | Insulin like growth factor binding protein 3 | down | **-1.64** | 1.77E-03 |
| **Integrin cell surface interactions** | R-HSA-216083 | **SPP1** | Secreted phosphoprotein 1 | down | **-2.25** | 5.38E-05 |
| | | **ITGA11** | Integrin subunit alpha 11 | down | **-2.24** | 3.58E-07 |
| | | **COL1A1** | Collagen type I alpha 1 chain | down | **-1.68** | 3.34E-03 |
| **O-glycosylation of TSR domain-containing proteins** | R-HSA-5173214 | **ADAMTSL1** | ADAMTS like 1 | up | **1.53** | 3.58E-05 |
| | | **ADAMTSL4** | ADAMTS like 4 | up | **1.53** | 4.94E-18 |
| | | **ADAMTS1** | ADAM metallopeptidase with thrombospondin type 1 motif 1 | down | **-1.57** | 8.90E-12 |

Up, up-regulated genes in the REACTOME pathway; down, down-regulated genes in the REACTOME pathway; DEG, differentially expressed gene; FC, fold-change.

**Supplementary Table S10.** Specific cortisol + RU-486-regulated enriched REACTOME pathways as predicted by bioinformatic enrichment analysis.

**REACTOME pathway** analysis on DEGs

**Cortisol $10^{-7}$ M + RU-486 $10^{-5}$ M *versus* Control**

| REACTOME Pathway Description | Link to REACTOME | Gene name | Description | Regulation | FC | *P*-value |
|---|---|---|---|---|---|---|
| **Cyclin A/B1/B2 associated events during G2/M transition** | R-HSA-69273 | **CCNA1** | Cyclin A1 | down | **-1.92** | 1.29E-07 |
| | | **PLK1** | Polo like kinase 1 | down | **-1.72** | 2.43E-04 |
| | | **CCNA2** | Cyclin A2 | down | **-1.72** | 1.60E-07 |
| | | **FOXM1** | Forkhead box M1 | down | **-1.62** | 3.17E-03 |
| | | **CDK1** | Cyclin dependent kinase 1 | down | **-1.62** | 4.47E-10 |
| **G0 and Early G1** | R-HSA-1538133 | **CCNA1** | Cyclin A1 | down | **-1.92** | 1.29E-07 |
| | | **CCNA2** | Cyclin A2 | down | **-1.72** | 1.60E-07 |
| | | **CDK1** | Cyclin dependent kinase 1 | down | **-1.62** | 4.47E-10 |
| | | **TOP2A** | Topoisomerase (DNA) II alpha | down | **-1.56** | 2.82E-06 |
| | | **MYBL2** | MYB proto-oncogene like 2 | down | **-1.56** | 8.14E-06 |
| **Resolution of Sister Chromatid Cohesion** | R-HSA-2500257 | **SPC24** | SPC24, NDC80 kinetochore complex component | down | **-1.84** | 4.74E-07 |
| | | **BUB1B** | BUB1 mitotic checkpoint serine/threonine kinase B | down | **-1.81** | 2.76E-11 |
| | | **PLK1** | Polo like kinase 1 | down | **-1.72** | 2.43E-04 |
| | | **CENPF** | Centromere protein F | down | **-1.63** | 2.37E-05 |
| | | **CDK1** | Cyclin dependent kinase 1 | down | **-1.62** | 4.47E-10 |
| | | **CENPM** | Centromere protein M | down | **-1.58** | 5.36E-03 |
| | | **SPC25** | SPC25, NDC80 kinetochore complex component | down | **-1.54** | 3.80E-02 |
| | | **CDC20** | Cell division cycle 20 | down | **-1.52** | 1.12E-03 |
| **RHO GTPases Activate Formins** | R-HSA-5663220 | **SPC24** | SPC24, NDC80 kinetochore complex component | down | **-1.84** | 4.74E-07 |

| | | | | | | |
|---|---|---|---|---|---|---|
| | | **BUB1B** | BUB1 mitotic checkpoint serine/threonine kinase B | down | **-1.81** | 2.76E-11 |
| | | **PLK1** | Polo like kinase 1 | down | **-1.72** | 2.43E-04 |
| | | **CENPF** | Centromere protein F | down | **-1.63** | 2.37E-05 |
| | | **CENPM** | Centromere protein M | down | **-1.58** | 5.36E-03 |
| | | **SPC25** | SPC25, NDC80 kinetochore complex component | down | **-1.54** | 3.80E-02 |
| | | **DIAPH3** | Diaphanous related formin 3 | down | **-1.53** | 1.22E-08 |
| | | **CDC20** | Cell division cycle 20 | down | **-1.52** | 1.12E-03 |
| | | | | | | |
| **Phosphorylation of proteins involved in the G2/M transition by Cyclin A:Cdc2 complexes** | R-HSA-170145 | **CCNA1** | Cyclin A1 | down | **-1.92** | 1.29E-07 |
| | | **CCNA2** | Cyclin A2 | down | **-1.72** | 1.60E-07 |
| | | **CDK1** | Cyclin dependent kinase 1 | down | **-1.62** | 4.47E-10 |
| | | | | | | |
| **Polo-like kinase mediated events** | R-HSA-156711 | **PLK1** | Polo like kinase 1 | down | **-1.72** | 2.43E-04 |
| | | **CENPF** | Centromere protein F | down | **-1.63** | 2.37E-05 |
| | | **FOXM1** | Forkhead box M1 | down | **-1.62** | 3.17E-03 |
| | | **MYBL2** | MYB proto-oncogene like 2 | down | **-1.56** | 8.14E-06 |
| | | | | | | |
| **Mitotic Prometaphase** | R-HSA-68877 | **SPC24** | SPC24, NDC80 kinetochore complex component | down | **-1.84** | 4.74E-07 |
| | | **BUB1B** | BUB1 mitotic checkpoint serine/threonine kinase B | down | **-1.81** | 2.76E-11 |
| | | **PLK1** | Polo like kinase 1 | down | **-1.72** | 2.43E-04 |
| | | **CENPF** | Centromere protein F | down | **-1.63** | 2.37E-05 |
| | | **CENPM** | Centromere protein M | down | **-1.58** | 5.36E-03 |
| | | **SPC25** | SPC25, NDC80 kinetochore complex component | down | **-1.54** | 3.80E-02 |
| | | **CDC20** | Cell division cycle 20 | down | **-1.52** | 1.12E-03 |
| | | | | | | |
| **Phosphorylation of Emi1** | R-HSA-176417 | **PLK1** | Polo like kinase 1 | down | **-1.72** | 2.43E-04 |
| | | **CDK1** | Cyclin dependent kinase 1 | down | **-1.62** | 4.47E-10 |
| | | **CDC20** | Cell division cycle 20 | down | **-1.52** | 1.12E-03 |

| | | | | | | |
|---|---|---|---|---|---|---|
| **Regulation of APC/C activators between G1/S and early anaphase** | R-HSA-176408 | **CCNA1** | Cyclin A1 | down | **-1.92** | 1.29E-07 |
| | | **CCNA2** | Cyclin A2 | down | **-1.72** | 1.60E-07 |
| | | **CDK1** | Cyclin dependent kinase 1 | down | **-1.62** | 4.47E-10 |
| | | **CDC20** | Cell division cycle 20 | down | **-1.52** | 1.12E-03 |
| **Cdc20:Phospho-APC/C mediated degradation of Cyclin A** | R-HSA-174184 | **CCNA1** | Cyclin A1 | down | **-1.92** | 1.29E-07 |
| | | **BUB1B** | BUB1 mitotic checkpoint serine/threonine kinase B // p21 (RAC1) activated kinase 6 | down | **-1.81** | 2.76E-11 |
| | | **CCNA2** | Cyclin A2 | down | **-1.72** | 1.60E-07 |
| | | **CDK1** | Cyclin dependent kinase 1 | down | **-1.62** | 4.47E-10 |
| | | **CDC20** | Cell division cycle 20 | down | **-1.52** | 1.12E-03 |
| **Dissolution of Fibrin Clot** | R-HSA-75205 | **PLAUR** | Plasminogen activator, urokinase receptor | up | **2.38** | 1.14E-10 |
| | | **SERPINE2** | Serpin family E member 2 | up | **2.12** | 3.52E-09 |
| | | **SERPINE1** | Serpin family E member 1 | up | **1.54** | 2.46E-03 |
| **Separation of Sister Chromatids** | R-HSA-2467813 | **SPC24** | SPC24, NDC80 kinetochore complex component | down | **-1.84** | 4.74E-07 |
| | | **BUB1B** | BUB1 mitotic checkpoint serine/threonine kinase B | down | **-1.81** | 2.76E-11 |
| | | **PLK1** | Polo like kinase 1 | down | **-1.72** | 2.43E-04 |
| | | **CENPF** | Centromere protein F | down | **-1.63** | 2.37E-05 |
| | | **CENPM** | Centromere protein M | down | **-1.58** | 5.36E-03 |
| | | **SPC25** | SPC25, NDC80 kinetochore complex component | down | **-1.54** | 3.80E-02 |
| | | **CDC20** | Cell division cycle 20 | down | **-1.52** | 1.12E-03 |
| **Degradation of the extracellular matrix** | R-HSA-1474228 | **MMP1** | Matrix metallopeptidase 1 | up | **6.06** | 2.25E-08 |
| | | **LAMC2** | Laminin subunit gamma 2 | up | **1.76** | 5.04E-12 |
| | | **CD44** | CD44 molecule (Indian blood group) | up | **1.54** | 2.73E-09 |

| | | | | | | |
|---|---|---|---|---|---|---|
| | | **ADAMTS5** | ADAM metallopeptidase with thrombospondin type 1 motif 5 | down | **-2.24** | 3.58E-08 |
| | | **MMP11** | Matrix metallopeptidase 11 | down | **-1.76** | 9.27E-23 |
| | | **CAPN6** | Calpain 6 | down | **-1.67** | 3.35E-12 |
| **PPARA activates gene expression** | R-HSA-1989781 | **ANGPTL4** | Angiopoietin like 4 | up | **3.56** | 2.07E-31 |
| | | **HMGCS2** | 3-hydroxy-3-methylglutaryl-CoA synthase 2 | up | **2.96** | 5.35E-54 |
| | | **PLIN2** | Perilipin 2 | up | **2.83** | 3.52E-64 |
| | | **TRIB3** | Tribbles pseudokinase 3 | up | **1.71** | 2.94E-06 |
| | | **CPT1A** | Carnitine palmitoyltransferase 1A | up | **1.67** | 3.98E-52 |
| **Reversible hydration of carbon dioxide** | R-HSA-1475029 | **CA12** | Carbonic anhydrase 12 | up | **2.28** | 1.56E-06 |
| | | **CA2** | Carbonic anhydrase 2 | down | **-1.55** | 2.48E-06 |
| | | **CA9** | Carbonic anhydrase 9 | down | **-1.50** | 7.90E-24 |
| **Integrin cell surface interactions** | R-HSA-216083 | **EFCAB13** | EF-hand calcium binding domain 13 | up | **1.94** | 2.60E-06 |
| | | **CD44** | CD44 molecule (Indian blood group) | up | **1.54** | 2.73E-09 |
| | | **ITGA11** | Integrin subunit alpha 11 | down | **-2.19** | 7.41E-07 |
| | | **LUM** | Lumican | down | **-1.72** | 6.80E-05 |
| | | **COL1A1** | Collagen type I alpha 1 chain | down | **-1.64** | 4.98E-03 |
| | | **COL3A1** | Collagen type III alpha 1 chain | down | **-1.52** | 1.44E-13 |
| **Basigin interactions** | R-HSA-210991 | **MMP1** | Matrix metallopeptidase 1 | up | **6.06** | 2.25E-08 |
| | | **ATP1B2** | ATPase $Na^{+}/K^{+}$ transporting subunit beta 2 | up | **1.57** | 1.62E-10 |
| | | **SLC7A11** | Solute carrier family 7 member 11 | up | **1.52** | 6.66E-14 |
| **APC-Cdc20 mediated degradation of Nek2A** | R-HSA-179409 | **BUB1B** | BUB1 mitotic checkpoint serine/threonine kinase B | down | **-1.81** | 2.76E-11 |
| | | **NEK2** | NIMA related kinase 2 | down | **-1.77** | 2.50E-03 |
| | | **CDC20** | Cell division cycle 20 | down | **-1.52** | 1.12E-03 |

| | | | | | | |
|---|---|---|---|---|---|---|
| **Platelet degranulation** | R-HSA-114608 | **RAB27B** | RAB27B, member RAS oncogene family | up | **2.05** | 2.46E-14 |
| | | **EFCAB13** | EF-hand calcium binding domain 13 | up | **1.94** | 2.60E-06 |
| | | **ECM1** | Extracellular matrix protein 1 | up | **1.82** | 3.16E-06 |
| | | **SERPINA3** | Serpin family A member 3 | up | **1.62** | 3.90E-07 |
| | | **SERPINE1** | Serpin family E member 1 | up | **1.54** | 2.46E-03 |
| **Antagonism of Activin by Follistatin** | R-HSA-2473224 | **INHBA** | Inhibin beta A subunit | down | **-1.96** | 6.23E-03 |
| | | **INHBB** | Inhibin beta B subunit | down | **-1.68** | 4.33E-13 |
| **Mitotic Telophase/Cytokinesis** | R-HSA-68884 | **KIF20A** | Kinesin family member 20A | down | **-2.04** | 5.94E-08 |
| | | **PLK1** | Polo like kinase 1 | down | **-1.72** | 2.43E-04 |
| **G2 Phase** | R-HSA-68911 | **CCNA1** | Cyclin A1 | down | **-1.92** | 1.29E-07 |
| | | **CCNA2** | Cyclin A2 | down | **-1.72** | 1.60E-07 |
| **ECM proteoglycans** | R-HSA-3000178 | **EFCAB13** | EF-hand calcium binding domain 13 | up | **1.94** | 2.60E-06 |
| | | **SERPINE1** | Serpin family E member 1 | up | **1.54** | 2.46E-03 |
| | | **LUM** | Lumican | down | **-1.72** | 6.80E-05 |
| | | **COL1A1** | Collagen type I alpha 1 chain | down | **-1.64** | 4.98E-03 |
| | | **COL3A1** | Collagen type III alpha 1 chain | down | **-1.52** | 1.44E-13 |
| **O-glycosylation of TSR domain-containing proteins** | R-HSA-5173214 | **ADAMTS5** | ADAM metallopeptidase with thrombospondin type 1 motif 5 | down | **-2.24** | 3.58E-08 |
| | | **ADAMTS15** | ADAM metallopeptidase with thrombospondin type 1 motif 15 | down | **-1.67** | 1.10E-04 |
| | | **THBS2** | Thrombospondin 2 | down | **-1.53** | 2.41E-06 |

Up, up-regulated genes in the REACTOME pathway; down, down-regulated genes in the REACTOME pathway; DEG, differentially expressed gene; FC, fold-change.

**Supplementary Table S11.** Specific aldosterone-regulated enriched biological process gene ontology terms as predicted by bioinformatic enrichment analysis.

**GO Biological Process** analysis on DEGs

**Aldosterone $10^{-7}$ M *versus* Control**

| GO cellular components term | Link to GO ID | Gene name | Description | Regulation | FC | *P*-value |
|---|---|---|---|---|---|---|
| **Chemical synaptic transmission** | GO:0007268 | **OPRD1** | Opioid receptor delta 1 | up | **1.55** | 2,95E-03 |
| | | **DOC2A** | Double C2 domain alpha | down | **-2.31** | 3,39E-03 |
| | | **PLP1** | Proteolipid protein 1 | down | **-1.87** | 3,19E-02 |
| | | **SST** | Somatostatin | down | **-1.81** | 1,02E-04 |
| | | **GJD2** | Gap junction protein delta 2 | down | **-1.67** | 1,70E-02 |
| | | **TPGS1** | Tubulin polyglutamylase complex subunit 1 | down | **-1.59** | 1,70E-03 |
| | | **KCNMB2** | Potassium calcium-activated channel subfamily M regulatory beta subunit 2 | down | **-1.56** | 1,09E-02 |
| | | **SLC12A5** | Solute carrier family 12 member 5 | down | **-1.52** | 2,40E-03 |
| | | **HTR1D** | 5-hydroxytryptamine receptor 1D | down | **-1.51** | 4,09E-02 |
| **Axonogenesis** | GO:0007409 | **OMD** | Osteomodulin | up | **1.87** | 2,87E-03 |
| | | **DSCAML1** | DS cell adhesion molecule like 1 | up | **1.57** | 7,08E-03 |
| | | **S100A6** | S100 calcium binding protein A6 | up | **1.55** | 2,27E-03 |
| | | **FMOD** | Fibromodulin | down | **-2.09** | 3,28E-02 |
| | | **CTNNA2** | Catenin alpha 2 | down | **-1.79** | 1,23E-02 |
| | | **OMG** | Oligodendrocyte myelin glycoprotein | down | **-1.58** | 3,84E-03 |
| **Cell adhesion** | GO:0007155 | **MYBPC2** | Myosin binding protein C, fast type | up | **1.95** | 4,04E-02 |
| | | **OMD** | Osteomodulin | up | **1.87** | 2,87E-03 |
| | | **SVEP1** | Sushi, von Willebrand factor type A, EGF and pentraxin domain containing 1 | up | **1.73** | 1,08E-02 |
| | | **ITGB3** | Integrin subunit beta 3 | up | **1.71** | 3,11E-02 |
| | | **EPHA8** | EPH receptor A8 | up | **1,70** | 2,77E-02 |
| | | **DSCAML1** | DS cell adhesion molecule like 1 | up | **1.57** | 7,08E-03 |
| | | **ITGA4** | Integrin subunit alpha 4 | up | **1.53** | 2,06E-03 |

| | | | | | | |
|---|---|---|---|---|---|---|
| | | **ITGAX** | Integrin subunit alpha X | up | **1.51** | 1,27E-02 |
| | | **CTNNA2** | Catenin alpha 2 | down | **-1.79** | 1,23E-02 |
| | | **OMG** | Oligodendrocyte myelin glycoprotein | down | **-1.58** | 3,84E-03 |
| **Heterotypic cell-cell adhesion** | GO:0034113 | **ITGB3** | Integrin subunit beta 3 | up | **1.71** | 3,11E-02 |
| | | **ITGA4** | Integrin subunit alpha 4 | up | **1.53** | 2,06E-03 |
| | | **ITGAX** | Integrin subunit alpha X | up | **1.51** | 1,27E-02 |
| **Epithelial cell differentiation** | GO:0030855 | **EHF** | ETS homologous factor | up | **3.55** | 2,68E-04 |
| | | **GDF7** | Growth differentiation factor 7 | down | **-2.01** | 2,21E-03 |
| | | **SIX1** | SIX homeobox 1 | down | **-1.95** | 4,58E-03 |
| | | **KRT4** | Keratin 4 | down | **-1.55** | 4,15E-03 |
| **Response to progesterone** | GO:0032570 | **FOSL1** | FOS like 1, AP-1 transcription factor subunit | up | **1.89** | 3,69E-03 |
| | | **PTGER2** | Prostaglandin E receptor 2 | up | **1.57** | 7,18E-06 |
| | | **RAMP2** | Receptor activity modifying protein 2 | down | **-1.57** | 1,23E-02 |
| **Negative regulation of JAK-STAT cascade** | GO:0046426 | **RTN4RL2** | Reticulon 4 receptor-like 2 | down | **-1.67** | 3,22E-04 |
| | | **BGN** | Biglycan | down | **-1.57** | 3,68E-03 |
| | | **SOCS1** | Suppressor of cytokine signaling 1 | down | **-1.51** | 3,26E-02 |
| **Integrin-mediated signaling pathway** | GO:0007229 | **ITGB3** | Integrin subunit beta 3 | up | **1.71** | 3,11E-02 |
| | | **ITGA4** | Integrin subunit alpha 4 | up | **1.53** | 2,06E-03 |
| | | **ITGAX** | Integrin subunit alpha X | up | **1.51** | 1,27E-02 |
| | | **PLP1** | Proteolipid protein 1 | down | **-1.87** | 3,19E-02 |
| **Negative regulation of protein kinase activity** | GO:0006469 | **RTN4RL2** | Reticulon 4 receptor-like 2 | down | **-1.67** | 3,22E-04 |
| | | **PTPRC** | Protein tyrosine phosphatase, receptor type C | down | **-1.64** | 2,25E-02 |
| | | **BGN** | Biglycan | down | **-1.57** | 3,68E-03 |

| | | | | | | |
|---|---|---|---|---|---|---|
| | | **SOCS1** | Suppressor of cytokine signaling 1 | down | **-1.51** | 3,26E-02 |
| | | | | | | |
| **Otic vesicle development** | GO:0071599 | **SIX1** | SIX homeobox 1 | down | **-1.95** | 4,58E-03 |
| | | **COL2A1** | Collagen type II alpha 1 chain | down | **-1.52** | 2,59E-03 |
| | | | | | | |
| **Response to cAMP** | GO:0051591 | **AGXT** | Alanine-glyoxylate aminotransferase | up | **2.13** | 4,53E-02 |
| | | **FOSL1** | FOS like 1, AP-1 transcription factor subunit | up | **1.89** | 3,69E-03 |
| | | **PER1** | MicroRNA 6883 // period circadian clock 1 | up | **1.51** | 3,53E-06 |

GO, gene ontology; up, up-regulated genes in the biological process gene ontology term; down, down-regulated genes in the biological process gene ontology term; DEG, differentially expressed gene; FC, fold-change.

**Supplementary Table S12.** Specific cortisol-regulated enriched biological process gene ontology terms as predicted by bioinformatic enrichment analysis.

**GO Biological Process** analysis on DEGs

**Cortisol $10^{-7}$ M *versus* Control**

| GO biological process term | Link to GO ID | Gene name | Description | Regulation | FC | *P*-value |
|---|---|---|---|---|---|---|
| **Negative regulation of skeletal muscle tissue development** | GO:0048642 | **TSC22D3** | TSC22 domain family member 3 | up | **1.83** | 2.58E-107 |
| | | **DKK1** | Dickkopf WNT signaling pathway inhibitor 1 | up | **1.76** | 5.76E-04 |
| | | **USP2** | Ubiquitin specific peptidase 2 | up | **1.57** | 4.64E-09 |
| **Positive regulation of apoptotic process** | GO:0043065 | **DUSP6** | Dual specificity phosphatase 6 | up | **1.82** | 2.93E-03 |
| | | **RPS6KA2** | Ribosomal protein S6 kinase A2 | up | **1.79** | 4.35E-45 |
| | | **WNT11** | Wnt family member 11 | up | **1.61** | 3.74E-04 |
| | | **ADAMTSL4** | ADAMTS like 4 | up | **1.53** | 4.94E-18 |
| | | **TGM2** | Transglutaminase 2 | down | **-2.40** | 1.64E-04 |
| | | **ANKRD1** | Ankyrin repeat domain 1 | down | **-1.79** | 1.02E-02 |
| | | **SFRP2** | Secreted frizzled related protein 2 | down | **-1.78** | 2.39E-03 |
| | | **IGFBP3** | Insulin like growth factor binding protein 3 | down | **-1.64** | 1.77E-03 |
| | | **NTRK3** | Neurotrophic receptor tyrosine kinase 3 | down | **-1.61** | 4.88E-04 |
| **Response to drug** | GO:0042493 | **DUSP6** | Dual specificity phosphatase 6 | up | **1.82** | 2.93E-03 |
| | | **SOD2** | Superoxide dismutase 2, mitochondrial | up | **1.69** | 1.35E-18 |
| | | **TGFBR2** | Transforming growth factor beta receptor 2 | up | **1.64** | 2.09E-18 |
| | | **PLIN2** | Perilipin 2 | up | **1.60** | 3.19E-14 |
| | | **INHBA** | Inhibin beta A subunit | down | **-2.07** | 3.31E-03 |
| | | **SFRP2** | Secreted frizzled related protein 2 | down | **-1.78** | 2.39E-03 |
| | | **SST** | Somatostatin | down | **-1.69** | 3.85E-03 |
| | | **COL1A1** | Collagen type I alpha 1 chain | down | **-1.68** | 3.34E-03 |
| **Response to hyperoxia** | GO:0055093 | **SOD2** | Superoxide dismutase 2, mitochondrial | up | **1.69** | 1.35E-18 |
| | | **KCNA5** | Potassium voltage-gated channel subfamily A member 5 | up | **1.57** | 6.84E-17 |

| | | | | | | |
|---|---|---|---|---|---|---|
| | | **COL1A1** | Collagen type I alpha 1 chain | down | **-1.68** | 3.34E-03 |
| | | | | | | |
| **Action potential** | GO:0001508 | **USP53** | Ubiquitin specific peptidase 53 | up | **1.73** | 1.71E-40 |
| | | **KCND2** | Potassium voltage-gated channel subfamily D member 2 | down | **-1.74** | 8.81E-05 |
| | | **GJD2** | Gap junction protein delta 2 | down | **-1.70** | 8.95E-05 |
| | | | | | | |
| **Osteoblast differentiation** | GO:0001649 | **WNT11** | Wnt family member 11 | up | **1.61** | 3.74E-04 |
| | | **ITGA11** | Integrin subunit alpha 11 | down | **-2.24** | 3.58E-07 |
| | | **SPP1** | Secreted phosphoprotein 1 | down | **-2.25** | 5.38E-05 |
| | | **COL1A1** | Collagen type I alpha 1 chain | down | **-1.68** | 3.34E-03 |
| | | **IGFBP3** | Insulin like growth factor binding protein 3 | down | **-1.64** | 1.77E-03 |
| | | | | | | |
| **Ganglioside biosynthetic process** | GO:0001574 | **ST6GALNAC2** | ST6 N-acetylgalactosaminide alpha-2,6-sialyltransferase 2 | up | **2.24** | 8.46E-132 |
| | | **ST3GAL5** | ST3 beta-galactoside alpha-2,3-sialyltransferase 5 | up | **1.72** | 1.01E-12 |
| | | **B4GALNT1** | Beta-1,4-N-acetyl-galactosaminyltransferase 1 | up | **1.51** | 1.60E-02 |
| | | | | | | |
| **Activation of MAPK activity** | GO:0000187 | **DUSP5** | Dual specificity phosphatase 5 | up | **1.84** | 3.95E-03 |
| | | **CXCR4** | C-X-C motif chemokine receptor 4 | up | **1.81** | 5.67E-15 |
| | | **DUSP6** | Dual specificity phosphatase 6 | up | **1.82** | 2.93E-03 |
| | | **IGF1** | Insulin like growth factor 1 | down | **-1.85** | 1.51E-18 |
| | | **NTRK3** | Neurotrophic receptor tyrosine kinase 3 | down | **-1.61** | 4.88E-04 |
| | | | | | | |
| **Cellular response to tumor necrosis factor** | GO:0071356 | **ZFP36** | ZFP36 ring finger protein | up | **1.58** | 1.58E-04 |
| | | **INPP5K** | Inositol polyphosphate-5-phosphatase K | up | **1.57** | 3.18E-28 |
| | | **CD58** | CD58 molecule | up | **1.51** | 1.09E-05 |
| | | **ANKRD1** | Ankyrin repeat domain 1 | down | **-1.79** | 1.02E-02 |

| | | | | | | |
|---|---|---|---|---|---|---|
| | | **COL1A1** | Collagen type I alpha 1 chain | down | **-1.68** | 3.34E-03 |
| **Response to steroid hormone** | GO:0048545 | **TGFBR2** | Transforming growth factor beta receptor 2 | up | **1.64** | 2.09E-18 |
| | | **SPP1** | Secreted phosphoprotein 1 | down | **-2.25** | 5.38E-05 |
| | | **SST** | Somatostatin | down | **-1.69** | 3.85E-03 |
| **Positive regulation of smooth muscle cell proliferation** | GO:0048661 | **SERPINF2** | Serpin family F member 2 | up | **1.70** | 2.01E-40 |
| | | **TGFBR2** | Transforming growth factor beta receptor 2 | up | **1.64** | 2.09E-18 |
| | | **TGM2** | Transglutaminase 2 | down | **-2.40** | 1.64E-04 |
| | | **IGF1** | Insulin like growth factor 1 | down | **-1.85** | 1.51E-18 |
| **Peptidyl-serine phosphorylation** | GO:0018105 | **RPS6KA2** | Ribosomal protein S6 kinase A2 | up | **1.79** | 4.35E-45 |
| | | **TGFBR2** | Transforming growth factor beta receptor 2 | up | **1.64** | 2.09E-18 |
| | | **SGK1** | Serum/glucocorticoid regulated kinase 1 | up | **1.57** | 8.43E-35 |
| | | **STK32B** | Serine/threonine kinase 32B | down | **-1.55** | 1.49E-02 |
| | | **TTK** | TTK protein kinase | down | **-1.54** | 3.29E-03 |
| **Cell adhesion** | GO:0007155 | **EPHA8** | EPH receptor A8 | up | **2.38** | 4.21E-20 |
| | | **ITGB4** | Integrin subunit beta 4 | up | **1.60** | 8.17E-52 |
| | | **CD58** | CD58 molecule | up | **1.51** | 1.09E-05 |
| | | **SPP1** | Secreted phosphoprotein 1 | down | **-2.25** | 5.38E-05 |
| | | **ITGA11** | Integrin subunit alpha 11 | down | **-2.24** | 3.58E-07 |
| | | **AMTN** | Amelotin | down | **-2.08** | 1.63E-06 |
| | | **OMG** | Oligodendrocyte myelin glycoprotein | down | **-2.02** | 2.94E-05 |
| | | **COL1A1** | Collagen type I alpha 1 chain | down | **-1.68** | 3.34E-03 |
| | | **ROBO2** | Roundabout guidance receptor 2 | down | **-1.61** | 1.18E-10 |
| **Regulation of endodermal cell fate specification** | GO:0042663 | **DUSP6** | Dual specificity phosphatase 6 | up | **1.82** | 2.93E-03 |
| | | **DKK1** | Dickkopf WNT signaling pathway inhibitor 1 | up | **1.76** | 5.76E-04 |

| | | | | | | |
|---|---|---|---|---|---|---|
| **Cellular response to epidermal growth factor stimulus** | GO:0071364 | **ZFP36** | ZFP36 ring finger protein | up | **1.58** | 1.58E-04 |
| | | **INPP5K** | Inositol polyphosphate-5-phosphatase K | up | **1.57** | 3.18E-28 |
| | | **COL1A1** | Collagen type I alpha 1 chain | down | **-1.68** | 3.34E-03 |
| **Oxidation-reduction process** | GO:0055114 | **MAOA** | Monoamine oxidase A | up | **1.94** | 3.65E-09 |
| | | **KIAA1456** | KIAA1456 | up | **1.92** | 2.89E-22 |
| | | **HPD** | 4-hydroxyphenylpyruvate dioxygenase | up | **1.91** | 1.93E-34 |
| | | **SH3PXD2B** | SH3 and PX domains 2B | up | **1.88** | 1.13E-22 |
| | | **ALDH1L1** | Aldehyde dehydrogenase 1 family member L1 | up | **1.73** | 1.10E-11 |
| | | **CP** | Ceruloplasmin | up | **1.70** | 8.53E-12 |
| | | **PRODH** | Proline dehydrogenase 1 | up | **1.69** | 6.92E-91 |
| | | **SOD2** | Superoxide dismutase 2, mitochondrial | up | **1.69** | 1.35E-18 |
| | | **CRYZ** | Crystallin zeta | up | **1.64** | 3.83E-80 |
| | | **RRM2** | Ribonucleotide reductase regulatory subunit M2 | down | **-1.55** | 1.46E-08 |
| **Negative regulation of mesodermal cell fate specification** | GO:0042662 | **DKK1** | Dickkopf WNT signaling pathway inhibitor 1 | up | **1.76** | 5.76E-04 |
| | | **SFRP2** | secreted frizzled related protein 2 | down | **-1.78** | 2.39E-03 |
| **Transmembrane transport** | GO:0055085 | **SLC13A4** | Solute carrier family 13 member 4 | up | **2.03** | 3.40E-95 |
| | | **SLC16A12** | Solute carrier family 16 member 12 | up | **1.96** | 6.25E-39 |
| | | **ABCC5** | ATP binding cassette subfamily C member 5 | up | **1.75** | 3.28E-22 |
| | | **ABCA4** | ATP binding cassette subfamily A member 4 | up | **1.69** | 4.72E-11 |
| | | **SLC16A10** | Solute carrier family 16 member 10 | up | **1.57** | 1.76E-30 |
| | | **GJD2** | Gap junction protein delta 2 | down | **-1.70** | 8.95E-05 |
| **Collagen fibril organization** | GO:0030199 | **SERPINF2** | Serpin family F member 2 | up | **1.70** | 2.01E-40 |
| | | **SFRP2** | Secreted frizzled related protein 2 | down | **-1.78** | 2.39E-03 |
| | | **COL1A1** | Collagen type I alpha 1 chain | down | **-1.68** | 3.34E-03 |

| | | | | | | |
|---|---|---|---|---|---|---|
| **Embryo implantation** | GO:0007566 | **PRLR** | Prolactin receptor | up | **2.01** | 9.89E-34 |
| | | **TGFBR2** | Transforming growth factor beta receptor 2 | up | **1.64** | 2.09E-18 |
| | | **SPP1** | Secreted phosphoprotein 1 | down | **-2.25** | 5.38E-05 |
| **Regulation of cell adhesion** | GO:0030155 | **EPHA8** | EPH receptor A8 | up | **2.38** | 4.21E-20 |
| | | **PRLR** | Prolactin receptor | up | **2.01** | 9.89E-34 |
| | | **CYTIP** | Cytohesin 1 interacting protein | up | **1.53** | 2.87E-03 |
| **Convergent extension involved in axis elongation** | GO:0060028 | **WNT11** | Wnt family member 11 | up | **1.61** | 3.74E-04 |
| | | **SFRP2** | Secreted frizzled related protein 2 | down | **-1.78** | 2.39E-03 |
| **Substrate-dependent cell migration** | GO:0006929 | **EPHA8** | EPH receptor A8 | up | **2.38** | 4.21E-20 |
| | | **ITGA11** | Integrin subunit alpha 11 | down | **-2.24** | 3.58E-07 |
| **Outflow tract morphogenesis** | GO:0003151 | **TGFBR2** | Transforming growth factor beta receptor 2 | up | **1.64** | 2.09E-18 |
| | | **WNT11** | Wnt family member 11 | up | **1.61** | 3.74E-04 |
| | | **SFRP2** | Secreted frizzled related protein 2 | down | **-1.78** | 2.39E-03 |
| **Heart development** | GO:0007507 | **SH3PXD2B** | SH3 and PX domains 2B | up | **1.88** | 1.13E-22 |
| | | **RPS6KA2** | Ribosomal protein S6 kinase A2 | up | **1.79** | 4.35E-45 |
| | | **SOD2** | Superoxide dismutase 2, mitochondrial | up | **1.69** | 1.35E-18 |
| | | **TGFBR2** | Transforming growth factor beta receptor 2 | up | **1.64** | 2.09E-18 |
| | | **NTRK3** | Neurotrophic receptor tyrosine kinase 3 | down | **-1.61** | 4.88E-04 |
| **Regulation of cell proliferation** | GO:0042127 | **PTGER2** | Prostaglandin E receptor 2 | up | **2.62** | 5.28E-54 |
| | | **TGFBR2** | Transforming growth factor beta receptor 2 | up | **1.64** | 2.09E-18 |
| | | **SGK1** | Serum/glucocorticoid regulated kinase 1 | up | **1.57** | 8.43E-35 |
| | | **CHST11** | Carbohydrate sulfotransferase 11 | up | **1.55** | 1.45E-25 |

| | | | | | | |
|---|---|---|---|---|---|---|
| | | **CDCA7** | Cell division cycle associated 7 | down | **-1.71** | 2.48E-04 |
| | | | | | | |
| **Positive regulation of fat cell differentiation** | GO:0045600 | **SH3PXD2B** | SH3 and PX domains 2B | up | **1.88** | 1.13E-22 |
| | | **ZFP36** | ZFP36 ring finger protein | up | **1.58** | 1.58E-04 |
| | | **SFRP2** | Secreted frizzled related protein 2 | down | **-1.78** | 2.39E-03 |
| | | | | | | |
| **Proton transport** | GO:0015992 | **HVCN1** | Hydrogen voltage gated channel 1 | up | **4.15** | 4.71E-32 |
| | | **SLC2A12** | Solute carrier family 2 member 12 | up | **1.58** | 2.58E-42 |
| | | **ATP6V0A4** | ATPase $H^+$ transporting V0 subunit a4 | up | **1.58** | 1.75E-05 |

GO, gene ontology; up, up-regulated genes in the biological process gene ontology term; down, down-regulated genes in the biological process gene ontology term; DEG, differentially expressed gene; FC, fold-change.

**Supplementary Table S13.** Specific cortisol + RU-486-regulated enriched biological process gene ontology terms as predicted by bioinformatic enrichment analysis.

**GO Biological Process** analysis on DEGs

**Cortisol $10^{-7}$ M + RU-486 $10^{-5}$ M *versus* Control**

| GO biological process term | Link to GO ID | Gene name | Description | Regulation | FC | *P*-value |
|---|---|---|---|---|---|---|
| **Mitotic nuclear division** | GO:0007067 | **CCNA1** | Cyclin A1 | down | **-1.92** | 1.29E-07 |
| | | **SPC24** | SPC24, NDC80 kinetochore complex component | down | **-1.84** | 4.74E-07 |
| | | **BUB1B** | BUB1 mitotic checkpoint serine/threonine kinase B | down | **-1.81** | 2.76E-11 |
| | | **NEK2** | NIMA related kinase 2 | down | **-1.77** | 2.50E-03 |
| | | **PLK1** | Polo like kinase 1 | down | **-1.72** | 2.43E-04 |
| | | **CCNA2** | Cyclin A2 | down | **-1.72** | 1.60E-07 |
| | | **CENPF** | Centromere protein F | down | **-1.63** | 2.37E-05 |
| | | **CDK1** | Cyclin dependent kinase 1 | down | **-1.62** | 4.47E-10 |
| | | **TPX2** | TPX2, microtubule nucleation factor | down | **-1.60** | 1.84E-10 |
| | | **CDCA3** | Cell division cycle associated 3 | down | **-1.59** | 4.27E-03 |
| | | **ASPM** | Abnormal spindle microtubule assembly | down | **-1.58** | 1.97E-06 |
| | | **ANLN** | Anillin actin binding protein | down | **-1.58** | 1.99E-06 |
| | | **SPC25** | SPC25, NDC80 kinetochore complex component | down | **-1.54** | 3.80E-02 |
| | | **CDC20** | Cell division cycle 20 | down | **-1.52** | 1.12E-03 |
| | | | | | | |
| **Response to drug** | GO:0042493 | **FOSL1** | FOS like 1, AP-1 transcription factor subunit | up | **3.81** | 3.16E-08 |
| | | **DUSP6** | Dual specificity phosphatase 6 | up | **2.85** | 1.83E-07 |
| | | **PLIN2** | Perilipin 2 | up | **2.83** | 3.52E-64 |
| | | **ANXA1** | Annexin A1 | up | **1.93** | 1.17E-08 |
| | | **IL6** | Interleukin 6 | up | **1.76** | 3.15E-05 |
| | | **CPT1A** | Carnitine palmitoyltransferase 1A | up | **1.67** | 3.98E-52 |
| | | **MGST1** | Microsomal glutathione S-transferase 1 | up | **1.57** | 3.47E-18 |
| | | **SOD2** | Superoxide dismutase 2, mitochondrial | up | **1.52** | 2.06E-12 |
| | | **INHBA** | Inhibin beta A subunit | down | **-1.96** | 6.23E-03 |

| | | | | | | |
|---|---|---|---|---|---|---|
| | | **OXTR** | Oxytocin receptor | down | **-1.85** | 1.73E-15 |
| | | **COL1A1** | Collagen type I alpha 1 chain | down | **-1.64** | 4.98E-03 |
| | | **CENPF** | Centromere protein F | down | **-1.63** | 2.37E-05 |
| | | **CDK1** | Cyclin dependent kinase 1 | down | **-1.62** | 4.47E-10 |
| | | **SST** | Somatostatin | down | **-1.60** | 8.94E-03 |
| | | **CA9** | Carbonic anhydrase 9 | down | **-1.50** | 7.90E-24 |
| | | | | | | |
| **Positive regulation of apoptotic process** | GO:0043065 | **FOSL1** | FOS like 1, AP-1 transcription factor subunit | up | **3.81** | 3.16E-08 |
| | | **DUSP6** | dual specificity phosphatase 6 | up | **2.85** | 1.83E-07 |
| | | **KCNMA1** | Potassium calcium-activated channel subfamily M alpha 1 | up | **1.92** | 6.93E-10 |
| | | **IL6** | Interleukin 6 | up | **1.76** | 3.15E-05 |
| | | **OSGIN1** | Oxidative stress induced growth inhibitor 1 | up | **1.74** | 1.43E-08 |
| | | **PMAIP1** | Phorbol-12-myristate-13-acetate-induced protein 1 | up | **1.61** | 1.21E-06 |
| | | **CYP1B1** | Cytochrome P450 family 1 subfamily B member 1 | up | **1.55** | 3.67E-05 |
| | | **RPS6KA2** | Ribosomal protein S6 kinase A2 | up | **1.54** | 6.36E-26 |
| | | **TGM2** | Transglutaminase 2 | down | **-2.86** | 6.47E-06 |
| | | **FGD3** | FYVE, RhoGEF and PH domain containing 3 | down | **-2.16** | 4.81E-09 |
| | | **NTRK3** | Neurotrophic receptor tyrosine kinase 3 | down | **-1.97** | 1.20E-06 |
| | | **ANKRD1** | Ankyrin repeat domain 1 | down | **-1.85** | 6.52E-03 |
| | | **BMF** | Bcl2 modifying factor | down | **-1.69** | 8.82E-13 |
| | | **TOP2A** | Topoisomerase (DNA) II alpha | down | **-1.56** | 2.82E-06 |
| | | | | | | |
| **Response to organic cyclic compound** | GO:0014070 | **DUSP6** | Dual specificity phosphatase 6 | up | **2.85** | 1.83E-07 |
| | | **PLIN2** | Perilipin 2 | up | **2.83** | 3.52E-64 |
| | | **CPT1A** | Carnitine palmitoyltransferase 1A | up | **1.67** | 3.98E-52 |
| | | **LUM** | Lumican | down | **-1.72** | 6.80E-05 |
| | | **MKI67** | Marker of proliferation Ki-67 | down | **-1.69** | 6.36E-05 |
| | | **CDK1** | Cyclin dependent kinase 1 | down | **-1.62** | 4.47E-10 |
| | | | | | | |
| | GO:0043066 | **ANGPTL4** | Angiopoietin like 4 | up | **3.56** | 2.07E-31 |

| | | | | | | |
|---|---|---|---|---|---|---|
| Negative regulation of apoptotic process | | **PLAUR** | Plasminogen activator, urokinase receptor | up | **2.38** | 1.14E-10 |
| | | **ANXA1** | Annexin A1 | up | **1.93** | 1.17E-08 |
| | | **SPRY2** | Sprouty RTK signaling antagonist 2 | up | **1.77** | 3.61E-07 |
| | | **IL6** | Interleukin 6 | up | **1.76** | 3.15E-05 |
| | | **NQO1** | NAD(P)H quinone dehydrogenase 1 | up | **1.72** | 9.60E-09 |
| | | **PRLR** | Prolactin receptor | up | **1.66** | 1.57E-18 |
| | | **TNFSF18** | Tumor necrosis factor superfamily member 18 | up | **1.59** | 8.30E-04 |
| | | **CD44** | CD44 molecule (Indian blood group) | up | **1.54** | 2.73E-09 |
| | | **SOD2** | Superoxide dismutase 2, mitochondrial | up | **1.52** | 2.06E-12 |
| | | **CHST11** | Carbohydrate sulfotransferase 11 | up | **1.50** | 2.25E-22 |
| | | **TGM2** | Transglutaminase 2 | down | **-2.86** | 6.47E-06 |
| | | **SOX8** | SRY-box 8 | down | **-1.97** | 1.41E-07 |
| | | **PLK1** | Polo like kinase 1 | down | **-1.72** | 2.43E-04 |
| | | **CDK1** | Cyclin dependent kinase 1 | down | **-1.62** | 4.47E-10 |
| | | **CCND2** | Cyclin D2 | down | **-1.54** | 2.85E-17 |
| | | | | | | |
| **Collagen fibril organization** | GO:0030199 | **CYP1B1** | Cytochrome P450 family 1 subfamily B member 1 | up | **1.55** | 3.67E-05 |
| | | **MMP11** | Matrix metallopeptidase 11 | down | **-1.76** | 9.27E-23 |
| | | **LUM** | Lumican | down | **-1.72** | 6.80E-05 |
| | | **COL1A1** | Collagen type I alpha 1 chain | down | **-1.64** | 4.98E-03 |
| | | **COL3A1** | Collagen type III alpha 1 chain | down | **-1.52** | 1.44E-13 |
| | | | | | | |
| **Negative regulation of fat cell differentiation** | GO:0045599 | **IL6** | Interleukin 6 | up | **1.76** | 3.15E-05 |
| | | **TRIB3** | Tribbles pseudokinase 3 | up | **1.71** | 2.94E-06 |
| | | **SOD2** | Superoxide dismutase 2, mitochondrial | up | **1.52** | 2.06E-12 |
| | | **RUNX1T1** | RUNX1 translocation partner 1 | down | **-1.93** | 1.02E-05 |
| | | **MMP11** | Matrix metallopeptidase 11 | down | **-1.76** | 9.27E-23 |
| | | | | | | |
| **Sister chromatid cohesion** | GO:0007062 | **SPC24** | SPC24, NDC80 kinetochore complex component | down | **-1.84** | 4.74E-07 |
| | | **BUB1B** | BUB1 mitotic checkpoint serine/threonine Kinase B | down | **-1.81** | 2.76E-11 |

| | | | | | | |
|---|---|---|---|---|---|---|
| | | **PLK1** | Polo like kinase 1 | down | **-1.72** | 2.43E-04 |
| | | **CENPF** | Centromere protein F | down | **-1.63** | 2.37E-05 |
| | | **CENPM** | Centromere protein M | down | **-1.58** | 5.36E-03 |
| | | **SPC25** | SPC25, NDC80 kinetochore complex component | down | **-1.54** | 3.80E-02 |
| | | **CDC20** | Cell division cycle 20 | down | **-1.52** | 1.12E-03 |
| | | | | | | |
| **Cell division** | GO:0051301 | **CCNA1** | Cyclin A1 | down | **-1.92** | 1.29E-07 |
| | | **SPC24** | SPC24, NDC80 kinetochore complex component | down | **-1.84** | 4.74E-07 |
| | | **BUB1B** | BUB1 mitotic checkpoint serine/threonine kinase B | down | **-1.81** | 2.76E-11 |
| | | **NEK2** | NIMA related kinase 2 | down | **-1.77** | 2.50E-03 |
| | | **CCNA2** | Cyclin A2 | down | **-1.72** | 1.60E-07 |
| | | **CENPF** | Centromere protein F | down | **-1.63** | 2.37E-05 |
| | | **CDK1** | Cyclin dependent kinase 1 | down | **-1.62** | 4.47E-10 |
| | | **TPX2** | TPX2, microtubule nucleation factor | down | **-1.60** | 1.84E-10 |
| | | **CDCA3** | Cell division cycle associated 3 | down | **-1.59** | 4.27E-03 |
| | | **SPC25** | SPC25, NDC80 kinetochore complex component | down | **-1.54** | 3.80E-02 |
| | | **CCND2** | Cyclin D2 | down | **-1.54** | 2.85E-17 |
| | | **CDC20** | Cell division cycle 20 | down | **-1.52** | 1.12E-03 |
| | | **KIFC1** | Kinesin family member C1 | down | **-1.50** | 4.13E-05 |
| | | | | | | |
| **Response to cAMP** | GO:0051591 | **AGXT** | Alanine-glyoxylate aminotransferase | up | **5.02** | 6.09E-05 |
| | | **FOSL1** | FOS like 1, AP-1 transcription factor subunit | up | **3.81** | 3.16E-08 |
| | | **PTPRN** | protein tyrosine phosphatase, receptor type N | up | **1.73** | 2.71E-05 |
| | | **PER1** | Period circadian clock 1 | up | **1.51** | 1.76E-11 |
| | | **COL1A1** | Collagen type I alpha 1 chain | down | **-1.64** | 4.98E-03 |
| | | | | | | |
| **Microtubule bundle formation** | GO:0001578 | **KIF20A** | Kinesin family member 20A | down | **-2.04** | 5.94E-08 |
| | | **TPPP3** | Tubulin polymerization promoting protein family member 3 | down | **-1.73** | 5.85E-04 |
| | | **PLK1** | Polo like kinase 1 | down | **-1.72** | 2.43E-04 |

| | | | | | | |
|---|---|---|---|---|---|---|
| | | **CAPN6** | Calpain 6 | down | **-1.67** | 3.35E-12 |
| | | | | | | |
| **Cell adhesion** | GO:0007155 | **EFCAB13** | EF-hand calcium binding domain 13 | up | **1.94** | 2.60E-06 |
| | | **LAMC2** | Laminin subunit gamma 2 | up | **1.76** | 5.04E-12 |
| | | **ATP1B2** | ATPase $Na^+/K^+$ transporting subunit beta 2 | up | **1.57** | 1.62E-10 |
| | | **CYP1B1** | Cytochrome P450 family 1 subfamily B member 1 | up | **1.55** | 3.67E-05 |
| | | **CD44** | CD44 molecule (Indian blood group) | up | **1.54** | 2.73E-09 |
| | | **EPHA8** | EPH receptor A8 | up | **1.50** | 2.43E-05 |
| | | **OMG** | Oligodendrocyte myelin glycoprotein | down | **-2.80** | 1.53E-09 |
| | | **AMTN** | Amelotin | down | **-2.37** | 1.93E-08 |
| | | **ITGA11** | Integrin subunit alpha 11 | down | **-2.19** | 7.41E-07 |
| | | **HAPLN3** | Hyaluronan and proteoglycan link protein 3 | down | **-2.00** | 8.82E-05 |
| | | **MOG** | Myelin oligodendrocyte glycoprotein | down | **-1.73** | 2.64E-02 |
| | | **COL1A1** | Collagen type I alpha 1 chain | down | **-1.64** | 4.98E-03 |
| | | **MFAP4** | Microfibrillar associated protein 4 | down | **-1.55** | 2.66E-07 |
| | | **THBS2** | Thrombospondin 2 | down | **-1.53** | 2.41E-06 |
| | | **MFGE8** | Milk fat globule-EGF factor 8 protein | down | **-1.52** | 9.70E-30 |
| | | | | | | |
| **Negative regulation of cell adhesion mediated by integrin** | GO:0033629 | **ACER2** | Alkaline ceramidase 2 | up | **1.67** | 2.27E-02 |
| | | **CYP1B1** | Cytochrome P450 family 1 subfamily B member 1 | up | **1.55** | 3.67E-05 |
| | | **SERPINE1** | Serpin family E member 1 | up | **1.54** | 2.46E-03 |
| | | | | | | |
| **Extracellular matrix organization** | GO:0030198 | **EFCAB13** | EF-hand calcium binding domain 13 | up | **1.94** | 2.60E-06 |
| | | **LAMC2** | Laminin subunit gamma 2 | up | **1.76** | 5.04E-12 |
| | | **SERPINE1** | Serpin family E member 1 | up | **1.54** | 2.46E-03 |
| | | **CD44** | CD44 molecule (Indian blood group) | up | **1.54** | 2.73E-09 |
| | | **ELF3** | E74 like ETS transcription factor 3 | down | **-2.34** | 1.44E-08 |
| | | **ITGA11** | Integrin subunit alpha 11 | down | **-2.19** | 7.41E-07 |
| | | **LUM** | Lumican | down | **-1.72** | 6.80E-05 |
| | | **COL1A1** | Collagen type I alpha 1 chain | down | **-1.64** | 4.98E-03 |
| | | **COL3A1** | Collagen type III alpha 1 chain | down | **-1.52** | 1.44E-13 |

| | | | | | | |
|---|---|---|---|---|---|---|
| **Positive regulation of ubiquitin protein ligase activity** | GO:1904668 | **CDC20B** | Cell division cycle 20B | down | **-1.85** | 3.55E-03 |
| | | **PLK1** | Polo like kinase 1 | down | **-1.72** | 2.43E-04 |
| | | **CDC20** | Cell division cycle 20 | down | **-1.52** | 1.12E-03 |
| **Negative regulation of platelet aggregation** | GO:0090331 | **SERPINE2** | Serpin family E member 2 | up | **2.12** | 3.52E-09 |
| | | **C1QTNF1** | C1q and tumor necrosis factor related protein 1 | up | **1.56** | 9.15E-14 |
| | | **UBASH3B** | Ubiquitin associated and SH3 domain containing B | up | **1.51** | 1.04E-03 |
| **Mitotic cytokinesis** | GO:0000281 | **KIF20A** | Kinesin family member 20A | down | **-2.04** | 5.94E-08 |
| | | **PLK1** | Polo like kinase 1 | down | **-1.72** | 2.43E-04 |
| | | **KIF4A** | Kinesin family member 4A | down | **-1.71** | 6.57E-05 |
| | | **ANLN** | Anillin actin binding protein | down | **-1.58** | 1.99E-06 |
| **Negative regulation of cell proliferation** | GO:0008285 | **FOSL1** | FOS like 1, AP-1 transcription factor subunit | up | **3.81** | 3.16E-08 |
| | | **SERPINE2** | Serpin family E member 2 | up | **2.12** | 3.52E-09 |
| | | **HMGA1** | High mobility group AT-hook 1 | up | **1.97** | 7.42E-07 |
| | | **SPRY2** | Sprouty RTK signaling antagonist 2 | up | **1.77** | 3.61E-07 |
| | | **IL6** | Interleukin 6 | up | **1.76** | 3.15E-05 |
| | | **TESC** | Tescalcin | up | **1.69** | 7.70E-06 |
| | | **IFITM1** | Interferon induced transmembrane protein 1 | up | **1.59** | 2.00E-05 |
| | | **CYP1B1** | Cytochrome P450 family 1 subfamily B member 1 | up | **1.55** | 3.67E-05 |
| | | **RPS6KA2** | Ribosomal protein S6 kinase A2 | up | **1.54** | 6.36E-26 |
| | | **SOD2** | Superoxide dismutase 2, mitochondrial | up | **1.52** | 2.06E-12 |
| | | **INHBA** | Inhibin beta A subunit | down | **-1.96** | 6.23E-03 |
| | | **SST** | Somatostatin | down | **-1.60** | 8.94E-03 |
| | | **CDKN2C** | Cyclin dependent kinase inhibitor 2C | down | **-1.57** | 1.97E-04 |
| **Positive regulation of gene expression** | GO:0010628 | **IL7R** | Interleukin 7 receptor | up | **3.40** | 3.11E-08 |

| | | | | | | |
|---|---|---|---|---|---|---|
| | | **SPRY2** | Sprouty RTK signaling antagonist 2 | up | **1.77** | 3.61E-07 |
| | | **IL6** | Interleukin 6 | up | **1.76** | 3.15E-05 |
| | | **TESC** | Tescalcin | up | **1.69** | 7.70E-06 |
| | | **C1QTNF1** | C1q and tumor necrosis factor related protein 1 | up | **1.56** | 9.15E-14 |
| | | **RPS6KA2** | Ribosomal protein S6 kinase A2 | up | **1.54** | 6.36E-26 |
| | | **ACTA2** | Actin, alpha 2, smooth muscle, aorta | down | **-2.13** | 4.92E-19 |
| | | **NTRK3** | Neurotrophic receptor tyrosine kinase 3 | down | **-1.97** | 1.20E-06 |
| | | **INHBA** | Inhibin beta A subunit | down | **-1.96** | 6.23E-03 |
| | | **CDK1** | Cyclin dependent kinase 1 | down | **-1.62** | 4.47E-10 |
| | | | | | | |
| **Protein homooligomerization** | GO:0051260 | **AKR1C1** | Aldo-keto reductase family 1 member C1 | up | **16.20** | 8.08E-17 |
| | | **ANGPTL4** | Angiopoietin like 4 | up | **3.56** | 2.07E-31 |
| | | **CPT1A** | Carnitine palmitoyltransferase 1A | up | **1.67** | 3.98E-52 |
| | | **SLC22A6** | Solute carrier family 22 member 6 | up | **1.63** | 1.17E-05 |
| | | **C1QTNF1** | C1q and tumor necrosis factor related protein 1 | up | **1.56** | 9.15E-14 |
| | | **KCNA5** | Potassium voltage-gated channel subfamily A member 5 | up | **1.53** | 3.15E-15 |
| | | **SOD2** | Superoxide dismutase 2, mitochondrial | up | **1.52** | 2.06E-12 |
| | | **TGM2** | Transglutaminase 2 | down | **-2.86** | 6.47E-06 |
| | | | | | | |
| **Positive regulation of cell proliferation** | GO:0008284 | **FOSL1** | FOS like 1, AP-1 transcription factor subunit | up | **3.81** | 3.16E-08 |
| | | **IL6** | Interleukin 6 | up | **1.76** | 3.15E-05 |
| | | **HILPDA** | Hypoxia inducible lipid droplet associated | up | **1.76** | 2.88E-09 |
| | | **LAMC2** | Laminin subunit gamma 2 | up | **1.76** | 5.04E-12 |
| | | **ACER2** | Alkaline ceramidase 2 | up | **1.67** | 2.27E-02 |
| | | **OSMR** | Oncostatin M receptor | up | **1.53** | 4.39E-10 |
| | | **NTRK3** | Neurotrophic receptor tyrosine kinase 3 | down | **-1.97** | 1.20E-06 |
| | | **FGFR3** | Fibroblast growth factor receptor 3 | down | **-1.91** | 5.84E-19 |
| | | **FOXM1** | Forkhead box M1 | down | **-1.62** | 3.17E-03 |
| | | **MLXIPL** | MLX interacting protein like | down | **-1.56** | 3.07E-04 |
| | | **EDN2** | Endothelin 2 | down | **-1.54** | 4.96E-12 |

| | | | | | | |
|---|---|---|---|---|---|---|
| | | **CCND2** | Cyclin D2 | down | **-1.54** | 2.85E-17 |
| | | **CDC20** | Cell division cycle 20 | down | **-1.52** | 1.12E-03 |
| | | **NTN1** | Netrin 1 | down | **-1.51** | 3.14E-06 |
| **Mitotic spindle assembly** | GO:0090307 | **NEK2** | NIMA related kinase 2 | down | **-1.77** | 2.50E-03 |
| | | **TPX2** | TPX2, microtubule nucleation factor | down | **-1.60** | 1.84E-10 |
| | | **MYBL2** | MYB proto-oncogene like 2 | down | **-1.56** | 8.14E-06 |
| | | **KIFC1** | Kinesin family member C1 | down | **-1.50** | 4.13E-05 |
| **Epithelial cell differentiation** | GO:0030855 | **AKR1C1** | Aldo-keto reductase family 1 member C1 | up | **16.20** | 8.08E-17 |
| | | **CPT1A** | Carnitine palmitoyltransferase 1A | up | **1.67** | 3.98E-52 |
| | | **TAGLN** | Transgelin | down | **-2.35** | 7.00E-33 |
| | | **ELF3** | E74 like ETS transcription factor 3 | down | **-2.34** | 1.44E-08 |
| | | **CDK1** | Cyclin dependent kinase 1 | down | **-1.62** | 4.47E-10 |
| **Response to virus** | GO:0009615 | **FOSL1** | FOS like 1, AP-1 transcription factor subunit | up | **3.81** | 3.16E-08 |
| | | **HMGA1** | High mobility group AT-hook 1 | up | **1.97** | 7.42E-07 |
| | | **RSAD2** | Radical S-adenosyl methionine domain containing 2 | up | **1.74** | 3.08E-17 |
| | | **CXCR4** | C-X-C motif chemokine receptor 4 | up | **1.64** | 1.05E-10 |
| | | **IFITM1** | Interferon induced transmembrane protein 1 | up | **1.59** | 2.00E-05 |
| | | **ACTA2** | Actin, alpha 2, smooth muscle, aorta | down | **-2.13** | 4.92E-19 |
| **Regulation of cell migration** | GO:0030334 | **SERPINE2** | Serpin family E member 2 | up | **2.12** | 3.52E-09 |
| | | **EFCAB13** | EF-hand calcium binding domain 13 | up | **1.94** | 2.60E-06 |
| | | **CXCR4** | C-X-C motif chemokine receptor 4 | up | **1.64** | 1.05E-10 |
| | | **SST** | Somatostatin | down | **-1.60** | 8.94E-03 |
| | | **NTN1** | Netrin 1 | down | **-1.51** | 3.14E-06 |
| **Regulation of pH** | GO:0006885 | **PDK4** | Pyruvate dehydrogenase kinase 4 | up | **2.21** | 1.14E-10 |
| | | **SLC9A9** | Solute carrier family 9 member A9 | up | **1.61** | 3.20E-07 |

| | | | | | | |
|---|---|---|---|---|---|---|
| | | **ATP6V1B1** | ATPase $H^+$ transporting V1 subunit B1 | down | **-1.85** | 5.46E-07 |
| **Extracellular matrix disassembly** | GO:0022617 | **MMP1** | Matrix metallopeptidase 1 | up | **6.06** | 2.25E-08 |
| | | **LAMC2** | Laminin subunit gamma 2 | up | **1.76** | 5.04E-12 |
| | | **CD44** | CD44 molecule (Indian blood group) | up | **1.54** | 2.73E-09 |
| | | **ADAMTS5** | ADAM metallopeptidase with thrombospondin type 1 motif 5 | down | **-2.24** | 3.58E-08 |
| | | **MMP11** | Matrix metallopeptidase 11 | down | **-1.76** | 9.27E-23 |
| **Platelet activation** | GO:0030168 | **EFCAB13** | EF-hand calcium binding domain 13 | up | **1.94** | 2.60E-06 |
| | | **IL6** | Interleukin 6 | up | **1.76** | 3.15E-05 |
| | | **GNG2** | G protein subunit gamma 2 | up | **1.54** | 5.57E-13 |
| | | **ADRA2C** | Adrenoceptor alpha 2C | down | **-1.67** | 3.79E-20 |
| | | **COL1A1** | Collagen type I alpha 1 chain | down | **-1.64** | 4.98E-03 |
| | | **COL3A1** | Collagen type III alpha 1 chain | down | **-1.52** | 1.44E-13 |
| **Visual perception** | GO:0007601 | **PRCD** | Progressive rod-cone degeneration | up | **1.99** | 4.97E-04 |
| | | **RLBP1** | Retinaldehyde binding protein 1 | up | **1.57** | 6.70E-10 |
| | | **CYP1B1** | Cytochrome P450 family 1 subfamily B member 1 | up | **1.55** | 3.67E-05 |
| | | **ABCA4** | ATP binding cassette subfamily A member 4 | up | **1.54** | 5.36E-08 |
| | | **GJD2** | Gap junction protein delta 2 | down | **-3.92** | 6.10E-20 |
| | | **RGS9** | Regulator of G-protein signaling 9 | down | **-1.74** | 3.78E-06 |
| | | **LUM** | Lumican | down | **-1.72** | 6.80E-05 |
| | | **COL1A1** | Collagen type I alpha 1 chain | down | **-1.64** | 4.98E-03 |
| **Response to hyperoxia** | GO:0055093 | **KCNA5** | Potassium voltage-gated channel subfamily A member 5 | up | **1.53** | 3.15E-15 |
| | | **SOD2** | Superoxide dismutase 2, mitochondrial | up | **1.52** | 2.06E-12 |
| | | **COL1A1** | Collagen type I alpha 1 chain | down | **-1.64** | 4.98E-03 |

| | | | | | | |
|---|---|---|---|---|---|---|
| **Xenobiotic metabolic process** | GO:0006805 | **AKR1C1** | Aldo-keto reductase family 1 member C1 | up | **16.20** | 8.08E-17 |
| | | **NQO1** | NAD(P)H quinone dehydrogenase 1 | up | **1.72** | 9.60E-09 |
| | | **MGST1** | Microsomal glutathione S-transferase 1 | up | **1.57** | 3.47E-18 |
| | | **CYP1B1** | Cytochrome P450 family 1 subfamily B member 1 | up | **1.55** | 3.67E-05 |
| | | **CYP2W1** | Cytochrome P450 family 2 subfamily W member 1 | down | **-1.94** | 1.98E-11 |
| **Ganglioside biosynthetic process** | GO:0001574 | **B4GALNT1** | Beta-1,4-N-acetyl-galactosaminyltransferase 1 | up | **2.46** | 3.94E-08 |
| | | **ST3GAL5** | ST3 beta-galactoside alpha-2,3-sialyltransferase 5 | up | **1.75** | 1.86E-13 |
| | | **ST3GAL4** | ST3 beta-galactoside alpha-2,3-sialyltransferase 4 | down | **-1.54** | 1.14E-17 |
| **Positive regulation of MAPK cascade** | GO:0043410 | **IL6** | Interleukin 6 | up | **1.76** | 3.15E-05 |
| | | **C1QTNF1** | C1q and tumor necrosis factor related protein 1 | up | **1.56** | 9.15E-14 |
| | | **EPHA8** | EPH receptor A8 | up | **1.50** | 2.43E-05 |
| | | **GPR37L1** | G protein-coupled receptor 37 like 1 | down | **-2.01** | 2.81E-06 |
| | | **FGFR3** | Fibroblast growth factor receptor 3 | down | **-1.91** | 5.84E-19 |
| **Potassium ion transport** | GO:0006813 | **SLC24A4** | Solute carrier family 24 member 4 | up | **2.12** | 1.61E-05 |
| | | **KCNMA1** | Potassium calcium-activated channel subfamily M alpha 1 | up | **1.92** | 6.93E-10 |
| | | **ATP1B2** | ATPase $Na^+/K^+$ transporting subunit beta 2 | up | **1.57** | 1.62E-10 |
| | | **KCNA5** | Potassium voltage-gated channel subfamily A member 5 | up | **1.53** | 3.15E-15 |
| | | **KCNAB1** | Potassium voltage-gated channel subfamily A member regulatory beta subunit 1 | down | **-2.41** | 3.67E-12 |

| | | | | | | |
|---|---|---|---|---|---|---|
| **Positive regulation of ovulation** | GO:0060279 | **INHBA** | Inhibin beta A subunit | down | **-1.96** | 6.23E-03 |
| | | **INHBB** | Inhibin beta B subunit | down | **-1.68** | 4.33E-13 |
| | | | | | | |
| **Leukocyte migration** | GO:0050900 | **MMP1** | Matrix metallopeptidase 1 | up | **6.06** | 2.25E-08 |
| | | **EFCAB13** | EF-hand calcium binding domain 13 // integrin subunit beta 3 | up | **1.94** | 2.60E-06 |
| | | **ATP1B2** | ATPase $Na^+/K^+$ transporting subunit beta 2 | up | **1.57** | 1.62E-10 |
| | | **CD44** | CD44 molecule (Indian blood group) | up | **1.54** | 2.73E-09 |
| | | **SLC7A11** | Solute carrier family 7 member 11 | up | **1.52** | 6.66E-14 |
| | | **COL1A1** | Collagen type I alpha 1 chain | down | **-1.64** | 4.98E-03 |
| | | | | | | |
| **Defense response to virus** | GO:0051607 | **ISG15** | ISG15 ubiquitin-like modifier | up | **1.88** | 3.91E-04 |
| | | **IL6** | Interleukin 6 | up | **1.76** | 3.15E-05 |
| | | **RSAD2** | Radical S-adenosyl methionine domain containing 2 | up | **1.74** | 3.08E-17 |
| | | **IL33** | Interleukin 33 | up | **1.62** | 2.89E-15 |
| | | **PMAIP1** | Phorbol-12-myristate-13-acetate-induced protein 1 | up | **1.61** | 1.21E-06 |
| | | **IFITM1** | Interferon induced transmembrane protein 1 | up | **1.59** | 2.00E-05 |
| | | **UNC13D** | Unc-13 homolog D | down | **-1.55** | 1.57E-12 |
| | | | | | | |
| **Cell differentiation** | GO:0030154 | **ANGPTL4** | Angiopoietin like 4 | up | **3.56** | 2.07E-31 |
| | | **DUSP6** | Dual specificity phosphatase 6 | up | **2.85** | 1.83E-07 |
| | | **NDRG4** | NDRG family member 4 | up | **2.71** | 1.93E-12 |
| | | **OSGIN1** | Oxidative stress induced growth inhibitor 1 | up | **1.74** | 1.43E-08 |
| | | **ETV5** | ETS variant 5 | up | **1.55** | 4.11E-10 |
| | | **MGP** | Matrix Gla protein | down | **-2.21** | 5.47E-07 |
| | | **INHBA** | Inhibin beta A subunit | down | **-1.96** | 6.23E-03 |
| | | **ERF** | ETS2 repressor factor | down | **-1.88** | 1.18E-23 |
| | | **INHBB** | Inhibin beta B subunit | down | **-1.68** | 4.33E-13 |
| | | **CENPF** | Centromere protein F | down | **-1.63** | 2.37E-05 |
| | | **ABCB5** | ATP binding cassette subfamily B member 5 | down | **-1.59** | 3.70E-08 |

| | | | | | | |
|---|---|---|---|---|---|---|
| | | **MYBL2** | MYB proto-oncogene like 2 | down | **-1.56** | 8.14E-06 |
| | | **PLXNB3** | Plexin B3 | down | **-1.52** | 4.18E-11 |
| | | | | | | |
| **Regulation of vasoconstriction** | GO:0019229 | **KCNA5** | Potassium voltage-gated channel subfamily A member 5 | up | **1.53** | 3.15E-15 |
| | | **ADRA2C** | Adrenoceptor alpha 2C | down | **-1.67** | 3.79E-20 |
| | | **EDN2** | Endothelin 2 | down | **-1.54** | 4.96E-12 |
| | | | | | | |
| **Mitotic spindle assembly checkpoint** | GO:0007094 | **BUB1B** | BUB1 mitotic checkpoint serine/threonine kinase B | down | **-1.81** | 2.76E-11 |
| | | **PLK1** | Polo like kinase 1 | down | **-1.72** | 2.43E-04 |
| | | **CENPF** | Centromere protein F | down | **-1.63** | 2.37E-05 |
| | | | | | | |
| **Response to hydrogen peroxide** | GO:0042542 | **FOSL1** | FOS like 1, AP-1 transcription factor subunit | up | **3.81** | 3.16E-08 |
| | | **KCNA5** | Potassium voltage-gated channel subfamily A member 5 | up | **1.53** | 3.15E-15 |
| | | **SOD2** | Superoxide dismutase 2, mitochondrial | up | **1.52** | 2.06E-12 |
| | | **COL1A1** | Collagen type I alpha 1 chain | down | **-1.64** | 4.98E-03 |
| | | | | | | |
| **Positive regulation of protein localization to nucleus** | GO:1900182 | **PLK1** | Polo like kinase 1 | down | **-1.72** | 2.43E-04 |
| | | **CDK1** | Cyclin dependent kinase 1 | down | **-1.62** | 4.47E-10 |
| | | **GTSE1** | G2 and S-phase expressed 1 | down | **-1.51** | 6.60E-05 |
| | | | | | | |
| **Response to cytokine** | GO:0034097 | **FOSL1** | FOS like 1, AP-1 transcription factor subunit | up | **3.81** | 3.16E-08 |
| | | **OSMR** | Oncostatin M receptor | up | **1.53** | 4.39E-10 |
| | | **OXTR** | Oxytocin receptor | down | **-1.85** | 1.73E-15 |
| | | **COL3A1** | Collagen type III alpha 1 chain | down | **-1.52** | 1.44E-13 |
| | | | | | | |
| | GO:1900087 | **ANXA1** | Annexin A1 | up | **1.93** | 1.17E-08 |

| | | | | | | |
|---|---|---|---|---|---|---|
| **Positive regulation of G1/S transition of mitotic cell cycle** | | **KCNA5** | Potassium voltage-gated channel subfamily A member 5 | up | **1.53** | 3.15E-15 |
| | | **CCND2** | Cyclin D2 | down | **-1.54** | 2.85E-17 |
| **Cell surface receptor signaling pathway** | GO:0007166 | **IL7R** | Interleukin 7 receptor | up | **3.40** | 3.11E-08 |
| | | **ANXA1** | Annexin A1 | up | **1.93** | 1.17E-08 |
| | | **PRLR** | Prolactin receptor | up | **1.66** | 1.57E-18 |
| | | **IFITM1** | Interferon induced transmembrane protein 1 | up | **1.59** | 2.00E-05 |
| | | **TSPAN1** | Tetraspanin 1 | up | **1.52** | 1.87E-02 |
| | | **INHBA** | Inhibin beta A subunit | down | **-1.96** | 6.23E-03 |
| | | **OXTR** | Oxytocin receptor | down | **-1.85** | 1.73E-15 |
| | | **SST** | Somatostatin | down | **-1.60** | 8.94E-03 |
| | | **EDN2** | Endothelin 2 | down | **-1.54** | 4.96E-12 |
| **Regulation of ubiquitin-protein ligase activity involved in mitotic cell cycle** | GO:0051439 | **PLK1** | Polo like kinase 1 | down | **-1.72** | 2.43E-04 |
| | | **CDK1** | Cyclin dependent kinase 1 | down | **-1.62** | 4.47E-10 |
| | | **CDC20** | Cell division cycle 20 | down | **-1.52** | 1.12E-03 |
| **Negative regulation of follicle-stimulating hormone secretion** | GO:0046882 | **INHBA** | Inhibin beta A subunit | down | **-1.96** | 6.23E-03 |
| | | **INHBB** | Inhibin beta B subunit | down | **-1.68** | 4.33E-13 |
| **Regulation of endodermal cell fate specification** | GO:0042663 | **DUSP6** | Dual specificity phosphatase 6 | up | **2.85** | 1.83E-07 |
| | | **DKK1** | Dickkopf WNT signaling pathway inhibitor 1 | up | **2.47** | 3.24E-08 |
| **Tooth eruption** | GO:0044691 | **ADAMTS5** | ADAM metallopeptidase with thrombospondin type 1 motif 5 | down | **-2.24** | 3.58E-08 |
| | | **COL1A1** | Collagen type I alpha 1 chain | down | **-1.64** | 4.98E-03 |
| **Male gonad development** | GO:0008584 | **TESC** | Tescalcin | up | **1.69** | 7.70E-06 |
| | | **SOX8** | SRY-box 8 | down | **-1.97** | 1.41E-07 |

| | | | | | | |
|---|---|---|---|---|---|---|
| | | **INHBA** | Inhibin beta A subunit | down | **-1.96** | 6.23E-03 |
| | | **WNT2B** | Wnt family member 2B | down | **-1.74** | 2.06E-06 |
| | | **ASPM** | Abnormal spindle microtubule assembly | down | **-1.58** | 1.97E-06 |
| | | | | | | |
| **G2/M transition of mitotic cell cycle** | GO:0000086 | **PHLDA1** | Pleckstrin homology like domain family A member 1 | up | **1.88** | 1.08E-07 |
| | | **NEK2** | NIMA related kinase 2 | down | **-1.77** | 2.50E-03 |
| | | **PLK1** | Polo like kinase 1 | down | **-1.72** | 2.43E-04 |
| | | **FOXM1** | Forkhead box M1 | down | **-1.62** | 3.17E-03 |
| | | **CDK1** | Cyclin dependent kinase 1 | down | **-1.62** | 4.47E-10 |
| | | **TPX2** | TPX2, microtubule nucleation factor | down | **-1.60** | 1.84E-10 |
| | | | | | | |
| **Heart development** | GO:0007507 | **FGF12** | Fibroblast growth factor 12 | up | **2.03** | 3.17E-08 |
| | | **RPS6KA2** | Ribosomal protein S6 kinase A2 | up | **1.54** | 6.36E-26 |
| | | **SOD2** | Superoxide dismutase 2, mitochondrial | up | **1.52** | 2.06E-12 |
| | | **KCNAB1** | Potassium voltage-gated channel subfamily A member regulatory beta subunit 1 | down | **-2.41** | 3.67E-12 |
| | | **NTRK3** | Neurotrophic receptor tyrosine kinase 3 | down | **-1.97** | 1.20E-06 |
| | | **OXTR** | Oxytocin receptor | down | **-1.85** | 1.73E-15 |
| | | **COL3A1** | Collagen type III alpha 1 chain | down | **-1.52** | 1.44E-13 |
| | | | | | | |
| **Cell growth** | GO:0016049 | **IL7R** | Interleukin 7 receptor | up | **3.40** | 3.11E-08 |
| | | **NDRG4** | NDRG family member 4 | up | **2.71** | 1.93E-12 |
| | | **EFCAB13** | EF-hand calcium binding domain 13 | up | **1.94** | 2.60E-06 |
| | | **IL6** | Interleukin 6 | up | **1.76** | 3.15E-05 |
| | | | | | | |
| **Cellular response to hydrogen peroxide** | GO:0070301 | **ANXA1** | Annexin A1 | up | **1.93** | 1.17E-08 |
| | | **IL6** | Interleukin 6 | up | **1.76** | 3.15E-05 |
| | | **CYP1B1** | Cytochrome P450 family 1 subfamily B member 1 | up | **1.55** | 3.67E-05 |
| | | **CDK1** | Cyclin dependent kinase 1 | down | **-1.62** | 4.47E-10 |

| | | | | | | |
|---|---|---|---|---|---|---|
| **Regulation of cell shape** | GO:0008360 | **ANXA1** | Annexin A1 | up | **1.93** | 1.17E-08 |
| | | **IL6** | Interleukin 6 | up | **1.76** | 3.15E-05 |
| | | **FGD3** | FYVE, RhoGEF and PH domain containing 3 | down | **-2.16** | 4.81E-09 |
| | | **CDC42EP1** | CDC42 effector protein 1 | down | **-1.60** | 1.93E-31 |
| | | **CDC42EP2** | CDC42 effector protein 2 | down | **-1.55** | 3.78E-12 |
| | | **PLXNB3** | Plexin B3 | down | **-1.52** | 4.18E-11 |
| **Response to calcium ion** | GO:0051592 | **KCNMA1** | Potassium calcium-activated channel subfamily M alpha 1 | up | **1.92** | **6.93E-10** |
| | | **IL6** | Interleukin 6 | up | **1.76** | **3.15E-05** |
| | | **TNNT2** | Troponin T2, cardiac type | down | **-2.09** | **5.81E-11** |
| | | **AQP3** | Aquaporin 3 (Gill blood group) | down | **-1.54** | **1.97E-02** |
| **Mitotic sister chromatid segregation** | GO:0000070 | **NEK2** | NIMA related kinase 2 | down | **-1.77** | 2.50E-03 |
| | | **PLK1** | Polo like kinase 1 | down | **-1.72** | 2.43E-04 |
| | | **KIFC1** | Kinesin family member C1 | down | **-1.50** | 4.13E-05 |
| **Response to mechanical stimulus** | GO:0009612 | **FOSL1** | FOS like 1, AP-1 transcription factor subunit | up | **3.81** | 3.16E-08 |
| | | **KCNA5** | Potassium voltage-gated channel subfamily A member 5 | up | **1.53** | 3.15E-15 |
| | | **INHBB** | Inhibin beta B subunit | down | **-1.68** | 4.33E-13 |
| | | **COL3A1** | Collagen type III alpha 1 chain | down | **-1.52** | 1.44E-13 |

GO, gene ontology; up, up-regulated genes in the biological process gene ontology term; down, down-regulated genes in the biological process gene ontology term; DEG, differentially expressed gene; FC, fold-change.

**Supplementary Table S14.** Specific aldosterone-regulated enriched molecular function gene ontology terms as predicted by bioinformatic enrichment analysis.

**GO Molecular Function** analysis on DEGs

**Aldosterone $10^{-7}$ M *versus* Control**

| GO molecular function term | Link to GO ID | Gene name | Description | Regulation | FC | *P*-value |
|---|---|---|---|---|---|---|
| **Coreceptor activity** | GO:0015026 | **ITGB3** | Integrin subunit beta 3 | up | **1.71** | 3,11E-02 |
| | | **ITGA4** | Integrin subunit alpha 4 | up | **1.53** | 2,06E-03 |
| | | **CXCR4** | C-X-C motif chemokine receptor 4 | up | **1.52** | 2,33E-03 |
| | | **RAMP2** | Receptor activity modifying protein 2 | down | **-1.57** | 1,23E-02 |
| **Calcium ion binding** | GO:0005509 | **PCDH1** | Protocadherin 1 | up | **2.13** | 6,84E-04 |
| | | **S100A2** | S100 calcium binding protein A2 | up | **1.81** | 2,50E-04 |
| | | **SVEP1** | Sushi, von Willebrand factor type A, EGF and pentraxin domain containing 1 | up | **1.73** | 1,08E-02 |
| | | **S100A3** | S100 calcium binding protein A3 | up | **1.67** | 3,26E-03 |
| | | **MCTP2** | Multiple C2 and transmembrane domain containing 2 | up | **1.61** | 1,81E-06 |
| | | **S100A6** | S100 calcium binding protein A6 | up | **1.55** | 2,27E-03 |
| | | **DOC2A** | Double C2 domain alpha | down | **-2.31** | 3,39E-03 |
| | | **MGP** | Matrix Gla protein | down | **-2.16** | 5,66E-03 |
| | | **MYL3** | Myosin light chain 3 | down | **-1.78** | 3,41E-02 |
| | | **EFCAB5** | EF-hand calcium binding domain 5 | down | **-1.61** | 9,46E-03 |
| | | **KCNIP1** | Potassium voltage-gated channel interacting protein 1 | down | **-1.58** | 4,02E-04 |
| | | **AHRR** | Aryl-hydrocarbon receptor repressor | down | **-1.52** | 1,57E-02 |
| **Fibronectin binding** | GO:0001968 | **ITGB3** | Integrin subunit beta 3 | up | **1.71** | 3,11E-02 |
| | | **ITGA4** | Integrin subunit alpha 4 | up | **1.53** | 2,06E-03 |
| | | **IGFBP3** | Insulin like growth factor binding protein 3 | down | **-1.70** | 3,37E-05 |

| | | | | | | |
|---|---|---|---|---|---|---|
| **Receptor activity** | GO:0004872 | **PLAUR** | Plasminogen activator, urokinase receptor | up | **2.16** | 6,77E-03 |
| | | **ITGB3** | Integrin subunit beta 3 | up | **1.71** | 3,11E-02 |
| | | **ITGAX** | Integrin subunit alpha X | up | **1.51** | 1,27E-02 |
| | | **RTN4RL2** | Reticulon 4 receptor-like 2 | down | **-1.67** | 3,22E-04 |
| | | **GFRA2** | GDNF family receptor alpha 2 | down | **-1.61** | 6,54E-04 |
| | | **CADM3** | Cell adhesion molecule 3 | down | **-1.59** | 1,70E-03 |
| **Transcriptional activator activity, RNA polymerase II core promoter proximal region sequence-specific binding** | GO:0001077 | **EHF** | ETS homologous factor | up | **3.55** | 2,68E-04 |
| | | **FOSL1** | FOS like 1, AP-1 transcription factor subunit | up | **1.89** | 3,69E-03 |
| | | **SIX1** | SIX homeobox 1 | down | **-1.95** | 4,58E-03 |
| | | **EGR1** | Early growth response 1 | down | **-1.85** | 3,76E-04 |
| | | **MAFA** | MAF bZIP transcription factor A | down | **-1.80** | 7,75E-04 |
| | | **EGR2** | Early growth response 2 | down | **-1.69** | 4,72E-02 |
| **Transcription factor activity, sequence-specific DNA binding** | GO:0003700 | **EHF** | ETS homologous factor | up | **3.55** | 2,68E-04 |
| | | **ZBTB16** | Zinc finger and BTB domain containing 16 | up | **3.35** | 2,87E-06 |
| | | **POU5F1** | POU class 5 homeobox 1 | up | **2.97** | 1,80E-05 |
| | | **FOSL1** | FOS like 1, AP-1 transcription factor subunit | up | **1.89** | 3,69E-03 |
| | | **RXRG** | Retinoid X receptor gamma | up | **1.71** | 2,61E-03 |
| | | **HMGA1** | High mobility group AT-hook 1 | up | **1.53** | 7,70E-03 |
| | | **SIX1** | SIX homeobox 1 | down | **-1.95** | 4,58E-03 |
| | | **EGR1** | Early growth response 1 | down | **-1.85** | 3,76E-04 |
| | | **SOX3** | SRY-box 3 | down | **-1.80** | 3,61E-03 |
| | | **MAFA** | MAF bZIP transcription factor A | down | **-1.80** | 7,75E-04 |
| | | **EGR2** | Early growth response 2 | down | **-1.69** | 4,72E-02 |
| | | **TBX6** | T-box 6 | down | **-1.56** | 4,27E-02 |
| | | **ZNF837** | Zinc finger protein 837 | down | **-1.51** | 6,00E-04 |
| **C-X3-C chemokine binding** | GO:0019960 | **ITGB3** | Integrin subunit beta 3 | up | **1.71** | 3,11E-02 |
| | | **ITGA4** | integrin subunit alpha 4 | up | **1.53** | 2,06E-03 |

| | | | | | | |
|---|---|---|---|---|---|---|
| **Receptor binding** | GO:0005102 | **PLAUR** | Plasminogen activator, urokinase receptor | up | **2.16** | 6,77E-03 |
| | | **AGXT** | Alanine-glyoxylate aminotransferase | up | **2.13** | 4,53E-02 |
| | | **TNFSF18** | Tumor necrosis factor superfamily member 18 | up | **2.00** | 2,57E-02 |
| | | **TAC4** | Tachykinin 4 (hemokinin) | up | **2.00** | 3,64E-02 |
| | | **PLAT** | Plasminogen activator, tissue type | up | **1.60** | 1,38E-02 |
| | | **PCSK1N** | Proprotein convertase subtilisin/kexin type 1 inhibitor | down | **-1.86** | 1,03E-03 |
| | | **CADM3** | Cell adhesion molecule 3 | down | **-1.59** | 1,70E-03 |
| | | | | | | |
| **RNA polymerase II core promoter proximal region sequence-specific DNA binding** | GO:0000978 | **EHF** | ETS homologous factor | up | **3.55** | 2,68E-04 |
| | | **FOSL1** | FOS like 1, AP-1 transcription factor subunit | up | **1.89** | 3,69E-03 |
| | | **ZNF114** | Zinc finger protein 114 | up | **1.57** | 1,64E-02 |
| | | **PER1** | Period circadian clock 1 | up | **1.51** | 3,53E-06 |
| | | **SIX1** | SIX homeobox 1 | down | **-1.95** | 4,58E-03 |
| | | **MAFA** | MAF bZIP transcription factor A | down | **-1.80** | 7,75E-04 |
| | | **EGR2** | Early growth response 2 | down | **-1.69** | 4,72E-02 |
| | | | | | | |
| **Ubiquitin protein ligase binding** | GO:0031625 | **POU5F1** | POU class 5 homeobox 1 | up | **2.97** | 1,80E-05 |
| | | **LTBR** | Lymphotoxin beta receptor | up | **2.71** | 2,30E-04 |
| | | **GPR75** | G protein-coupled receptor 75 | up | **1.58** | 7,16E-03 |
| | | **CXCR4** | C-X-C motif chemokine receptor 4 | up | **1.52** | 2,33E-03 |
| | | **PER1** | Period circadian clock 1 | up | **1.51** | 3,53E-06 |
| | | **EGR2** | Early growth response 2 | down | **-1.69** | 4,72E-02 |
| | | | | | | |
| **Protein kinase inhibitor activity** | GO:0004860 | **RTN4RL2** | Reticulon 4 receptor-like 2 | down | **-1.67** | 3,22E-04 |
| | | **BGN** | Biglycan | down | **-1.57** | 3,68E-03 |
| | | **SOCS1** | Suppressor of cytokine signaling 1 | down | **-1.51** | 3,26E-02 |

GO, gene ontology; up, up-regulated genes in the molecular function gene ontology term; down, down-regulated genes in the molecular function gene ontology term; DEG, differentially expressed gene; FC, fold-change.

**Supplemental Table S15.** Specific cortisol-regulated enriched molecular function gene ontology terms as predicted by bioinformatic enrichment analysis.

**GO Molecular Function** analysis on DEGs

**Cortisol $10^{-7}$ M *versus* Control**

| GO molecular function term | Link to GO ID | Gene name | Description | Regulation | FC | *P*-value |
|---|---|---|---|---|---|---|
| **Protein serine/threonine/tyrosine kinase activity** | GO:0004712 | **RPS6KA2** | Ribosomal protein S6 kinase A2 | up | **1.79** | 4.35E-45 |
| | | **SGK1** | Serum/glucocorticoid regulated kinase 1 | up | **1.57** | 8.43E-35 |
| | | **TTK** | TTK protein kinase | down | **-1.54** | 3.29E-03 |
| **Thyroid hormone transmembrane transporter activity** | GO:0015349 | **SLC16A10** | Solute carrier family 16 member 10 | up | **1.57** | 1.76E-30 |
| | | **SLCO4A1** | Solute carrier organic anion transporter family member 4A1 | up | **1.53** | 9.95E-16 |
| **Metalloendopeptidase activity** | GO:0004222 | **PAPLN** | Papilin, proteoglycan like sulfated glycoprotein | up | **1.75** | 4.18E-05 |
| | | **ADAMTSL4** | ADAMTS like 4 | up | **1.53** | 4.94E-18 |
| | | **PAPPA** | Pappalysin 1 | down | **-1.66** | 1.58E-02 |
| | | **ADAMTS1** | ADAM metallopeptidase with thrombospondin type 1 motif 1 | down | **-1.57** | 8.90E-12 |
| **GPI-linked ephrin receptor activity** | GO:0005004 | **EPHA8** | EPH receptor A8 | up | **2.38** | 4.21E-20 |
| | | **NTRK3** | Neurotrophic receptor tyrosine kinase 3 | down | **-1.61** | 4.88E-04 |

GO, gene ontology; up, up-regulated genes in the molecular function gene ontology term; down, down-regulated genes in the molecular function gene ontology term; DEG, differentially expressed gene; FC, fold-change.

**Supplemental Table S16.** Specific cortisol + RU-486-regulated enriched molecular function gene ontology terms as predicted by bioinformatic enrichment analysis.

**GO Molecular Function** analysis on DEGs

**Cortisol $10^{-7}$ M + RU-486 $10^{-5}$ M *versus* Control**

| GO molecular function term | Link to GO ID | Gene name | Description | Regulation | FC | *P*-value |
|---|---|---|---|---|---|---|
| **Carbonate dehydratase activity** | GO:0004089 | **CA12** | Carbonic anhydrase 12 | up | **2.28** | 1.56E-06 |
| | | **CA2** | Carbonic anhydrase 2 | down | **-1.55** | 2.48E-06 |
| | | **CA11** | Carbonic anhydrase 11 | down | **-1.51** | 8.23E-15 |
| | | **CA9** | Carbonic anhydrase 9 | down | **-1.50** | 7.90E-24 |
| **Anaphase-promoting complex binding** | GO:0010997 | **CDC20B** | Cell division cycle 20B | down | **-1.85** | 3.55E-03 |
| | | **PLK1** | Polo like kinase 1 | down | **-1.72** | 2.43E-04 |
| | | **CDC20** | Cell division cycle 20 | down | **-1.52** | 1.12E-03 |
| **Extracellular matrix binding** | GO:0050840 | **EFCAB13** | EF-hand calcium binding domain 13 | up | **1.94** | 2.60E-06 |
| | | **SPARCL1** | SPARC like 1 | up | **1.53** | 2.85E-08 |
| | | **ADAMTS5** | ADAM metallopeptidase with thrombospondin type 1 motif 5 | down | **-2.24** | 3.58E-08 |
| | | **ADAMTS15** | ADAM metallopeptidase with thrombospondin type 1 motif 15 | down | **-1.67** | 1.10E-04 |
| **Collagen binding** | GO:0005518 | **C1QTNF1** | C1q and tumor necrosis factor related protein 1 | up | **1.56** | 9.15E-14 |
| | | **CD44** | CD44 molecule (Indian blood group) | up | **1.54** | 2.73E-09 |
| | | **SPARCL1** | SPARC like 1 | up | **1.53** | 2.85E-08 |
| | | **ITGA11** | Integrin subunit alpha 11 | down | **-2.19** | 7.41E-07 |
| | | **LUM** | Lumican | down | **-1.72** | 6.80E-05 |

| | | | | | | |
|---|---|---|---|---|---|---|
| **Extracellular matrix structural constituent** | GO:0005201 | **MGP** | Matrix Gla protein | down | **-2.21** | 5.47E-07 |
| | | **HAPLN3** | Hyaluronan and proteoglycan link protein 3 | down | **-2.00** | 8.82E-05 |
| | | **LUM** | Lumican | down | **-1.72** | 6.80E-05 |
| | | **COL1A1** | Collagen type I alpha 1 chain | down | **-1.64** | 4.98E-03 |
| | | **COL3A1** | Collagen type III alpha 1 chain | down | **-1.52** | 1.44E-13 |
| | | | | | | |
| **Aldo-keto reductase (NADP) activity** | GO:0004033 | **AKR1C1** | Aldo-keto reductase family 1 member C1 | up | **16.20** | 8.08E-17 |
| | | **AKR1B10** | Aldo-keto reductase family 1 member B10 | up | **7.56** | 1.07E-12 |
| | | **KCNAB1** | Potassium voltage-gated channel subfamily A member regulatory beta subunit 1 | down | **-2.41** | 3.67E-12 |
| | | | | | | |
| **Symporter activity** | GO:0015293 | **SLC24A4** | Solute carrier family 24 member 4 | up | **2.12** | 1.61E-05 |
| | | **SLC1A7** | Solute carrier family 1 member 7 | up | **1.84** | 6.79E-11 |
| | | **SLC16A6** | Solute carrier family 16 member 6 | up | **1.53** | 4.76E-05 |
| | | **MFSD2A** | Major facilitator superfamily domain containing 2A | down | **-1.69** | 2.54E-11 |
| | | | | | | |
| **Protein kinase binding** | GO:0019901 | **SPRY2** | Sprouty RTK signaling antagonist 2 | up | **1.77** | 3.61E-07 |
| | | **TRIB3** | Tribbles pseudokinase 3 | up | **1.71** | 2.94E-06 |
| | | **KCNA5** | Potassium voltage-gated channel subfamily A member 5 | up | **1.53** | 3.15E-15 |
| | | **ACTA2** | Actin, alpha 2, smooth muscle, aorta | down | **-2.13** | 4.92E-19 |
| | | **KIF20A** | Kinesin family member 20A | down | **-2.04** | 5.94E-08 |
| | | **CCNA2** | Cyclin A2 | down | **-1.72** | 1.60E-07 |
| | | **PLK1** | Polo like kinase 1 | down | **-1.72** | 2.43E-04 |
| | | **FOXM1** | Forkhead box M1 | down | **-1.62** | 3.17E-03 |
| | | **TPX2** | TPX2, microtubule nucleation factor | down | **-1.60** | 1.84E-10 |
| | | **CDKN2C** | Cyclin dependent kinase inhibitor 2C | down | **-1.57** | 1.97E-04 |
| | | **CCND2** | Cyclin D2 | down | **-1.54** | 2.85E-17 |
| | | | | | | |
| **ATP binding** | GO:0005524 | **HSPA6** | Heat shock protein family A member 6 | up | **13.96** | 9.08E-12 |
| | | **ABCC3** | ATP binding cassette subfamily C member 3 | up | **2.60** | 6.14E-08 |

| | | | | | | |
|---|---|---|---|---|---|---|
| | | **PDK4** | Pyruvate dehydrogenase kinase 4 | up | **2.21** | 1.14E-10 |
| | | **ACSL5** | Acyl-CoA synthetase long-chain family member 5 | up | **1.74** | 6.15E-04 |
| | | **TRIB3** | Tribbles pseudokinase 3 | up | **1.71** | 2.94E-06 |
| | | **RPS6KA2** | Ribosomal protein S6 kinase A2 | up | **1.54** | 6.36E-26 |
| | | **ABCA4** | ATP binding cassette subfamily A member 4 | up | **1.54** | 5.36E-08 |
| | | **EPHA8** | EPH receptor A8 | up | **1.50** | 2.43E-05 |
| | | **TGM2** | Transglutaminase 2 | down | **-2.86** | 6.47E-06 |
| | | **ACTA2** | Actin, alpha 2, smooth muscle, aorta | down | **-2.13** | 4.92E-19 |
| | | **KIF20A** | Kinesin family member 20A | down | **-2.04** | 5.94E-08 |
| | | **CARNS1** | Carnosine synthase 1 | down | **-2.02** | 5.05E-16 |
| | | **NTRK3** | Neurotrophic receptor tyrosine kinase 3 | down | **-1.97** | 1.20E-06 |
| | | **FGFR3** | Fibroblast growth factor receptor 3 | down | **-1.91** | 5.84E-19 |
| | | **ATP6V1B1** | ATPase $H^+$ transporting V1 subunit B1 | down | **-1.85** | 5.46E-07 |
| | | **BUB1B** | BUB1 mitotic checkpoint serine/threonine kinase B | down | **-1.81** | 2.76E-11 |
| | | **NEK2** | NIMA related kinase 2 | down | **-1.77** | 2.50E-03 |
| | | **PLK1** | Polo like kinase 1 | down | **-1.72** | 2.43E-04 |
| | | **KIF4A** | Kinesin family member 4A | down | **-1.71** | 6.57E-05 |
| | | **MKI67** | Marker of proliferation Ki-67 | down | **-1.69** | 6.36E-05 |
| | | **CDK1** | Cyclin dependent kinase 1 | down | **-1.62** | 4.47E-10 |
| | | **UBE2T** | Ubiquitin conjugating enzyme E2 T | down | **-1.61** | 1.75E-04 |
| | | **TPX2** | TPX2, microtubule nucleation factor | down | **-1.60** | 1.84E-10 |
| | | **ABCB5** | ATP binding cassette subfamily B member 5 | down | **-1.59** | 3.70E-08 |
| | | **ENTPD8** | Ectonucleoside triphosphate diphosphohydrolase 8 | down | **-1.57** | 4.41E-10 |
| | | **TOP2A** | Topoisomerase (DNA) II alpha | down | **-1.56** | 2.82E-06 |
| | | **PLXNB3** | Plexin B3 | down | **-1.52** | 4.18E-11 |
| | | **ABCG1** | ATP binding cassette subfamily G member 1 | down | **-1.52** | 5.50E-18 |
| | | **KIFC1** | Kinesin family member C1 | down | **-1.50** | 4.13E-05 |
| **Hyaluronic acid binding** | GO:0005540 | **LAYN** | Layilin | up | **1.55** | 4.50E-10 |
| | | **CD44** | CD44 molecule (Indian blood group) | up | **1.54** | 2.73E-09 |
| | | **HAPLN3** | Hyaluronan and proteoglycan link protein 3 | down | **-2.00** | 8.82E-05 |

| | | | | | | |
|---|---|---|---|---|---|---|
| **Indanol dehydrogenase activity** | GO:0047718 | **AKR1C1** | Aldo-keto reductase family 1 member C1 | up | **16.20** | 8.08E-17 |
| | | **AKR1B10** | Aldo-keto reductase family 1 member B10 | up | **7.56** | 1.07E-12 |
| **Oxidoreductase activity, acting on paired donors, with incorporation or reduction of molecular oxygen** | GO:0016705 | **CYP1B1** | Cytochrome P450 family 1 subfamily B member 1 | up | **1.55** | 3.67E-05 |
| | | **CYP2W1** | Cytochrome P450 family 2 subfamily W member 1 | down | **-1.94** | 1.98E-11 |
| | | **CYP19A1** | Cytochrome P450 family 19 subfamily A member 1 | down | **-1.89** | 5.27E-06 |
| | | **CYP2S1** | Cytochrome P450 family 2 subfamily S member 1 | down | **-1.67** | 1.31E-04 |
| **Peptide binding** | GO:0042277 | **MME** | Membrane metallo-endopeptidase | up | **1.79** | 8.51E-07 |
| | | **TMEM158** | Transmembrane protein 158 | up | **1.63** | 3.83E-20 |
| | | **GPR37L1** | G protein-coupled receptor 37 like 1 | down | **-2.01** | 2.81E-06 |
| | | **OXTR** | Oxytocin receptor | down | **-1.85** | 1.73E-15 |
| **Steroid hydroxylase activity** | GO:0008395 | **CYP2W1** | Cytochrome P450 family 2 subfamily W member 1 | down | **-1.94** | 1.98E-11 |
| | | **CYP19A1** | Cytochrome P450 family 19 subfamily A member 1 | down | **-1.89** | 5.27E-06 |
| | | **CYP2S1** | Cytochrome P450 family 2 subfamily S member 1 | down | **-1.67** | 1.31E-04 |

GO, gene ontology; up, up-regulated genes in the molecular function gene ontology term; down, down-regulated genes in the molecular function gene ontology term; DEG, differentially expressed gene; FC, fold-change.

**Supplemental Table S17.** Specific aldosterone-regulated enriched cellular component gene ontology terms as predicted by bioinformatic enrichment analysis.

**GO Cellular Component** analysis on DEGs

**Aldosterone $10^{-7}$ M *versus* Control**

| GO cellular components term | Link to GO ID | Gene name | Description | Regulation | FC | *P*-value |
|---|---|---|---|---|---|---|
| **Cell surface** | GO:0009986 | **OPRD1** | Opioid receptor delta 1 | up | **1.55** | 2,95E-03 |
| | | **DOC2A** | Double C2 domain alpha | down | **-2.31** | 3,39E-03 |
| | | **PLP1** | Proteolipid protein 1 | down | **-1.87** | 3,19E-02 |
| | | **SST** | Somatostatin | down | **-1.81** | 1,02E-04 |
| | | **GJD2** | Gap junction protein delta 2 | down | **-1.67** | 1,70E-02 |
| | | **TPGS1** | Tubulin polyglutamylase complex subunit 1 | down | **-1.59** | 1,70E-03 |
| | | **KCNMB2** | Potassium calcium-activated channel subfamily M regulatory beta subunit 2 | down | **-1.56** | 1,09E-02 |
| | | **SLC12A5** | Solute carrier family 12 member 5 | down | **-1.52** | 2,40E-03 |
| | | **HTR1D** | 5-hydroxytryptamine receptor 1D | down | **-1.51** | 4,09E-02 |
| **Integral component of plasma membrane** | GO:0005887 | **LTBR** | Lymphotoxin beta receptor | up | **2.71** | 2,30E-04 |
| | | **SCNN1A** | Sodium channel epithelial 1 alpha subunit | up | **2.22** | 1,02E-06 |
| | | **PLAUR** | Plasminogen activator, urokinase receptor | up | **2.16** | 6,77E-03 |
| | | **PCDH1** | Protocadherin 1 | up | **2.13** | 6,84E-04 |
| | | **GRID2** | Glutamate ionotropic receptor delta type subunit 2 | up | **2.02** | 1,15E-02 |
| | | **HVCN1** | Hydrogen voltage gated channel 1 | up | **1.87** | 4,26E-03 |
| | | **SLC3A1** | Solute carrier family 3 member 1 | up | **1.87** | 4,91E-04 |
| | | **P2RY6** | Pyrimidinergic receptor P2Y6 | up | **1.71** | 4,23E-02 |
| | | **ITGB3** | EF-hand calcium binding domain 13 // integrin subunit beta 3 | up | **1.71** | 3,11E-02 |
| | | **EPHA8** | EPH receptor A8 | up | **1.70** | 2,77E-02 |
| | | **GPR75** | G protein-coupled receptor 75 | up | **1.58** | 7,16E-03 |
| | | **PTGER2** | Prostaglandin E receptor 2 | up | **1.57** | 7,18E-06 |
| | | **ABCC3** | ATP binding cassette subfamily C member 3 | up | **1.55** | 2,40E-02 |
| | | **OPRD1** | Opioid receptor delta 1 | up | **1.55** | 2,95E-03 |

| | | | | | | |
|---|---|---|---|---|---|---|
| | | **PLPPR3** | Phospholipid phosphatase related 3 | down | **-1.64** | 1,00E-03 |
| | | **PTPRC** | Protein tyrosine phosphatase, receptor type C | down | **-1.64** | 2,25E-02 |
| | | **CADM3** | Cell adhesion molecule 3 | down | **-1.59** | 1,70E-03 |
| | | **RAMP2** | Receptor activity modifying protein 2 | down | **-1.57** | 1,23E-02 |
| | | **KCNMB2** | Potassium calcium-activated channel subfamily M regulatory beta subunit 2 | down | **-1.56** | 1,09E-02 |
| | | **SLC12A5** | Solute carrier family 12 member 5 | down | **-1.52** | 2,40E-03 |
| | | **HTR1D** | 5-hydroxytryptamine receptor 1D | down | **-1.51** | 4,09E-02 |
| | | | | | | |
| | | **ITGB3** | Integrin subunit beta 3 | up | **1.71** | 3,11E-02 |
| **Integrin complex** | GO:0008305 | **ITGA4** | Integrin subunit alpha 4 | up | **1.53** | 2,06E-03 |
| | | **ITGAX** | Integrin subunit alpha X | up | **1.51** | 1,27E-02 |
| | | | | | | |
| | | **PLAUR** | Plasminogen activator, urokinase receptor | up | **2.16** | 6,77E-03 |
| | | **SPRY4** | Sprouty RTK signaling antagonist 4 | up | **1.82** | 4,71E-03 |
| | | **ITGB3** | Integrin subunit beta 3 | up | **1.71** | 3,11E-02 |
| | | **ITGA4** | Integrin subunit alpha 4 | up | **1.53** | 2,06E-03 |
| **Focal adhesion** | GO:0005925 | **HMGA1** | High mobility group AT-hook 1 | up | **1.53** | 7,70E-03 |
| | | **CNN1** | Calponin 1 | down | **-1.72** | 1,02E-02 |
| | | **RPL13AP5** | Ribosomal protein L13a pseudogene 5 | down | **-1.72** | 2,24E-04 |
| | | **PTPRC** | Protein tyrosine phosphatase, receptor type C | down | **-1.64** | 2,25E-02 |
| | | | | | | |
| | | **SERPINA3** | Serpin family A member 3 | up | **2.99** | 1,86E-03 |
| | | **TNFSF18** | Tumor necrosis factor superfamily member 18 | up | **2.00** | 2,57E-02 |
| | | **TAC4** | Tachykinin 4 (hemokinin) | up | **2.00** | 3,64E-02 |
| | | **OMD** | Osteomodulin | up | **1.87** | 2,87E-03 |
| | GO:0005615 | **PLAT** | Plasminogen activator, tissue type | up | **1.60** | 1,38E-02 |
| **Extracellular space** | | **DSCAML1** | DS cell adhesion molecule like 1 | up | **1.57** | 7,08E-03 |
| | | **PTX3** | Pentraxin 3 | up | **1.54** | 2,66E-02 |
| | | **HIST1H2BC** | Histone cluster 1, H2bc | up | **1.54** | 7,46E-03 |
| | | **FMOD** | Fibromodulin | down | **-2.09** | 3,28E-02 |
| | | **GDF7** | Growth differentiation factor 7 | down | **-2.01** | 2,21E-03 |

| | | | | | |
|---|---|---|---|---|---|
| | **PCSK1N** | Proprotein convertase subtilisin/kexin type 1 inhibitor | down | **-1.86** | 1,03E-03 |
| | **SST** | Somatostatin | down | **-1.81** | 1,02E-04 |
| | **ACTG2** | Actin, gamma 2, smooth muscle, enteric | down | **-1.73** | 3,72E-02 |
| | **IGFBP3** | Insulin like growth factor binding protein 3 | down | **-1.70** | 3,37E-05 |
| | **CLEC11A** | C-type lectin domain family 11 member A | down | **-1.54** | 4,83E-03 |
| | **COL2A1** | Collagen type II alpha 1 chain | down | **-1.52** | 2,59E-03 |
| | **CFD** | Complement factor D | down | **-1.52** | 4,62E-03 |

GO, gene ontology; up, up-regulated genes in the cellular component gene ontology term; down, down-regulated genes in the cellular component gene ontology term; DEG, differentially expressed gene; FC, fold-change.

**Supplemental Table S18.** Specific cortisol-regulated enriched cellular component gene ontology terms as predicted by bioinformatic enrichment analysis.

**GO Cellular Component** analysis on DEGs

**Cortisol $10^{-7}$ M *versus* Control**

| GO cellular components term | Link to GO ID | Gene name | Description | Regulation | FC | *P*-value |
|---|---|---|---|---|---|---|
| **Integral component of plasma membrane** | GO:0005887 | **SCNN1A** | Sodium channel epithelial 1 alpha subunit | up | **6.48** | 1.80E-152 |
| | | **HVCN1** | Hydrogen voltage gated channel 1 | up | **4.15** | 4.71E-32 |
| | | **SLC28A3** | Solute carrier family 28 member 3 | up | **2.66** | 2.79E-12 |
| | | **PTGER2** | Prostaglandin E receptor 2 | up | **2.62** | 5.28E-54 |
| | | **EPHA8** | EPH receptor A8 | up | **2.38** | 4.21E-20 |
| | | **SLC13A4** | Solute carrier family 13 member 4 | up | **2.03** | 3.40E-95 |
| | | **SLC16A12** | Solute carrier family 16 member 12 | up | **1.96** | 6.25E-39 |
| | | **ABCC5** | ATP binding cassette subfamily C member 5 | up | **1.75** | 3.28E-22 |
| | | **ST3GAL5** | ST3 beta-galactoside alpha-2,3-sialyltransferase 5 | up | **1.72** | 1.01E-12 |
| | | **ABCA4** | ATP binding cassette subfamily A member 4 | up | **1.69** | 4.72E-11 |
| | | **TGFBR2** | Transforming growth factor beta receptor 2 | up | **1.64** | 2.09E-18 |
| | | **SLC2A12** | Solute carrier family 2 member 12 | up | **1.58** | 2.58E-42 |
| | | **KCNA5** | Potassium voltage-gated channel subfamily A member 5 | up | **1.57** | 6.84E-17 |
| | | **SLC16A10** | Solute carrier family 16 member 10 | up | **1.57** | 1.76E-30 |
| | | **SLCO4A1** | Solute carrier organic anion transporter family member 4A1 | up | **1.53** | 9.95E-16 |
| | | **CD58** | CD58 molecule | up | **1.51** | 1.09E-05 |
| | | **KCND2** | Potassium voltage-gated channel subfamily D member 2 | down | **-1.74** | 8.81E-05 |
| | | **NTRK3** | Neurotrophic receptor tyrosine kinase 3 | down | **-1.61** | 4.88E-04 |
| | | | | | | |
| **Insulin-like growth factor binding protein complex** | GO:0016942 | **IGF1** | Insulin like growth factor 1 | down | **-1.85** | 1.51E-18 |
| | | **IGFBP3** | Insulin like growth factor binding protein 3 | down | **-1.64** | 1.77E-03 |
| | | | | | | |
| | GO:0042567 | **IGF1** | Insulin like growth fact | down | **-1.85** | 1.51E-18 |

| | | | | | | |
|---|---|---|---|---|---|---|
| **Insulin-like growth factor ternary complex** | | **IGFBP3** | Insulin like growth fact | down | **-1.64** | 1.77E-03 |
| | | | | | | |
| **Proteinaceous extracellular matrix** | GO:0005578 | **PAPLN** | Papilin, proteoglycan like sulfated glycoprotein | up | **1.75** | 4.18E-05 |
| | | **WNT11** | Wnt family member 11 | up | **1.61** | 3.74E-04 |
| | | **ADAMTSL1** | ADAMTS like 1 | up | **1.53** | 3.58E-05 |
| | | **AMTN** | Amelotin | down | **-2.08** | 1.63E-06 |
| | | **SMOC1** | SPARC related modular calcium binding 1 | down | **-1.63** | 1.12E-04 |
| | | **ADAMTS1** | ADAM metallopeptidase with thrombospondin type 1 motif 1 | down | **-1.57** | 8.90E-12 |
| | | | | | | |
| **Extracellular space** | GO:0005615 | **SERPINA3** | Serpin family A member 3 | up | **2.53** | 1.29E-22 |
| | | **CES4A** | Carboxylesterase 4A | up | **2.06** | 7.08E-08 |
| | | **TLE2** | Transducin like enhancer of split 2 | up | **2.31** | 2.98E-22 |
| | | **DKK1** | Dickkopf WNT signaling pathway inhibitor 1 | up | **1.76** | 5.76E-04 |
| | | **CP** | Ceruloplasmin | up | **1.70** | 8.53E-12 |
| | | **SERPINF2** | Serpin family F member 2 | up | **1.70** | 2.01E-40 |
| | | **SCGB1D2** | Secretoglobin family 1D member 2 | up | **1.68** | 2.03E-04 |
| | | **WNT11** | Wnt family member 11 | up | **1.61** | 3.74E-04 |
| | | **KRT81** | Keratin 81 | down | **-2.35** | 9.24E-04 |
| | | **SPP1** | Secreted phosphoprotein 1 | down | **-2.25** | 5.38E-05 |
| | | **IGF1** | Insulin like growth factor 1 | down | **-1.85** | 1.51E-18 |
| | | **SFRP2** | Secreted frizzled related protein 2 | down | **-1.78** | 2.39E-03 |
| | | **SST** | Somatostatin | down | **-1.69** | 3.85E-03 |
| | | **COL1A1** | Collagen type I alpha 1 chain | down | **-1.68** | 3.34E-03 |
| | | **PAPPA** | Pappalysin 1 | down | **-1.66** | 1.58E-02 |
| | | **IGFBP3** | Insulin like growth factor binding protein 3 | down | **-1.64** | 1.77E-03 |

GO, gene ontology; up, up-regulated genes in the cellular component gene ontology term; down, down-regulated genes in the cellular component gene ontology term; DEG, differentially expressed gene; FC, fold-change.

**Supplemental Table S19.** Specific cortisol + RU-486-regulated enriched cellular component gene ontology terms as predicted by bioinformatic enrichment analysis.

**GO Cellular Component** analysis on DEGs

**Cortisol $10^{-7}$ M + RU-486 $10^{-5}$ M *versus* Control**

| GO cellular component term | Link to GO ID | Gene name | Description | Regulation | FC | *P*-value |
|---|---|---|---|---|---|---|
| **Extracellular matrix** | GO:0031012 | **MMP1** | Matrix metallopeptidase 1 | up | **6.06** | 2.25E-08 |
| | | **SERPINE2** | Serpin family E member 2 | up | **2.12** | 3.52E-09 |
| | | **ECM1** | Extracellular matrix protein 1 | up | **1.82** | 3.16E-06 |
| | | **SERPINE1** | Serpin family E member 1 | up | **1.54** | 2.46E-03 |
| | | **TGM2** | Transglutaminase 2 | down | **-2.86** | 6.47E-06 |
| | | **MGP** | Matrix Gla protein | down | **-2.21** | 5.47E-07 |
| | | **MMP11** | Matrix metallopeptidase 11 | down | **-1.76** | 9.27E-23 |
| | | **LUM** | Lumican | down | **-1.72** | 6.80E-05 |
| | | **COL1A1** | Collagen type I alpha 1 chain | down | **-1.64** | 4.98E-03 |
| | | **MFAP4** | Microfibrillar associated protein 4 | down | **-1.55** | 2.66E-07 |
| | | **THBS2** | Thrombospondin 2 | down | **-1.53** | 2.41E-06 |
| | | **MFGE8** | Milk fat globule-EGF factor 8 protein | down | **-1.52** | 9.70E-30 |
| | | **COL3A1** | Collagen type III alpha 1 chain | down | **-1.52** | 1.44E-13 |
| | | | | | | |
| **Plasma membrane** | GO:0005886 | **IL7R** | Interleukin 7 receptor | up | **3.40** | 3.11E-08 |
| | | **PLIN2** | Perilipin 2 | up | **2.83** | 3.52E-64 |
| | | **ABCC3** | ATP binding cassette subfamily C member 3 | up | **2.60** | 6.14E-08 |
| | | **DKK1** | Dickkopf WNT signaling pathway inhibitor 1 | up | **2.47** | 3.24E-08 |
| | | **B4GALNT1** | Beta-1,4-N-acetyl-galactosaminyltransferase 1 | up | **2.46** | 3.94E-08 |
| | | **PLAUR** | Plasminogen activator, urokinase receptor | up | **2.38** | 1.14E-10 |
| | | **CA12** | Carbonic anhydrase 12 | up | **2.28** | 1.56E-06 |
| | | **SCNN1A** | Sodium channel epithelial 1 alpha subunit | up | **2.21** | 3.92E-26 |
| | | **CLMP** | CXADR like membrane protein | up | **2.17** | 1.94E-07 |
| | | **SLC24A4** | Solute carrier family 24 member 4 | up | **2.12** | 1.61E-05 |
| | | **HVCN1** | Hydrogen voltage gated channel 1 | up | **1.97** | 7.60E-08 |
| | | **PTGER2** | Prostaglandin E receptor 2 | up | **1.95** | 1.63E-26 |

| | | | | |
|---|---|---|---|---|
| **EFCAB13** | EF-hand calcium binding domain 13 | up | **1.94** | 2.60E-06 |
| **ANXA1** | Annexin A1 | up | **1.93** | 1.17E-08 |
| **KCNMA1** | Potassium calcium-activated channel subfamily M alpha 1 | up | **1.92** | 6.93E-10 |
| **CDCP1** | CUB domain containing protein 1 | up | **1.84** | 3.65E-12 |
| **SLC1A7** | Solute carrier family 1 member 7 | up | **1.84** | 6.79E-11 |
| **MME** | Membrane metallo-endopeptidase | up | **1.79** | 8.51E-07 |
| **SPRY2** | Sprouty RTK signaling antagonist 2 | up | **1.77** | 3.61E-07 |
| **PTPRN** | Protein tyrosine phosphatase, receptor type N | up | **1.73** | 2.71E-05 |
| **SLC3A1** | Solute carrier family 3 member 1 | up | **1.72** | 1.76E-03 |
| **TRIB3** | Tribbles pseudokinase 3 | up | **1.71** | 2.94E-06 |
| **TESC** | Tescalcin | up | **1.69** | 7.70E-06 |
| **PRLR** | Prolactin receptor | up | **1.66** | 1.57E-18 |
| **ANO4** | Anoctamin 4 | up | **1.65** | 1.28E-03 |
| **CXCR4** | C-X-C motif chemokine receptor 4 | up | **1.64** | 1.05E-10 |
| **SLC22A6** | Solute carrier family 22 member 6 | up | **1.63** | 1.17E-05 |
| **SLC9A9** | Solute carrier family 9 member A9 | up | **1.61** | 3.20E-07 |
| **RAET1G** | Retinoic acid early transcript 1G | up | **1.60** | 1.87E-02 |
| **TNFSF18** | Tumor necrosis factor superfamily member 18 | up | **1.59** | 8.30E-04 |
| **IFITM1** | Interferon induced transmembrane protein 1 | up | **1.59** | 2.00E-05 |
| **ATP1B2** | ATPase $Na^+/K^+$ transporting subunit beta 2 | up | **1.57** | 1.62E-10 |
| **SLC18A3** | Solute carrier family 18 member A3 | up | **1.55** | 1.33E-26 |
| **ETV5** | ETS variant 5 | up | **1.55** | 4.11E-10 |
| **GNG2** | G protein subunit gamma 2 | up | **1.54** | 5.57E-13 |
| **SERPINE1** | Serpin family E member 1 | up | **1.54** | 2.46E-03 |
| **CD44** | CD44 molecule (Indian blood group) | up | **1.54** | 2.73E-09 |
| **RIN1** | Ras and Rab interactor 1 | up | **1.54** | 5.00E-04 |
| **KCNA5** | Potassium voltage-gated channel subfamily A member 5 | up | **1.53** | 3.15E-15 |
| **OSMR** | Oncostatin M receptor | up | **1.53** | 4.39E-10 |
| **TSPAN1** | Tetraspanin 1 | up | **1.52** | 1.87E-02 |
| **MGLL** | Monoglyceride lipase | up | **1.52** | 5.27E-09 |
| **SLC7A11** | Solute carrier family 7 member 11 | up | **1.52** | 6.66E-14 |
| **GABBR2** | Gamma-aminobutyric acid type B receptor subunit 2 | up | **1.51** | 2.82E-07 |
| **EPHA8** | EPH receptor A8 | up | **1.50** | 2.43E-05 |

| | | | | |
|---|---|---|---|---|
| **GJD2** | Gap junction protein delta 2 | down | **-3.92** | 6.10E-20 |
| **C14orf180** | Chromosome 14 open reading frame 180 | down | **-3.24** | 8.78E-16 |
| **TGM2** | Transglutaminase 2 | down | **-2.86** | 6.47E-06 |
| **OMG** | Oligodendrocyte myelin glycoprotein | down | **-2.80** | 1.53E-09 |
| **KCNAB1** | Potassium voltage-gated channel subfamily A member regulatory beta subunit 1 | down | **-2.41** | 3.67E-12 |
| **ITGA11** | Integrin subunit alpha 11 | down | **-2.19** | 7.41E-07 |
| **GFRA2** | GDNF family receptor alpha 2 | down | **-2.01** | 4.14E-07 |
| **GPR37L1** | G protein-coupled receptor 37 like 1 | down | **-2.01** | 2.81E-06 |
| **CYP2W1** | Cytochrome P450 family 2 subfamily W member 1 | down | **-1.94** | 1.98E-11 |
| **FGFR3** | Fibroblast growth factor receptor 3 | down | **-1.91** | 5.84E-19 |
| **OXTR** | Oxytocin receptor | down | **-1.85** | 1.73E-15 |
| **RHBDL2** | Rhomboid like 2 | down | **-1.84** | 2.52E-08 |
| **C10orf54** | Chromosome 10 open reading frame 54 | down | **-1.80** | 1.02E-13 |
| **SYT10** | Synaptotagmin 10 | down | **-1.75** | 3.49E-04 |
| **PRSS12** | Protease, serine 12 | down | **-1.74** | 4.60E-28 |
| **RGS9** | Regulator of G-protein signaling 9 | down | **-1.74** | 3.78E-06 |
| **MOG** | Myelin oligodendrocyte glycoprotein | down | **-1.73** | 2.64E-02 |
| **BMF** | Bcl2 modifying factor | down | **-1.69** | 8.82E-13 |
| **MFSD2A** | Major facilitator superfamily domain containing 2A | down | **-1.69** | 2.54E-11 |
| **ADRA2C** | Adrenoceptor alpha 2C | down | **-1.67** | 3.79E-20 |
| **CDC42EP1** | CDC42 effector protein 1 | down | **-1.60** | 1.93E-31 |
| **PHLDB2** | Pleckstrin homology like domain family B member 2 | down | **-1.59** | 2.92E-05 |
| **ABCB5** | ATP binding cassette subfamily B member 5 | down | **-1.59** | 3.70E-08 |
| **ENTPD8** | Ectonucleoside triphosphate diphosphohydrolase 8 | down | **-1.57** | 4.41E-10 |
| **RGS6** | Regulator of G-protein signaling 6 | down | **-1.57** | 5.04E-03 |
| **UNC5B** | Unc-5 netrin receptor B | down | **-1.56** | 4.34E-19 |
| **CDC42EP2** | CDC42 effector protein 2 | down | **-1.55** | 3.78E-12 |
| **CA2** | Carbonic anhydrase 2 | down | **-1.55** | 2.48E-06 |
| **AQP3** | Aquaporin 3 (Gill blood group) | down | **-1.54** | 1.97E-02 |
| **SMPD3** | Sphingomyelin phosphodiesterase 3 | down | **-1.53** | 8.77E-03 |
| **PLXNB3** | Plexin B3 | down | **-1.52** | 4.18E-11 |
| **ABCG1** | ATP binding cassette subfamily G member 1 | down | **-1.52** | 5.50E-18 |

| | | | | | | |
|---|---|---|---|---|---|---|
| | | **GRIK3** | Glutamate ionotropic receptor kainate type subunit 3 | down | **-1.51** | 9.06E-06 |
| | | **CA9** | Carbonic anhydrase 9 | down | **-1.50** | 7.90E-24 |
| | | | | | | |
| **Proteinaceous extracellular matrix** | GO:0005578 | **MMP1** | Matrix metallopeptidase 1 | up | **6.06** | 2.25E-08 |
| | | **ANGPTL4** | Angiopoietin like 4 | up | **3.56** | 2.07E-31 |
| | | **ECM1** | Extracellular matrix protein 1 | up | **1.82** | 3.16E-06 |
| | | **SPARCL1** | SPARC like 1 | up | **1.53** | 2.85E-08 |
| | | **AMTN** | Amelotin | down | **-2.37** | 1.93E-08 |
| | | **ADAMTS5** | ADAM metallopeptidase with thrombospondin type 1 motif 5 | down | **-2.24** | 3.58E-08 |
| | | **MGP** | Matrix Gla protein | down | **-2.21** | 5.47E-07 |
| | | **HAPLN3** | Hyaluronan and proteoglycan link protein 3 | down | **-2.00** | 8.82E-05 |
| | | **MMP11** | Matrix metallopeptidase 11 | down | **-1.76** | 9.27E-23 |
| | | **WNT2B** | Wnt family member 2B | down | **-1.74** | 2.06E-06 |
| | | **LUM** | Lumican | down | **-1.72** | 6.80E-05 |
| | | **ADAMTS15** | ADAM metallopeptidase with thrombospondin type 1 motif 15 | down | **-1.67** | 1.10E-04 |
| | | | | | | |
| **Spindle** | GO:0005819 | **KBTBD8** | Kelch repeat and BTB domain containing 8 | up | **1.67** | 1.62E-06 |
| | | **RPS6KA2** | Ribosomal protein S6 kinase A2 | up | **1.54** | 6.36E-26 |
| | | **KIF20A** | Kinesin family member 20A | down | **-2.04** | 5.94E-08 |
| | | **PLK1** | Polo like kinase 1 | down | **-1.72** | 2.43E-04 |
| | | **CENPF** | Centromere protein F | down | **-1.63** | 2.37E-05 |
| | | **TPX2** | TPX2, microtubule nucleation factor | down | **-1.60** | 1.84E-10 |
| | | **CDC20** | Cell division cycle 20 | down | **-1.52** | 1.12E-03 |
| | | **KIFC1** | Kinesin family member C1 | down | **-1.50** | 4.13E-05 |
| | | | | | | |
| **Extracellular space** | GO:0005615 | **ANGPTL4** | Angiopoietin like 4 | up | **3.56** | 2.07E-31 |
| | | **SRPX2** | Sushi repeat containing protein, X-linked 2 | up | **3.54** | 1.92E-11 |
| | | **DKK1** | Dickkopf WNT signaling pathway inhibitor 1 | up | **2.47** | 3.24E-08 |
| | | **PTX3** | Pentraxin 3 | up | **2.14** | 3.89E-05 |
| | | **SERPINE2** | Serpin family E member 2 | up | **2.12** | 3.52E-09 |

| | | | | | | |
|---|---|---|---|---|---|---|
| | | **FGF12** | Fibroblast growth factor 12 | up | **2.03** | 3.17E-08 |
| | | **ANXA1** | Annexin A1 | up | **1.93** | 1.17E-08 |
| | | **ECM1** | Extracellular matrix protein 1 | up | **1.82** | 3.16E-06 |
| | | **IL6** | Interleukin 6 | up | **1.76** | 3.15E-05 |
| | | **LAMC2** | Laminin subunit gamma 2 | up | **1.76** | 5.04E-12 |
| | | **HILPDA** | Hypoxia inducible lipid droplet associated | up | **1.76** | 2.88E-09 |
| | | **SERPINA3** | Serpin family A member 3 | up | **1.62** | 3.90E-07 |
| | | **IL33** | Interleukin 33 | up | **1.62** | 2.89E-15 |
| | | **TNFSF18** | Tumor necrosis factor superfamily member 18 | up | **1.59** | 8.30E-04 |
| | | **C1QTNF1** | C1q and tumor necrosis factor related protein 1 | up | **1.56** | 9.15E-14 |
| | | **SERPINE1** | Serpin family E member 1 | up | **1.54** | 2.46E-03 |
| | | **SPARCL1** | SPARC like 1 | up | **1.53** | 2.85E-08 |
| | | **ADAMTS5** | ADAM metallopeptidase with thrombospondin type 1 motif 5 | down | **-2.24** | 3.58E-08 |
| | | **ACTA2** | Actin, alpha 2, smooth muscle, aorta | down | **-2.13** | 4.92E-19 |
| | | **KRT81** | Keratin 81 | down | **-2.06** | 4.57E-03 |
| | | **HAPLN3** | Hyaluronan and proteoglycan link protein 3 | down | **-2.00** | 8.82E-05 |
| | | **WNT2B** | Wnt family member 2B | down | **-1.74** | 2.06E-06 |
| | | **LUM** | Lumican | down | **-1.72** | 6.80E-05 |
| | | **INHBB** | Inhibin beta B subunit | down | **-1.68** | 4.33E-13 |
| | | **ADAMTS15** | ADAM metallopeptidase with thrombospondin type 1 motif 15 | down | **-1.67** | 1.10E-04 |
| | | **SEMA3D** | Semaphorin 3D | down | **-1.65** | 6.47E-07 |
| | | **COL1A1** | Collagen type I alpha 1 chain | down | **-1.64** | 4.98E-03 |
| | | **SST** | Somatostatin | down | **-1.60** | 8.94E-03 |
| | | **CA2** | Carbonic anhydrase 2 | down | **-1.55** | 2.48E-06 |
| | | **EDN2** | Endothelin 2 | down | **-1.54** | 4.96E-12 |
| | | **MFGE8** | Milk fat globule-EGF factor 8 protein | down | **-1.52** | 9.70E-30 |
| | | **COL3A1** | Collagen type III alpha 1 chain | down | **-1.52** | 1.44E-13 |
| | | **PCSK2** | Proprotein convertase subtilisin/kexin type 2 | down | **-1.51** | 2.35E-03 |
| | | **SRPX2** | Sushi repeat containing protein, X-linked 2 | up | **3.54** | 1.92E-11 |
| **Cell surface** | GO:0009986 | **CLMP** | CXADR like membrane protein | up | **2.17** | 1.94E-07 |
| | | **EFCAB13** | EF-hand calcium binding domain 13 | up | **1.94** | 2.60E-06 |

| | | | | | | |
|---|---|---|---|---|---|---|
| | | **ANXA1** | Annexin A1 | up | **1.93** | 1.17E-08 |
| | | **HILPDA** | Hypoxia inducible lipid droplet associated | up | **1.76** | 2.88E-09 |
| | | **PRLR** | Prolactin receptor | up | **1.66** | 1.57E-18 |
| | | **CXCR4** | C-X-C motif chemokine receptor 4 | up | **1.64** | 1.05E-10 |
| | | **TNFSF18** | Tumor necrosis factor superfamily member 18 | up | **1.59** | 8.30E-04 |
| | | **LAYN** | Layilin | up | **1.55** | 4.50E-10 |
| | | **CD44** | CD44 molecule (Indian blood group) | up | **1.54** | 2.73E-09 |
| | | **IQGAP2** | IQ motif containing GTPase activating protein 2 | up | **1.52** | 1.10E-24 |
| | | **SLC7A11** | Solute carrier family 7 member 11 | up | **1.52** | 6.66E-14 |
| | | **CYP2W1** | Cytochrome P450 family 2 subfamily W member 1 | down | **-1.94** | 1.98E-11 |
| | | **FGFR3** | Fibroblast growth factor receptor 3 | down | **-1.91** | 5.84E-19 |
| | | **C10orf54** | Chromosome 10 open reading frame 54 | down | **-1.80** | 1.02E-13 |
| | | **ADAMTS15** | ADAM metallopeptidase with thrombospondin type 1 motif 15 | down | **-1.67** | 1.10E-04 |
| | | **PLXNB3** | Plexin B3 | down | **-1.52** | 4.18E-11 |
| | | | | | | |
| **Extracellular region** | GO:0005576 | **MMP1** | Matrix metallopeptidase 1 | up | **6.06** | 2.25E-08 |
| | | **ANGPTL4** | Angiopoietin like 4 | up | **3.56** | 2.07E-31 |
| | | **IL7R** | Interleukin 7 receptor | up | **3.40** | 3.11E-08 |
| | | **PLIN2** | Perilipin 2 | up | **2.83** | 3.52E-64 |
| | | **DKK1** | Dickkopf WNT signaling pathway inhibitor 1 | up | **2.47** | 3.24E-08 |
| | | **PTX3** | Pentraxin 3 | up | **2.14** | 3.89E-05 |
| | | **SERPINE2** | Serpin family E member 2 | up | **2.12** | 3.52E-09 |
| | | **ANXA1** | Annexin A1 | up | **1.93** | 1.17E-08 |
| | | **ISM2** | Isthmin 2 | up | **1.89** | 4.09E-05 |
| | | **ISG15** | ISG15 ubiquitin-like modifier | up | **1.88** | 3.91E-04 |
| | | **CDCP1** | CUB domain containing protein 1 | up | **1.84** | 3.65E-12 |
| | | **ECM1** | Extracellular matrix protein 1 | up | **1.82** | 3.16E-06 |
| | | **IL6** | Interleukin 6 | up | **1.76** | 3.15E-05 |
| | | **LAMC2** | Laminin subunit gamma 2 | up | **1.76** | 5.04E-12 |
| | | **PRLR** | Prolactin receptor | up | **1.66** | 1.57E-18 |
| | | **IL33** | Interleukin 33 | up | **1.62** | 2.89E-15 |
| | | **SERPINA3** | Serpin family A member 3 | up | **1.62** | 3.90E-07 |

| | | | | | | |
|---|---|---|---|---|---|---|
| | | **RAET1G** | Retinoic acid early transcript 1G | up | **1.60** | 1.87E-02 |
| | | **C1QTNF1** | C1q and tumor necrosis factor related protein 1 | up | **1.56** | 9.15E-14 |
| | | **SERPINE1** | Serpin family E member 1 | up | **1.54** | 2.46E-03 |
| | | **ADAMTS5** | ADAM metallopeptidase with thrombospondin type 1 motif 5 | down | **-2.24** | 3.58E-08 |
| | | **INHBA** | Inhibin beta A subunit | down | **-1.96** | 6.23E-03 |
| | | **FGFR3** | Fibroblast growth factor receptor 3 | down | **-1.91** | 5.84E-19 |
| | | **MMP11** | Matrix metallopeptidase 11 | down | **-1.76** | 9.27E-23 |
| | | **WNT2B** | Wnt family member 2B | down | **-1.74** | 2.06E-06 |
| | | **LUM** | Lumican | down | **-1.72** | 6.80E-05 |
| | | **INHBB** | Inhibin beta B subunit | down | **-1.68** | 4.33E-13 |
| | | **COL1A1** | Collagen type I alpha 1 chain | down | **-1.64** | 4.98E-03 |
| | | **SST** | Somatostatin | down | **-1.60** | 8.94E-03 |
| | | **MFAP4** | Microfibrillar associated protein 4 | down | **-1.55** | 2.66E-07 |
| | | **EDN2** | Endothelin 2 | down | **-1.54** | 4.96E-12 |
| | | **THBS2** | Thrombospondin 2 | down | **-1.53** | 2.41E-06 |
| | | **MFGE8** | Milk fat globule-EGF factor 8 protein | down | **-1.52** | 9.70E-30 |
| | | **COL3A1** | Collagen type III alpha 1 chain | down | **-1.52** | 1.44E-13 |
| | | **CA11** | Carbonic anhydrase 11 | down | **-1.51** | 8.23E-15 |
| | | **NTN1** | Netrin 1 | down | **-1.51** | 3.14E-06 |
| | | **MMP1** | Matrix metallopeptidase 1 | down | **-6.06** | 2.25E-08 |
| | | | | | | |
| **Integral component of plasma membrane** | GO:0005887 | **ABCC3** | ATP binding cassette subfamily C member 3 | up | **2.60** | 6.14E-08 |
| | | **PLAUR** | Plasminogen activator, urokinase receptor | up | **2.38** | 1.14E-10 |
| | | **TM4SF1** | Transmembrane 4 L six family member 1 | up | **2.30** | 1.04E-05 |
| | | **SCNN1A** | Sodium channel epithelial 1 alpha subunit | up | **2.21** | 3.92E-26 |
| | | **SLC24A4** | Solute carrier family 24 member 4 | up | **2.12** | 1.61E-05 |
| | | **HVCN1** | Hydrogen voltage gated channel 1 | up | **1.97** | 7.60E-08 |
| | | **PTGER2** | Prostaglandin E receptor 2 | up | **1.95** | 1.63E-26 |
| | | **EFCAB13** | EF-hand calcium binding domain 13 | up | **1.94** | 2.60E-06 |
| | | **MME** | Membrane metallo-endopeptidase | up | **1.79** | 8.51E-07 |
| | | **ST3GAL5** | ST3 beta-galactoside alpha-2,3-Sialyltransferase 5 | up | **1.75** | 1.86E-13 |
| | | **SLC3A1** | Solute carrier family 3 member 1 | up | **1.72** | 1.76E-03 |

| | | | | | | |
|---|---|---|---|---|---|---|
| | | **SLC22A6** | Solute carrier family 22 member 6 | up | **1.63** | 1.17E-05 |
| | | **C1QTNF1** | C1q and tumor necrosis factor related protein 1 | up | **1.56** | 9.15E-14 |
| | | **SLC18A3** | Solute carrier family 18 member A3 | up | **1.55** | 1.33E-26 |
| | | **CD44** | CD44 molecule (Indian blood group) | up | **1.54** | 2.73E-09 |
| | | **ABCA4** | ATP binding cassette subfamily A member 4 | up | **1.54** | 5.36E-08 |
| | | **SLC16A6** | Solute carrier family 16 member 6 | up | **1.53** | 4.76E-05 |
| | | **KCNA5** | Potassium voltage-gated channel subfamily A member 5 | up | **1.53** | 3.15E-15 |
| | | **SLC7A11** | Solute carrier family 7 member 11 | up | **1.52** | 6.66E-14 |
| | | **TSPAN1** | Tetraspanin 1 | up | **1.52** | 1.87E-02 |
| | | **GABBR2** | Gamma-aminobutyric acid type B receptor subunit 2 | up | **1.51** | 2.82E-07 |
| | | **EPHA8** | EPH receptor A8 | up | **1.50** | 2.43E-05 |
| | | **NTRK3** | Neurotrophic receptor tyrosine kinase 3 | down | **-1.97** | 1.20E-06 |
| | | **FGFR3** | Fibroblast growth factor receptor 3 | down | **-1.91** | 5.84E-19 |
| | | **OXTR** | Oxytocin receptor | down | **-1.85** | 1.73E-15 |
| | | **MFSD2A** | Major facilitator superfamily domain containing 2A | down | **-1.69** | 2.54E-11 |
| | | **ADRA2C** | Adrenoceptor alpha 2C | down | **-1.67** | 3.79E-20 |
| | | **ABCB5** | ATP binding cassette subfamily B member 5 | down | **-1.59** | 3.70E-08 |
| | | **AQP3** | Aquaporin 3 (Gill blood group) | down | **-1.54** | 1.97E-02 |
| | | **PLXNB3** | Plexin B3 | down | **-1.52** | 4.18E-11 |
| | | **ABCG1** | ATP binding cassette subfamily G member 1 | down | **-1.52** | 5.50E-18 |
| | | **GRIK3** | Glutamate ionotropic receptor kainate type subunit 3 | down | **-1.51** | 9.06E-06 |
| | | | | | | |
| **Focal adhesion** | GO:0005925 | **PLAUR** | Plasminogen activator, urokinase receptor | up | **2.38** | 1.14E-10 |
| | | **HMGA1** | High mobility group AT-hook 1 | up | **1.97** | 7.42E-07 |
| | | **EFCAB13** | EF-hand calcium binding domain 13 | up | **1.94** | 2.60E-06 |
| | | **ANXA1** | Annexin A1 | up | **1.93** | 1.17E-08 |
| | | **MME** | Membrane metallo-endopeptidase | up | **1.79** | 8.51E-07 |
| | | **LAYN** | Layilin | up | **1.55** | 4.50E-10 |
| | | **CD44** | CD44 molecule (Indian blood group) | up | **1.54** | 2.73E-09 |
| | | **CNN1** | Calponin 1 | down | **-4.32** | 6.93E-06 |

| | | | | | | |
|---|---|---|---|---|---|---|
| | | **TGM2** | Transglutaminase 2 | down | **-2.86** | 6.47E-06 |
| | | **ITGA11** | Integrin subunit alpha 11 | down | **-2.19** | 7.41E-07 |
| | | **FGFR3** | Fibroblast growth factor receptor 3 | down | **-1.91** | 5.84E-19 |
| | | **CDC42EP1** | CDC42 effector protein 1 | down | **-1.60** | 1.93E-31 |
| | | **PHLDB2** | Pleckstrin homology like domain family B member 2 | down | **-1.59** | 2.92E-05 |
| | | | | | | |
| **Midbody** | GO:0030496 | **KIF20A** | Kinesin family member 20A | down | **-2.04** | 5.94E-08 |
| | | **NEK2** | NIMA related kinase 2 | down | **-1.77** | 2.50E-03 |
| | | **PLK1** | Polo like kinase 1 | down | **-1.72** | 2.43E-04 |
| | | **KIF4A** | Kinesin family member 4A | down | **-1.71** | 6.57E-05 |
| | | **CENPF** | Centromere protein F | down | **-1.63** | 2.37E-05 |
| | | **CDK1** | Cyclin dependent kinase 1 | down | **-1.62** | 4.47E-10 |
| | | **ASPM** | Abnormal spindle microtubule assembly | down | **-1.58** | 1.97E-06 |
| | | | | | | |
| **Cytosol** | GO:0005829 | **AKR1C1** | Aldo-keto reductase family 1 member C1 | up | **16.20** | 8.08E-17 |
| | | **HSPA6** | Heat shock protein family A member 6 | up | **13.96** | 9.08E-12 |
| | | **AKR1B10** | Aldo-keto reductase family 1 member B10 | up | **7.56** | 1.07E-12 |
| | | **FOSL1** | FOS like 1, AP-1 transcription factor subunit | up | **3.81** | 3.16E-08 |
| | | **DUSP6** | Dual specificity phosphatase 6 | up | **2.85** | 1.83E-07 |
| | | **PLIN2** | Perilipin 2 | up | **2.83** | 3.52E-64 |
| | | **NDRG4** | NDRG family member 4 | up | **2.71** | 1.93E-12 |
| | | **SERPINE2** | Serpin family E member 2 | up | **2.12** | 3.52E-09 |
| | | **HMGA1** | High mobility group AT-hook 1 | up | **1.97** | 7.42E-07 |
| | | **CALB2** | Calbindin 2 | up | **1.96** | 7.57E-03 |
| | | **PHLDA1** | Pleckstrin homology like domain family A member 1 | up | **1.88** | 1.08E-07 |
| | | **MT2A** | Metallothionein 2A | up | **1.88** | 4.02E-11 |
| | | **ISG15** | ISG15 ubiquitin-like modifier | up | **1.88** | 3.91E-04 |
| | | **SPRY2** | Sprouty RTK signaling antagonist 2 | up | **1.77** | 3.61E-07 |
| | | **NQO1** | NAD(P)H quinone dehydrogenase 1 | up | **1.72** | 9.60E-09 |
| | | **TRIB3** | Tribbles pseudokinase 3 | up | **1.71** | 2.94E-06 |
| | | **PMAIP1** | Phorbol-12-myristate-13-acetate-induced protein 1 | up | **1.61** | 1.21E-06 |
| | | **RLBP1** | Retinaldehyde binding protein 1 | up | **1.57** | 6.70E-10 |

| | | | | |
|---|---|---|---|---|
| **UPP1** | Uridine phosphorylase 1 | up | **1.57** | 1.64E-04 |
| **PCP4** | Purkinje cell protein 4 | up | **1.55** | 2.98E-38 |
| **RPS6KA2** | Ribosomal protein S6 kinase A2 | up | **1.54** | 6.36E-26 |
| **CHAT** | Choline O-acetyltransferase | up | **1.53** | 2.92E-04 |
| **IQGAP2** | IQ motif containing GTPase activating protein 2 | up | **1.52** | 1.10E-24 |
| **MGLL** | Monoglyceride lipase | up | **1.52** | 5.27E-09 |
| **TGM2** | Transglutaminase 2 | down | **-2.86** | 6.47E-06 |
| **RPL3L** | Ribosomal protein L3 like | down | **-2.55** | 1.32E-10 |
| **KCNAB1** | Potassium voltage-gated channel subfamily A member regulatory beta subunit 1 | down | **-2.41** | 3.67E-12 |
| **FGD3** | FYVE, RhoGEF and PH domain containing 3 | down | **-2.16** | 4.81E-09 |
| **ACTA2** | Actin, alpha 2, smooth muscle, aorta | down | **-2.13** | 4.92E-19 |
| **TNNT2** | Troponin T2, cardiac type | down | **-2.09** | 5.81E-11 |
| **CARNS1** | Carnosine synthase 1 | down | **-2.02** | 5.05E-16 |
| **CCNA1** | Cyclin A1 | down | **-1.92** | 1.29E-07 |
| **AP1M2** | Adaptor related protein complex 1 mu 2 subunit | down | **-1.91** | 2.49E-11 |
| **PPP1R14A** | Protein phosphatase 1 regulatory inhibitor subunit 14A | down | **-1.88** | 3.09E-07 |
| **ERF** | ETS2 repressor factor | down | **-1.88** | 1.18E-23 |
| **ATP6V1B1** | ATPase $H^+$ transporting V1 subunit B1 | down | **-1.85** | 5.46E-07 |
| **ANKRD1** | Ankyrin repeat domain 1 | down | **-1.85** | 6.52E-03 |
| **SPC24** | SPC24, NDC80 kinetochore complex component | down | **-1.84** | 4.74E-07 |
| **BUB1B** | BUB1 mitotic checkpoint serine/threonine kinase B | down | **-1.81** | 2.76E-11 |
| **NEK2** | NIMA related kinase 2 | down | **-1.77** | 2.50E-03 |
| **PLK1** | Polo like kinase 1 | down | **-1.72** | 2.43E-04 |
| **KIF4A** | Kinesin family member 4A | down | **-1.71** | 6.57E-05 |
| **BMF** | Bcl2 modifying factor | down | **-1.69** | 8.82E-13 |
| **DEPDC1B** | DEP domain containing 1B | down | **-1.65** | 1.03E-04 |
| **CENPF** | Centromere protein F | down | **-1.63** | 2.37E-05 |
| **CDK1** | Cyclin dependent kinase 1 | down | **-1.62** | 4.47E-10 |
| **TPX2** | TPX2, microtubule nucleation factor | down | **-1.60** | 1.84E-10 |
| **CDCA3** | Cell division cycle associated 3 | down | **-1.59** | 4.27E-03 |

| | | | | | | |
|---|---|---|---|---|---|---|
| | | **CENPM** | Centromere protein M | down | **-1.58** | 5.36E-03 |
| | | **CDKN2C** | Cyclin dependent kinase inhibitor 2C | down | **-1.57** | 1.97E-04 |
| | | **RGS6** | Regulator of G-protein signaling 6 | down | **-1.57** | 5.04E-03 |
| | | **MLXIPL** | MLX interacting protein like | down | **-1.56** | 3.07E-04 |
| | | **CDC42EP2** | CDC42 effector protein 2 | down | **-1.55** | 3.78E-12 |
| | | **CA2** | Carbonic anhydrase 2 | down | **-1.55** | 2.48E-06 |
| | | **SPC25** | SPC25, NDC80 kinetochore complex component | down | **-1.54** | 3.80E-02 |
| | | **CCND2** | Cyclin D2 | down | **-1.54** | 2.85E-17 |
| | | **DIAPH3** | Diaphanous related formin 3 | down | **-1.53** | 1.22E-08 |
| | | **CDC20** | Cell division cycle 20 | down | **-1.52** | 1.12E-03 |
| | | **PYGM** | Phosphorylase, glycogen, muscle | down | **-1.51** | 1.45E-24 |
| | | **GTSE1** | G2 and S-phase expressed 1 | down | **-1.51** | 6.60E-05 |
| **Basolateral plasma membrane** | GO:0016323 | **NDRG4** | NDRG family member 4 | up | **2.71** | 1.93E-12 |
| | | **ANXA1** | Annexin A1 | up | **1.93** | 1.17E-08 |
| | | **SLC22A6** | Solute carrier family 22 member 6 | up | **1.63** | 1.17E-05 |
| | | **ATP6V1B1** | ATPase $H^+$ transporting V1 subunit B1 | down | **-1.85** | 5.46E-07 |
| | | **CA2** | Carbonic anhydrase 2 | down | **-1.55** | 2.48E-06 |
| | | **AQP3** | Aquaporin 3 (Gill blood group) | down | **-1.54** | 1.97E-02 |
| | | **CA11** | Carbonic anhydrase 11 | down | **-1.51** | 8.23E-15 |
| | | **CA9** | Carbonic anhydrase 9 | down | **-1.50** | 7.90E-24 |
| **Spindle microtubule** | GO:0005876 | **PLK1** | Polo like kinase 1 | down | **-1.72** | 2.43E-04 |
| | | **KIF4A** | Kinesin family member 4A | down | **-1.71** | 6.57E-05 |
| | | **CAPN6** | Calpain 6 | down | **-1.67** | 3.35E-12 |
| | | **CDK1** | Cyclin dependent kinase 1 | down | **-1.62** | 4.47E-10 |
| **Extrinsic component of membrane** | GO:0019898 | **PLAUR** | Plasminogen activator, urokinase receptor | up | **2.38** | 1.14E-10 |
| | | **ANXA1** | Annexin A1 | up | **1.93** | 1.17E-08 |
| | | **MGLL** | Monoglyceride lipase | up | **1.52** | 5.27E-09 |
| | | **GFRA2** | GDNF family receptor alpha 2 | down | **-2.01** | 4.14E-07 |
| | | **RGS6** | Regulator of G-protein signaling 6 | down | **-1.57** | 5.04E-03 |

| | | | | | | |
|---|---|---|---|---|---|---|
| **Condensed chromosome kinetochore** | GO:0000777 | **SPC24** | SPC24, NDC80 kinetochore complex component | down | **-1.84** | 4.74E-07 |
| | | **BUB1B** | BUB1 mitotic checkpoint serine/threonine kinase B | down | **-1.81** | 2.76E-11 |
| | | **NEK2** | NIMA related kinase 2 | down | **-1.77** | 2.50E-03 |
| | | **CENPM** | Centromere protein M | down | **-1.58** | 5.36E-03 |
| | | **SPC25** | SPC25, NDC80 kinetochore complex component | down | **-1.54** | 3.80E-02 |
| **Microvillus** | GO:0005902 | **IQGAP2** | IQ motif containing GTPase activating protein 2 | up | **1.52** | 1.10E-24 |
| | | **OXTR** | Oxytocin receptor | down | **-1.85** | 1.73E-15 |
| | | **ATP6V1B1** | ATPase $H^{+}$ transporting V1 subunit B1 | down | **-1.85** | 5.46E-07 |
| | | **CA2** | Carbonic anhydrase 2 | down | **-1.55** | 2.48E-06 |
| **Integral component of Golgi membrane** | GO:0030173 | **B4GALNT1** | Beta-1,4-N-acetyl-galactosaminyltransferase 1 | up | **2.46** | 3.94E-08 |
| | | **ST3GAL5** | ST3 beta-galactoside alpha-2,3-sialyltransferase 5 | up | **1.75** | 1.86E-13 |
| | | **ACER2** | Alkaline ceramidase 2 | up | **1.67** | 2.27E-02 |
| | | **ST3GAL4** | ST3 beta-galactoside alpha-2,3-sialyltransferase 4 | down | **-1.54** | 1.14E-17 |
| **Apical plasma membrane** | GO:0016324 | **SCNN1A** | Sodium channel epithelial 1 alpha subunit | up | **2.21** | 3.92E-26 |
| | | **RAB27B** | RAB27B, member RAS oncogene family | up | **2.05** | 2.46E-14 |
| | | **ANXA1** | Annexin A1 | up | **1.93** | 1.17E-08 |
| | | **KCNMA1** | Potassium calcium-activated channel subfamily M alpha 1 | up | **1.92** | 6.93E-10 |
| | | **ATP1B2** | ATPase $Na^{+}/K^{+}$ transporting subunit beta 2 | up | **1.57** | 1.62E-10 |
| | | **OSMR** | Oncostatin M receptor | up | **1.53** | 4.39E-10 |
| | | **OXTR** | Oxytocin receptor | down | **-1.85** | 1.73E-15 |

| | | | | |
|---|---|---|---|---|
| **ATP6V1B1** | ATPase $H^+$ transporting V1 subunit B1 | down | **-1.85** | 5.46E-07 |
| **TMEM235** | Transmembrane protein 235 | down | **-1.52** | 3.22E-04 |

GO, gene ontology; up, up-regulated genes in the cellular component gene ontology term; down, down-regulated genes in the cellular component gene ontology term; DEG, differentially expressed gene; FC, fold-change.

**Supplementary Table S2** RNA-Seq transcriptomic analysis predicted aldosterone up-regulated genes. Aldosterone-treated samples compared to untreated control samples.

**Aldosterone $10^{-7}$ M** **Up-regulated genes**

| *P*-value | FC | Gene name | Description |
|---|---|---|---|
| 2.68E-04 | **3.55** | **EHF** | ETS homologous factor |
| 2.87E-06 | **3.35** | **ZBTB16** | Zinc finger and BTB domain containing 16 |
| 1.87E-03 | **2.99** | **SERPINA3** | Serpin family A member 3 |
| 1.80E-05 | **2.97** | **POU5F1** | POU class 5 homeobox 1 |
| 2.30E-04 | **2.71** | **LTBR** | Lymphotoxin beta receptor |
| 7.89E-03 | **2.53** | **LINC01844** | *Long intergenic non-protein coding RNA 1844 (LOC101926975)* |
| 4.88E-03 | **2.48** | **AL021395.1** | *Long intergenic non-coding RNA* |
| 3.10E-03 | **2.46** | **ARMC12** | Armadillo repeat containing 12 |
| 4.73E-03 | **2.45** | **AC112206.2** | *Long intergenic non-protein coding RNA (LOC101928858)* |
| 1.40E-03 | **2.41** | **LINC01603** | *Long intergenic non-protein coding RNA 1603* |
| 1.02E-06 | **2.22** | **SCNN1A** | Sodium channel epithelial 1 alpha subunit |
| 4.69E-03 | **2.18** | **RP11-1024P17.1** | *Long non-protein coding RNA* |
| 6.77E-03 | **2.16** | **PLAUR** | Plasminogen activator, urokinase receptor |
| 2.86E-02 | **2.15** | **GCK** | Glucokinase |
| 2.70E-02 | **2.14** | **TMPRSS15** | Transmembrane protease, serine 15 |
| 6.85E-04 | **2.13** | **PCDH1** | Protocadherin 1 |
| 4.54E-02 | **2.13** | **AGXT** | Alanine-glyoxylate aminotransferase |
| 2.06E-02 | **2.10** | **LINC00176** | *Long intergenic non-protein coding RNA 176* |
| 1.15E-02 | **2.02** | **GRID2** | Glutamate ionotropic receptor delta type subunit 2 |
| 2.57E-02 | **2.00** | **TNFSF18** | Tumor necrosis factor superfamily member 18 |
| 2.70E-03 | **2.00** | **LRRC37A17P** | Leucine rich repeat containing 37 member A17, pseudogene |
| 3.65E-02 | **2.00** | **TAC4** | Tachykinin 4 (hemokinin) |
| 7.27E-03 | **1.99** | **AC132938.5** | *Long non-protein coding RNA* |
| 1.93E-02 | **1.95** | **MPP4** | Membrane palmitoylated protein 4 |
| 4.04E-02 | **1.95** | **MYBPC2** | Myosin binding protein C, fast type |
| 2.24E-02 | **1.94** | **RNU6ATAC18P** | RNA, U6atac small nuclear 18, pseudogene |
| 8.17E-03 | **1.93** | **SEZ6L** | Seizure related 6 homolog like |
| 1.56E-03 | **1.92** | **AC009542.2** | *Transcribed unprocessed pseudogene* |
| 1.58E-03 | **1.90** | **FKBP5** | FK506 binding protein 5 |
| 3.69E-03 | **1.89** | **FOSL1** | FOS like 1, AP-1 transcription factor subunit |
| 3.93E-02 | **1.88** | **AL390066.1** | *Long intergenic non-protein coding RNA* |
| 2.87E-03 | **1.87** | **OMD** | Osteomodulin |
| 4.26E-03 | **1.87** | **HVCN1** | Hydrogen voltage gated channel 1 |
| 4.91E-04 | **1.87** | **SLC3A1** | Solute carrier family 3 member 1 |
| 2.85E-04 | **1.86** | **DUSP8P5** | Dual specificity phosphatase 8 pseudogene 5 |
| 3.34E-03 | **1.85** | **DRGX** | Dorsal root ganglia homeobox |
| 3.48E-05 | **1.84** | **AP001596.1** | *Antisense RNA* |
| 4.72E-03 | **1.82** | **SPRY4** | Sprouty RTK signaling antagonist 4 |
| 2.50E-04 | **1.81** | **S100A2** | S100 calcium binding protein A2 |
| 4.40E-03 | **1.81** | **AC005162.3** | *Antisense RNA* |
| 8.65E-03 | **1.80** | **AC124312.5** | *Long non-protein coding RNA* |
| 4.34E-03 | **1.74** | **CYYR1-AS1** | Cysteine and tyrosine rich 1 antisense RNA 1 |
| 2.64E-02 | **1.74** | **ADH1B** | Alcohol dehydrogenase 1B (class I), beta polypeptide |
| 1.09E-02 | **1.73** | **SVEP1** | Sushi, von Willebrand factor type A, EGF and pentraxin domain containing 1 |

| | | | |
|---|---|---|---|
| 2.61E-03 | **1.71** | **RXRG** | Retinoid X receptor gamma |
| 3.12E-02 | **1.71** | **ITGB3** | Integrin subunit beta 3 |
| 3.97E-06 | **1.71** | **TMEM72** | Transmembrane protein 72 |
| 4.23E-02 | **1.71** | **P2RY6** | Pyrimidinergic receptor P2Y6 |
| 1.81E-02 | **1.70** | **SNORD109A** | Small nucleolar RNA, C/D box 109A |
| 2.87E-02 | **1.70** | **LINC01443** | *Long intergenic non-protein coding RNA 1443* |
| 2.10E-02 | **1.70** | **AL353801.1** | *Long intergenic non-protein coding RNA* |
| 2.77E-02 | **1.70** | **EPHA8** | EPH receptor A8 |
| 6.41E-04 | **1.69** | **TMEM72-AS1** | TMEM72 antisense RNA 1 |
| 2.60E-02 | **1.68** | **NLRC3** | NLR family CARD domain containing 3 |
| 1.65E-02 | **1.68** | **RFTN2** | Raftlin family member 2 |
| 1.94E-02 | **1.68** | **PNMT** | Phenylethanolamine N-methyltransferase |
| 3.27E-03 | **1.67** | **S100A3** | S100 calcium binding protein A3 |
| 8.81E-03 | **1.67** | **AC128688.2** | *Antisense RNA* |
| 3.01E-03 | **1.66** | **VWA5B2** | Von Willebrand factor A domain containing 5B2 |
| 1.54E-02 | **1.64** | **LINC00930** | *Long intergenic non-protein coding RNA 930* |
| 3.89E-02 | **1.63** | **AC025263.1** | *Antisense RNA (LOC101928002)* |
| 9.51E-04 | **1.62** | **GPRIN1** | G protein regulated inducer of neurite outgrowth 1 |
| 1.81E-06 | **1.61** | **MCTP2** | Multiple C2 and transmembrane domain containing 2 |
| 3.30E-02 | **1.60** | **AC024075.1** | *Sense intronic RNA* |
| 1.39E-02 | **1.60** | **PLAT** | Plasminogen activator, tissue type |
| 7.17E-03 | **1.58** | **GPR75** | G protein-coupled receptor 75 |
| 1.27E-04 | **1.58** | **AC083843.3** | *Long intergenic non-protein coding RNA* |
| 7.08E-03 | **1.57** | **DSCAML1** | DS cell adhesion molecule like 1 |
| 1.37E-05 | **1.57** | **DUSP6** | Dual specificity phosphatase 6 |
| 7.18E-06 | **1.57** | **PTGER2** | Prostaglandin E receptor 2 |
| 1.44E-02 | **1.57** | **AC245060.4** | *Transcribed unprocessed pseudogene* |
| 1.64E-02 | **1.57** | **ZNF114** | Zinc finger protein 114 |
| 8.84E-04 | **1.55** | **ATP6V0A4** | ATPase $H^+$ transporting V0 subunit a4 |
| 2.95E-03 | **1.55** | **OPRD1** | Opioid receptor delta 1 |
| 2.28E-03 | **1.55** | **S100A6** | S100 calcium binding protein A6 |
| 5.61E-03 | **1.55** | **ANKRD23** | Ankyrin repeat domain 23 |
| 2.41E-02 | **1.55** | **ABCC3** | ATP binding cassette subfamily C member 3 |
| 2.66E-02 | **1.54** | **PTX3** | Pentraxin 3 |
| 2.43E-03 | **1.54** | **FAM196B** | Family with sequence similarity 196 member B |
| 2.82E-03 | **1.54** | **LINC01238** | *Long intergenic non-protein coding RNA 1238* |
| 7.46E-03 | **1.54** | **HIST1H2BC** | Histone cluster 1, H2bc |
| 7.70E-03 | **1.53** | **HMGA1** | High mobility group AT-hook 1 |
| 2.06E-03 | **1.53** | **ITGA4** | Integrin subunit alpha 4 |
| 2.34E-03 | **1.52** | **CXCR4** | C-X-C motif chemokine receptor 4 |
| 1.27E-02 | **1.51** | **ITGAX** | Integrin subunit alpha X |
| 3.53E-06 | **1.51** | **PER1** | Period circadian clock 1 |
| 4.01E-02 | **1.51** | **C19orf71** | *Chromosome 19 open reading frame 71* |
| 1.47E-03 | **1.51** | **GNL3L** | G protein nucleolar 3 like |
| 5.68E-03 | **1.51** | **MGAM2** | Maltase-glucoamylase 2 (putative) |
| 6.91E-03 | **1.50** | **GHRLOS** | Ghrelin opposite strand/antisense RNA |

Genes are classified according to the fold change with a *P*-value and fold-change cut-off of $P < 0.05$ and FC > 1.5, respectively. FC, fold-change.

Supplementary Table S3 RNA-Seq transcriptomic analysis predicted aldosterone down-regulated genes. Aldosterone-treated samples compared to untreated control samples.

Aldosterone $10^{-7}$ M — Down-regulated genes

| *P*-value | FC | Gene name | Description |
|---|---|---|---|
| 2.06E-04 | -2.70 | SOCS2-AS1 | SOCS2 antisense RNA 1 |
| 1.20E-03 | -2.53 | SERPINB9P1 | Serpin family B member 9 pseudogene 1 |
| 1.36E-04 | -2.48 | LINC01164 | *Long intergenic non-protein coding RNA 1164* |
| 3.39E-03 | -2.31 | DOC2A | Double C2 domain alpha |
| 4.78E-03 | -2.30 | STK32A | Serine/threonine kinase 32A |
| 2.16E-03 | -2.19 | MPPED1 | Metallophosphoesterase domain containing 1 |
| 5.66E-03 | -2.16 | MGP | Matrix Gla protein |
| 8.28E-04 | -2.15 | AL589740.1 | *Long intergenic non-protein coding RNA (LOC101927314)* |
| 2.31E-02 | -2.11 | LINC00844 | *Long intergenic non-protein coding RNA 844* |
| 3.29E-02 | -2.09 | FMOD | Fibromodulin |
| 4.51E-04 | -2.05 | ABCB1 | ATP binding cassette subfamily B member 1 |
| 1.38E-03 | -2.04 | IL5RA | Interleukin 5 receptor subunit alpha |
| 4.87E-04 | -2.02 | RPRML | Reprimo like |
| 2.21E-03 | -2.01 | GDF7 | Growth differentiation factor 7 |
| 1.32E-03 | -1.97 | TMEM160 | Transmembrane protein 160 |
| 7.25E-03 | -1.96 | C19orf81 | *Chromosome 19 open reading frame 81* |
| 3.10E-03 | -1.96 | PCOLCE-AS1 | PCOLCE antisense RNA 1 |
| 4.59E-03 | -1.95 | SIX1 | SIX homeobox 1 |
| 3.45E-02 | -1.95 | LINC00639 | *Long intergenic non-protein coding RNA 639* |
| 9.35E-04 | -1.94 | CDC20P1 | Cell division cycle 20 pseudogene 1 |
| 1.28E-02 | -1.93 | AL603840.1 | *Long intergenic non-protein coding RNA* |
| 4.99E-03 | -1.92 | EVC2 | EvC ciliary complex subunit 2 |
| 2.06E-02 | -1.92 | RBFOX3 | RNA binding protein, fox-1 homolog 3 |
| 1.90E-03 | -1.92 | MAP3K7CL | MAP3K7 C-terminal like |
| 3.72E-02 | -1.89 | LINC01719 | *Long intergenic non-protein coding RNA 1719* |
| 2.77E-03 | -1.88 | NYAP2 | Neuronal tyrosine-phosphorylated phosphoinositide-3-kinase adaptor 2 |
| 3.57E-03 | -1.88 | NPW | Neuropeptide W |
| 1.10E-03 | -1.88 | C4orf48 | *Chromosome 4 open reading frame 48* |
| 3.20E-02 | -1.87 | PLP1 | Proteolipid protein 1 |
| 1.04E-03 | -1.86 | PCSK1N | Proprotein convertase subtilisin/kexin type 1 inhibitor |
| 3.76E-04 | -1.85 | EGR1 | Early growth response 1 |
| 1.80E-02 | -1.82 | LMX1A | LIM homeobox transcription factor 1 alpha |
| 1.66E-02 | -1.82 | FIGNL2 | Fidgetin like 2 |
| 1.03E-04 | -1.81 | SST | Somatostatin |
| 3.33E-02 | -1.81 | AL731563.3 | *Long intergenic non-protein coding RNA* |
| 3.62E-03 | -1.80 | SOX3 | SRY-box 3 |
| 1.66E-02 | -1.80 | GRIN3B | Glutamate ionotropic receptor NMDA type subunit 3B |
| 7.76E-04 | -1.80 | MAFA | MAF bZIP transcription factor A |
| 2.31E-03 | -1.80 | AC087741.3 | *Uncategorized gene* |
| 7.77E-03 | -1.79 | PNMA8C | PNMA family member 8C |
| 9.00E-03 | -1.79 | CCDC3 | Coiled-coil domain containing 3 |
| 9.23E-03 | -1.79 | MORN5 | MORN repeat containing 5 |
| 1.23E-02 | -1.79 | CTNNA2 | Catenin alpha 2 |
| 3.42E-02 | -1.78 | MYL3 | Myosin light chain 3 |
| 1.79E-02 | -1.78 | AC110285.7 | *Long non-protein coding RNA* |
| 5.10E-04 | -1.78 | CDC37L1-AS1 | CDC37L1 antisense RNA 1 (head to head) |

| | | | |
|---|---|---|---|
| 1.56E-02 | -1.76 | UNC5B-AS1 | UNC5B antisense RNA 1 |
| 3.21E-02 | -1.76 | AC100814.1 | *Long intergenic non-protein coding RNA* |
| 2.52E-02 | -1.75 | AC120053.1 | *Antisense RNA* |
| 3.56E-03 | -1.74 | AL441883.1 | *Antisense RNA* |
| 3.73E-02 | -1.73 | ACTG2 | Actin, gamma 2, smooth muscle, enteric |
| 1.03E-02 | -1.72 | CNN1 | Calponin 1 |
| 3.69E-02 | -1.72 | AC024681.1 | *Long non-protein coding RNA* |
| 2.25E-04 | -1.72 | RPL13AP5 | Ribosomal protein L13a pseudogene 5 |
| 1.86E-03 | -1.72 | PRKAG2-AS1 | PRKAG2 antisense RNA 1 |
| 3.19E-02 | -1.71 | AP000473.2 | *Long intergenic non-protein coding RNA* |
| 1.71E-02 | -1.70 | ADARB2 | Adenosine deaminase, RNA specific B2 (inactive) |
| 3.40E-05 | -1.70 | IGFBP3 | Insulin like growth factor binding protein 3 |
| 2.25E-03 | -1.70 | RASL12 | RAS like family 12 |
| 4.72E-02 | -1.69 | EGR2 | Early growth response 2 |
| 3.22E-04 | -1.67 | RTN4RL2 | Reticulon 4 receptor-like 2 |
| 1.70E-02 | -1.67 | GJD2 | Gap junction protein delta 2 |
| 5.39E-03 | -1.67 | SCGB1B2P | Secretoglobin family 1B member 2, pseudogene |
| 2.59E-03 | -1.66 | EEF1A1P6 | Eukaryotic translation elongation factor 1 alpha 1 pseudogene 6 |
| 4.60E-03 | -1.66 | ADAT3 | Adenosine deaminase, tRNA specific 3 |
| 1.38E-04 | -1.65 | LIX1 | Limb and CNS expressed 1 |
| 1.36E-02 | -1.65 | MXRA5Y | Matrix remodeling associated 5, Y-linked (pseudogene) |
| 1.01E-03 | -1.64 | PLPPR3 | Phospholipid phosphatase related 3 |
| 2.25E-02 | -1.64 | PTPRC | Protein tyrosine phosphatase, receptor type C |
| 7.16E-04 | -1.63 | AC004540.2 | *Antisense RNA (LOC105375304)* |
| 3.09E-02 | -1.62 | PGM5P4 | Phosphoglucomutase 5 pseudogene 4 |
| 6.55E-04 | -1.61 | GFRA2 | GDNF family receptor alpha 2 |
| 9.47E-03 | -1.61 | EFCAB5 | EF-hand calcium binding domain 5 |
| 6.71E-04 | -1.61 | TESMIN | Testis expressed metallothionein like protein |
| 1.13E-02 | -1.61 | RPS2P7 | Ribosomal protein S2 pseudogene 7 |
| 1.79E-02 | -1.60 | DRC1 | Dynein regulatory complex subunit 1 |
| 1.74E-02 | -1.60 | LINC02256 | *Long intergenic non-protein coding RNA 2256* |
| 2.24E-03 | -1.60 | GPR158 | G protein-coupled receptor 158 |
| 1.65E-03 | -1.59 | AP000892.3 | *Uncategorized gene* |
| 2.69E-02 | -1.59 | EPS8L1 | EPS8 like 1 |
| 1.71E-03 | -1.59 | TPGS1 | Tubulin polyglutamylase complex subunit 1 |
| 1.70E-03 | -1.59 | CADM3 | Cell adhesion molecule 3 |
| 3.34E-02 | -1.59 | AL138963.3 | *Antisense RNA* |
| 3.88E-03 | -1.58 | HES4 | Hes family bHLH transcription factor 4 |
| 4.02E-04 | -1.58 | KCNIP1 | Potassium voltage-gated channel interacting protein 1 |
| 3.85E-03 | -1.58 | OMG | Oligodendrocyte myelin glycoprotein |
| 1.11E-03 | -1.58 | DPY19L2P3 | DPY19L2 pseudogene 3 |
| 5.77E-03 | -1.58 | SOX21-AS1 | SOX21 antisense RNA 1 (head to head) |
| 5.62E-03 | -1.58 | CCDC85B | Coiled-coil domain containing 85B |
| 2.66E-02 | -1.57 | AL391261.2 | *Long intergenic non-protein coding RNA* |
| 7.69E-03 | -1.57 | RPS15P4 | Ribosomal Protein S15 Pseudogene 4 |
| 3.69E-03 | -1.57 | BGN | Biglycan |
| 1.24E-02 | -1.57 | RAMP2 | Receptor activity modifying protein 2 |
| 4.28E-02 | -1.56 | TBX6 | T-box 6 |
| 1.10E-02 | -1.56 | KCNMB2 | Potassium calcium-activated channel subfamily M regulatory beta subunit 2 |
| 7.45E-03 | -1.56 | JSRP1 | Junctional sarcoplasmic reticulum protein 1 |
| 1.18E-03 | -1.55 | STUM | Stum, mechanosensory transduction mediator homolog |
| 4.73E-03 | -1.55 | AC084824.4 | *Sense intronic RNA* |
| 4.16E-03 | -1.55 | KRT4 | Keratin 4 |
| 1.93E-03 | -1.55 | AL391650.1 | *Protein coding gene* |
| 4.84E-03 | -1.54 | CLEC11A | C-type lectin domain family 11 member A |

| | | | |
|---|---|---|---|
| 1.61E-02 | -1.54 | CR2 | Complement component 3d receptor 2 |
| 6.37E-03 | -1.54 | MIR503HG | MIR503 host gene |
| 5.83E-03 | -1.54 | FAM69C | Family with sequence similarity 69 member C |
| 2.60E-02 | -1.53 | FAM19A4 | Family with sequence similarity 19 member A4, C-C motif chemokine like |
| 9.35E-03 | -1.53 | LINC01024 | *Long intergenic non-protein coding RNA 1024* |
| 1.58E-02 | -1.52 | AHRR | Aryl-hydrocarbon receptor repressor |
| 4.63E-03 | -1.52 | CFD | Complement factor D |
| 2.40E-03 | -1.52 | SLC12A5 | Solute carrier family 12 member 5 |
| 2.60E-03 | -1.52 | COL2A1 | Collagen type II alpha 1 chain |
| 1.77E-02 | -1.51 | LINC00862 | *Long intergenic non-protein coding RNA 862* |
| 6.00E-04 | -1.51 | ZNF837 | Zinc finger protein 837 |
| 3.26E-02 | -1.51 | SOCS1 | Suppressor of cytokine signaling 1 |
| 4.10E-02 | -1.51 | HTR1D | 5-hydroxytryptamine receptor 1D |
| 1.47E-03 | -1.51 | ERICH6-AS1 | ERICH6 antisense RNA 1 |
| 3.29E-03 | -1.51 | FAM81B | Family with sequence similarity 81 member B |
| 2.60E-02 | -1.51 | AC016723.1 | *Antisense RNA* |
| 2.32E-02 | -1.51 | STX17-AS1 | STX17 antisense RNA 1 |
| 2.71E-03 | -1.50 | EVA1B | Eva-1 homolog B |
| 8.12E-03 | -1.50 | CTU1 | Cytosolic thiouridylase subunit 1 |
| 2.56E-02 | -1.50 | AL117332.1 | *Antisense RNA* |
| 3.63E-02 | -1.50 | ID2-AS1 | ID2 antisense RNA 1 (head to head) |

Genes are classified according to the fold change with a *P*-value and fold-change cut-off of $P < 0.05$ and FC < -1.5, respectively. FC, fold-change.

**Supplementary Table S4** RNA-Seq transcriptomic analysis predicted cortisol up-regulated genes. Cortisol-treated samples compared to untreated control samples.

**Cortisol $10^{-7}$ M** **Up-regulated genes**

| *P*-value | FC | Gene name | Description |
|---|---|---|---|
| 1.81E-152 | **6.48** | **SCNN1A** | Sodium channel epithelial 1 alpha subunit |
| 1.88E-202 | **5.36** | **FKBP5** | FK506 binding protein 5 |
| 4.71E-32 | **4.15** | **HVCN1** | Hydrogen voltage gated channel 1 |
| 5.41E-49 | **3.13** | **POU5F1** | POU class 5 homeobox 1 |
| 1.10E-07 | **3.06** | **ADH1B** | Alcohol dehydrogenase 1B (class I), beta polypeptide |
| 5.32E-77 | **3.00** | **MIR6883 / PER1** | MicroRNA 6883 / Period circadian clock 1 |
| 4.19E-40 | **2.97** | **HIF3A** | Hypoxia inducible factor 3 alpha subunit |
| 1.44E-82 | **2.68** | **ACSS1** | Acyl-CoA synthetase short-chain family member 1 |
| 2.79E-12 | **2.66** | **SLC28A3** | Solute carrier family 28 member 3 |
| 5.28E-54 | **2.62** | **PTGER2** | Prostaglandin E receptor 2 |
| 1.93E-36 | **2.56** | **MAP7D2** | MAP7 domain containing 2 |
| 1.29E-22 | **2.53** | **SERPINA3** | Serpin family A member 3 |
| 4.21E-20 | **2.38** | **EPHA8** | EPH receptor A8 |
| 2.99E-22 | **2.31** | **TLE2** | Transducin like enhancer of split 2 |
| 8.46E-132 | **2.24** | **ST6GALNAC2** | ST6 N-acetylgalactosaminide alpha-2, 6-sialyltransferase 2 |
| 1.57E-03 | **2.18** | **APCDD1L** | APC down-regulated 1 like |
| 8.35E-04 | **2.15** | **MIR7-1** | MicroRNA 7-1 |
| 4.57E-51 | **2.12** | **REPS2** | RALBP1 associated Eps domain containing 2 |
| 7.08E-08 | **2.06** | **CES4A** | Carboxylesterase 4A |
| 3.41E-95 | **2.03** | **SLC13A4** | Solute carrier family 13 member 4 |
| 9.89E-34 | **2.01** | **PRLR** | Prolactin receptor |
| 1.82E-08 | **1.96** | **CDH20** | Cadherin 20 |
| 6.25E-39 | **1.96** | **SLC16A12** | Solute carrier family 16 member 12 |
| 1.16E-24 | **1.95** | **FAM196B** | Family with sequence similarity 196 member B |
| 3.65E-09 | **1.94** | **MAOA** | Monoamine oxidase A |
| 4.56E-05 | **1.93** | **STAC2** | SH3 and cysteine rich domain 2 |
| 9.77E-16 | **1.92** | **SYTL3** | Synaptotagmin like 3 |
| 2.90E-22 | **1.92** | **TRMT9B** | TRNA Methyltransferase 9B |
| 3.25E-09 | **1.92** | **H19 / MIR675** | H19, imprinted maternally expressed transcript / MicroRNA 675 |
| 1.94E-34 | **1.91** | **HPD** | 4-hydroxyphenylpyruvate dioxygenase |
| 1.13E-22 | **1.88** | **SH3PXD2B** | SH3 and PX domains 2B |
| 7.99E-71 | **1.87** | **TCEAL4** | Transcription elongation factor A like 4 |
| 4.28E-21 | **1.85** | **RND2** | Rho family GTPase 2 |
| 3.95E-03 | **1.84** | **DUSP5** | Dual specificity phosphatase 5 |
| 6.57E-17 | **1.83** | **TMEM72** | Transmembrane protein 72 |
| 2.59E-107 | **1.83** | **TSC22D3** | TSC22 domain family member 3 |
| 2.93E-03 | **1.82** | **DUSP6** | Dual specificity phosphatase 6 |
| 5.67E-15 | **1.81** | **CXCR4** | C-X-C motif chemokine receptor 4 |
| 2.08E-50 | **1.80** | **IQSEC3** | IQ motif and Sec7 domain 3 |
| 2.86E-13 | **1.79** | **TMEM229B** | Transmembrane protein 229B |
| 4.35E-45 | **1.79** | **RPS6KA2** | Ribosomal protein S6 kinase A2 |
| 3.40E-40 | **1.77** | **KLF15** | Kruppel like factor 15 |
| 5.28E-12 | **1.77** | **METTL7A** | Methyltransferase like 7A |
| 5.77E-04 | **1.76** | **DKK1** | Dickkopf WNT signaling pathway inhibitor 1 |

| | | | |
|---|---|---|---|
| 3.59E-22 | **1.75** | **AIM1** | Absent in melanoma 1 |
| 1.03E-30 | **1.75** | **MIOS** | Meiosis regulator for oocyte development |
| 3.29E-22 | **1.75** | **ABCC5** | ATP binding cassette subfamily C member 5 |
| 4.18E-05 | **1.75** | **PAPLN** | Papilin, proteoglycan like sulfated glycoprotein |
| 1.71E-40 | **1.73** | **USP53** | Ubiquitin specific peptidase 53 |
| 1.01E-05 | **1.73** | **GRAMD1C** | GRAM domain containing 1C |
| 1.10E-11 | **1.73** | **ALDH1L1** | Aldehyde dehydrogenase 1 family member L1 |
| 1.02E-12 | **1.72** | **ST3GAL5** | ST3 beta-galactoside alpha-2, 3-sialyltransferase 5 |
| 4.31E-07 | **1.72** | **KBTBD8** | Kelch repeat and BTB domain containing 8 |
| 5.22E-05 | **1.71** | **KRT5** | Keratin 5 |
| 2.01E-40 | **1.70** | **SERPINF2** | Serpin family F member 2 |
| 8.53E-12 | **1.70** | **CP** | Ceruloplasmin |
| 1.36E-18 | **1.69** | **SOD2** | Superoxide dismutase 2, mitochondrial |
| 6.93E-91 | **1.69** | **PRODH** | Proline dehydrogenase 1 |
| 4.73E-11 | **1.69** | **ABCA4** | ATP binding cassette subfamily A member 4 |
| 2.04E-04 | **1.68** | **SCGB1D2** | Secretoglobin family 1D member 2 |
| 3.04E-03 | **1.66** | **S100A2** | S100 calcium binding protein A2 |
| 2.10E-18 | **1.64** | **TGFBR2** | Transforming growth factor beta receptor 2 |
| 2.21E-09 | **1.64** | **BICDL1** | BICD family like cargo adaptor 1 |
| 3.83E-80 | **1.64** | **CRYZ** | Crystallin zeta |
| 1.05E-14 | **1.63** | **MT1X** | Metallothionein 1X |
| 1.57E-33 | **1.63** | **IQGAP2** | IQ motif containing GTPase activating protein 2 |
| 1.47E-16 | **1.62** | **MCTP2** | Multiple C2 and transmembrane domain containing 2 |
| 3.75E-04 | **1.61** | **WNT11** | Wnt family member 11 |
| 1.69E-03 | **1.61** | **LINC01088** | *Long intergenic non-protein coding RNA 1088* |
| 2.05E-44 | **1.61** | **PCP4** | Purkinje cell protein 4 |
| 4.79E-07 | **1.60** | **SLC1A7** | Solute carrier family 1 member 7 |
| 3.19E-14 | **1.60** | **PLIN2** | Perilipin 2 |
| 8.18E-52 | **1.60** | **ITGB4** | Integrin subunit beta 4 |
| 4.87E-12 | **1.60** | **FHIT** | Fragile histidine triad |
| 1.75E-05 | **1.58** | **ATP6V0A4** | ATPase $H^+$ transporting V0 subunit a4 |
| 1.58E-04 | **1.58** | **ZFP36** | ZFP36 ring finger protein |
| 2.75E-04 | **1.58** | **FAM167A-AS1** | FAM167A antisense RNA 1 |
| 3.72E-02 | **1.58** | **MIR641** | MicroRNA 641 |
| 2.58E-42 | **1.58** | **SLC2A12** | Solute carrier family 2 member 12 |
| 2.97E-14 | **1.57** | **CEBPD** | CCAAT/enhancer binding protein delta |
| 2.25E-03 | **1.57** | **NDRG4** | NDRG family member 4 |
| 4.65E-09 | **1.57** | **USP2** | Ubiquitin specific peptidase 2 |
| 6.84E-17 | **1.57** | **KCNA5** | Potassium voltage-gated channel subfamily A member 5 |
| 8.43E-35 | **1.57** | **SGK1** | Serum/glucocorticoid regulated kinase 1 |
| 3.19E-28 | **1.57** | **INPP5K** | Inositol polyphosphate-5-phosphatase K |
| 1.77E-30 | **1.57** | **SLC16A10** | Solute carrier family 16 member 10 |
| 2.80E-20 | **1.56** | **PPM1K** | Protein phosphatase, $Mg^{2+}$/$Mn^{2+}$ dependent 1K |
| 3.76E-07 | **1.56** | **XYLB** | Xylulokinase |
| 1.45E-25 | **1.55** | **CHST11** | Carbohydrate sulfotransferase 11 |
| 2.78E-03 | **1.54** | **EEPD1** | Endonuclease/exonuclease/phosphatase family domain containing 1 |
| 2.87E-03 | **1.53** | **CYTIP** | Cytohesin 1 interacting protein |
| 3.65E-13 | **1.53** | **AADACL4** | Arylacetamide deacetylase like 4 |
| 9.96E-16 | **1.53** | **SLCO4A1** | Solute carrier organic anion transporter family member 4A1 |
| 3.59E-05 | **1.53** | **ADAMTSL1** | ADAMTS like 1 |
| 4.94E-18 | **1.53** | **ADAMTSL4** | ADAMTS like 4 |
| 1.72E-07 | **1.52** | **LINC00330** | *Long intergenic non-protein coding RNA 330* |
| 1.61E-02 | **1.51** | **B4GALNT1** | Beta-1, 4-N-acetyl-galactosaminyltransferase 1 |
| 1.09E-05 | **1.51** | **CD58** | CD58 molecule |
| 3.95E-06 | **1.50** | **MAFF** | MAF bZIP transcription factor F |

Genes are classified according to the fold change with a *P*-value and fold-change cut-off of $P < 0.05$ and $FC > 1.5$, respectively. FC, fold-change.

**Supplementary Table S5** RNA-Seq transcriptomic analysis predicted cortisol down-regulated genes. Cortisol-treated samples compared to untreated control samples.

**Cortisol $10^{-7}$ M** **Down-regulated genes**

| *P*-value | FC | Gene name | Description |
|---|---|---|---|
| 1.64E-04 | **-2.40** | **TGM2** | Transglutaminase 2 |
| 9.25E-04 | **-2.35** | **KRT81** | Keratin 81 |
| 8.37E-03 | **-2.26** | **CNN1** | Calponin 1 |
| 5.39E-05 | **-2.25** | **SPP1** | Secreted phosphoprotein 1 |
| 3.59E-07 | **-2.24** | **ITGA11** | Integrin subunit alpha 11 |
| 1.63E-06 | **-2.08** | **AMTN** | Amelotin |
| 3.31E-03 | **-2.07** | **INHBA** | Inhibin beta A subunit |
| 2.95E-05 | **-2.02** | **OMG** | Oligodendrocyte myelin glycoprotein |
| 6.84E-04 | **-1.95** | **C1orf61 / MIR9-1** | *Chromosome 1 open reading frame 61* / MicroRNA 9-1 |
| 1.81E-05 | **-1.95** | **TK1** | Thymidine kinase 1 |
| 1.52E-18 | **-1.85** | **IGF1** | Insulin like growth factor 1 |
| 8.35E-04 | **-1.83** | **CYS1** | Cystin 1 |
| 2.47E-08 | **-1.81** | **DTL** | Denticleless E3 ubiquitin protein ligase homolog |
| 1.02E-02 | **-1.79** | **ANKRD1** | Ankyrin repeat domain 1 |
| 2.40E-03 | **-1.78** | **SFRP2** | Secreted frizzled related protein 2 |
| 8.82E-05 | **-1.74** | **KCND2** | Potassium voltage-gated channel subfamily D member 2 |
| 2.48E-04 | **-1.71** | **CDCA7** | Cell division cycle associated 7 |
| 8.96E-05 | **-1.70** | **GJD2** | Gap junction protein delta 2 |
| 4.81E-05 | **-1.69** | **DEPDC1B** | DEP domain containing 1B |
| 3.85E-03 | **-1.69** | **SST** | Somatostatin |
| 3.35E-03 | **-1.68** | **COL1A1** | Collagen type I alpha 1 chain |
| 1.58E-02 | **-1.66** | **PAPPA** | Pappalysin 1 |
| 1.77E-03 | **-1.64** | **IGFBP3** | Insulin like growth factor binding protein 3 |
| 1.13E-04 | **-1.63** | **SMOC1** | SPARC related modular calcium binding 1 |
| 4.88E-04 | **-1.61** | **NTRK3** | Neurotrophic receptor tyrosine kinase 3 |
| 3.67E-03 | **-1.61** | **ZNF367** | Zinc finger protein 367 |
| 1.18E-10 | **-1.61** | **ROBO2** | Roundabout guidance receptor 2 |
| 3.80E-05 | **-1.60** | **FAM111B** | Family with sequence similarity 111 member B |
| 1.79E-04 | **-1.60** | **ASF1B** | Anti-silencing function 1B histone chaperone |
| 6.15E-04 | **-1.58** | **TCF19** | Transcription factor 19 |
| 1.44E-03 | **-1.58** | **CDCA5** | Cell division cycle associated 5 |
| 3.00E-02 | **-1.58** | **CDC20B** | Cell division cycle 20B |
| 8.91E-12 | **-1.57** | **ADAMTS1** | ADAM metallopeptidase with thrombospondin type 1 motif 1 |
| 1.46E-08 | **-1.55** | **RRM2** | Ribonucleotide reductase regulatory subunit M2 |
| 3.77E-02 | **-1.55** | **NACAD** | NAC alpha domain containing |
| 1.50E-02 | **-1.55** | **STK32B** | Serine/threonine kinase 32B |
| 8.99E-05 | **-1.54** | **NDST3** | N-deacetylase and N-sulfotransferase 3 |
| 3.30E-03 | **-1.54** | **TTK** | TTK protein kinase |
| 8.85E-03 | **-1.54** | **CENPK** | Centromere protein K |
| 1.36E-08 | **-1.52** | **IGSF9** | Immunoglobulin superfamily member 9 |
| 2.11E-03 | **-1.52** | **SHCBP1** | SHC binding and spindle associated 1 |

Genes are classified according to the fold change with a *P*-value and fold-change cut-off of $P < 0.05$ and FC < -1.5, respectively. FC, fold-change.

**Supplementary Table S6** RNA-Seq transcriptomic analysis predicted cortisol + RU-486 up-regulated genes. Cortisol + RU-486-treated samples compared to untreated control samples.

**Cortisol $10^{-7}$ M + RU-486 $10^{-5}$ M** **Up-regulated genes**

| *P*-value | FC | Gene name | Description |
|---|---|---|---|
| 8.09E-17 | **16.20** | **AKR1C1** | Aldo-keto reductase family 1 member C1 |
| 9.08E-12 | **13.96** | **HSPA6** | Heat shock protein family A (Hsp70) member 6 |
| 1.08E-12 | **7.56** | **AKR1B10** | Aldo-keto reductase family 1 member B10 |
| 2.25E-08 | **6.06** | **MMP1** | Matrix metallopeptidase 1 |
| 6.09E-05 | **5.02** | **AGXT** | Alanine-glyoxylate aminotransferase |
| 5.65E-14 | **4.82** | **DUSP5** | Dual specificity phosphatase 5 |
| 3.17E-08 | **3.81** | **FOSL1** | FOS like 1, AP-1 transcription factor subunit |
| 2.08E-31 | **3.56** | **ANGPTL4** | Angiopoietin like 4 |
| 1.92E-11 | **3.54** | **SRPX2** | Sushi repeat containing protein, X-linked 2 |
| 3.12E-08 | **3.40** | **IL7R** | Interleukin 7 receptor |
| 5.36E-54 | **2.96** | **HMGCS2** | 3-hydroxy-3-methylglutaryl-CoA synthase 2 |
| 1.83E-07 | **2.85** | **DUSP6** | Dual specificity phosphatase 6 |
| 3.52E-64 | **2.83** | **PLIN2** | Perilipin 2 |
| 1.94E-12 | **2.71** | **NDRG4** | NDRG family member 4 |
| 6.14E-08 | **2.60** | **ABCC3** | ATP binding cassette subfamily C member 3 |
| 3.25E-08 | **2.47** | **DKK1** | Dickkopf WNT signaling pathway inhibitor 1 |
| 3.94E-08 | **2.46** | **B4GALNT1** | Beta-1, 4-N-acetyl-galactosaminyltransferase 1 |
| 1.15E-10 | **2.38** | **PLAUR** | Plasminogen activator, urokinase receptor |
| 1.05E-05 | **2.30** | **TM4SF1** | Transmembrane 4 L six family member 1 |
| 1.56E-06 | **2.28** | **CA12** | Carbonic anhydrase 12 |
| 3.93E-26 | **2.21** | **SCNN1A** | Sodium channel epithelial 1 alpha subunit |
| 1.15E-10 | **2.21** | **PDK4** | Pyruvate dehydrogenase kinase 4 |
| 1.95E-07 | **2.17** | **CLMP** | CXADR like membrane protein |
| 3.89E-05 | **2.14** | **PTX3** | Pentraxin 3 |
| 1.62E-05 | **2.12** | **SLC24A4** | Solute carrier family 24 member 4 |
| 3.52E-09 | **2.12** | **SERPINE2** | Serpin family E member 2 |
| 6.41E-05 | **2.08** | **MARCH4** | Membrane associated ring-CH-type finger 4 |
| 2.47E-14 | **2.05** | **RAB27B** | RAB27B, member RAS oncogene family |
| 3.18E-08 | **2.03** | **FGF12** | Fibroblast growth factor 12 |
| 5.61E-34 | **2.02** | **FKBP5** | FK506 binding protein 5 |
| 4.98E-04 | **1.99** | **PRCD** | Progressive rod-cone degeneration |
| 7.60E-08 | **1.97** | **HVCN1** | Hydrogen voltage gated channel 1 |
| 7.43E-07 | **1.97** | **HMGA1 / MIR6835** | High mobility group AT-hook 1 / MicroRNA 6835 |
| 7.58E-03 | **1.96** | **CALB2** | Calbindin 2 |
| 1.63E-26 | **1.95** | **PTGER2** | Prostaglandin E receptor 2 |
| 2.60E-06 | **1.94** | **EFCAB13 / ITGB3** | EF-hand calcium binding domain 13 / Integrin subunit beta 3 |
| 1.18E-08 | **1.93** | **ANXA1** | Annexin A1 |
| 6.93E-10 | **1.92** | **KCNMA1** | Potassium calcium-activated channel subfamily M alpha 1 |
| 4.91E-06 | **1.91** | **METTL7B** | Methyltransferase like 7B |
| 7.22E-08 | **1.90** | **DLGAP1-AS2** | DLGAP1 antisense RNA 2 |
| 4.09E-05 | **1.89** | **ISM2** | Isthmin 2 |

| | | | |
|---|---|---|---|
| 1.08E-07 | **1.88** | **PHLDA1** | Pleckstrin homology like domain family A member 1 |
| 3.92E-04 | **1.88** | **ISG15** | ISG15 ubiquitin-like modifier |
| 4.02E-11 | **1.88** | **MT2A** | Metallothionein 2A |
| 8.34E-13 | **1.87** | **MAFF** | MAF bZIP transcription factor F |
| 6.79E-11 | **1.84** | **SLC1A7** | Solute carrier family 1 member 7 |
| 3.66E-12 | **1.84** | **CDCP1** | CUB domain containing protein 1 |
| 3.17E-06 | **1.82** | **ECM1** | Extracellular matrix protein 1 |
| 1.24E-20 | **1.80** | **DBI** | Diazepam binding inhibitor, acyl-CoA binding protein |
| 8.51E-07 | **1.79** | **MME** | Membrane metallo-endopeptidase |
| 3.61E-07 | **1.77** | **SPRY2** | Sprouty RTK signaling antagonist 2 |
| 5.04E-12 | **1.76** | **LAMC2** | Laminin subunit gamma 2 |
| 5.49E-15 | **1.76** | **TMEM72** | Transmembrane protein 72 |
| 2.88E-09 | **1.76** | **HILPDA** | Hypoxia inducible lipid droplet associated |
| 3.15E-05 | **1.76** | **IL6** | Interleukin 6 |
| 1.87E-13 | **1.75** | **ST3GAL5** | ST3 beta-galactoside alpha-2, 3-sialyltransferase 5 |
| 6.16E-04 | **1.74** | **ACSL5** | Acyl-CoA synthetase long-chain family member 5 |
| 3.09E-17 | **1.74** | **RSAD2** | Radical S-adenosyl methionine domain containing 2 |
| 1.43E-08 | **1.74** | **OSGIN1** | Oxidative stress induced growth inhibitor 1 |
| 2.72E-05 | **1.73** | **PTPRN** | Protein tyrosine phosphatase, receptor type N |
| 2.71E-02 | **1.73** | **VMO1** | Vitelline membrane outer layer 1 homolog |
| 1.76E-03 | **1.72** | **SLC3A1** | Solute carrier family 3 member 1 |
| 9.60E-09 | **1.72** | **NQO1** | NAD(P)H quinone dehydrogenase 1 |
| 2.94E-06 | **1.71** | **TRIB3** | Tribbles pseudokinase 3 |
| 1.66E-03 | **1.71** | **S100A2** | S100 calcium binding protein A2 |
| 2.37E-02 | **1.69** | **MIR7-1** | MicroRNA 7-1 |
| 7.71E-06 | **1.69** | **TESC** | Tescalcin |
| 3.99E-52 | **1.67** | **CPT1A** | Carnitine palmitoyltransferase 1A |
| 1.62E-06 | **1.67** | **KBTBD8** | Kelch repeat and BTB domain containing 8 |
| 2.28E-02 | **1.67** | **ACER2** | Alkaline ceramidase 2 |
| 1.58E-18 | **1.66** | **PRLR** | Prolactin receptor |
| 3.29E-03 | **1.66** | **WSCD2** | WSC domain containing 2 |
| 2.09E-28 | **1.66** | **RMI1** | RecQ mediated genome instability 1 |
| 1.29E-03 | **1.65** | **ANO4** | Anoctamin 4 |
| 3.38E-15 | **1.65** | **FRMPD3** | FERM and PDZ domain containing 3 |
| 1.05E-10 | **1.64** | **CXCR4** | C-X-C motif chemokine receptor 4 |
| 6.53E-04 | **1.64** | **DRGX** | Dorsal root ganglia homeobox |
| 1.18E-05 | **1.63** | **SLC22A6** | Solute carrier family 22 member 6 |
| 3.84E-20 | **1.63** | **TMEM158** | Transmembrane protein 158 (gene/pseudogene) |
| 3.90E-07 | **1.62** | **SERPINA3** | Serpin family A member 3 |
| 2.90E-15 | **1.62** | **IL33** | Interleukin 33 |
| 3.20E-07 | **1.61** | **SLC9A9** | Solute carrier family 9 member A9 |
| 1.21E-06 | **1.61** | **PMAIP1** | Phorbol-12-myristate-13-acetate-induced protein 1 |
| 1.87E-02 | **1.60** | **RAET1G** | Retinoic acid early transcript 1G |
| 1.52E-06 | **1.60** | **BSPRY** | B-box and SPRY domain containing |
| 2.00E-05 | **1.59** | **IFITM1** | Interferon induced transmembrane protein 1 |
| 8.30E-04 | **1.59** | **TNFSF18** | Tumor necrosis factor superfamily member 18 |
| 5.00E-03 | **1.59** | **BTBD11** | BTB domain containing 11 |
| 3.47E-18 | **1.57** | **MGST1** | Microsomal glutathione S-transferase 1 |
| 6.71E-10 | **1.57** | **RLBP1** | Retinaldehyde binding protein 1 |
| 1.63E-10 | **1.57** | **ATP1B2** | ATPase $Na^+/K^+$ transporting subunit beta 2 |
| 1.65E-04 | **1.57** | **UPP1** | Uridine phosphorylase 1 |
| 9.15E-14 | **1.56** | **C1QTNF1** | C1q and tumor necrosis factor related protein 1 |
| 3.68E-05 | **1.55** | **CYP1B1** | Cytochrome P450 family 1 subfamily B member 1 |
| 2.99E-38 | **1.55** | **PCP4** | Purkinje cell protein 4 |
| 1.34E-26 | **1.55** | **SLC18A3** | Solute carrier family 18 member A3 |
| 1.10E-07 | **1.55** | **BICDL1** | BICD family like cargo adaptor 1 |
| 2.16E-02 | **1.55** | **FAM196A** | Family with sequence similarity 196 member A |

| | | | |
|---|---|---|---|
| 4.12E-10 | **1.55** | **ETV5** | ETS variant 5 |
| 4.50E-10 | **1.55** | **LAYN** | Layilin |
| 6.36E-26 | **1.54** | **RPS6KA2** | Ribosomal protein S6 kinase A2 |
| 5.36E-08 | **1.54** | **ABCA4** | ATP binding cassette subfamily A member 4 |
| 5.01E-04 | **1.54** | **RIN1** | Ras and Rab interactor 1 |
| 2.47E-03 | **1.54** | **SERPINE1** | Serpin family E member 1 |
| 2.73E-09 | **1.54** | **CD44** | CD44 molecule (Indian blood group) |
| 5.57E-13 | **1.54** | **GNG2** | G protein subunit gamma 2 |
| 6.35E-11 | **1.54** | **FAM196B** | Family with sequence similarity 196 member B |
| 2.85E-08 | **1.53** | **SPARCL1** | SPARC like 1 |
| 4.39E-10 | **1.53** | **OSMR** | Oncostatin M receptor |
| 3.15E-15 | **1.53** | **KCNA5** | Potassium voltage-gated channel subfamily A member 5 |
| 4.76E-05 | **1.53** | **SLC16A6** | Solute carrier family 16 member 6 |
| 2.93E-04 | **1.53** | **C10orf53 / CHAT** | *Chromosome 10 open reading frame 53* / Choline O-acetyltransferase |
| 5.28E-09 | **1.52** | **MGLL** | Monoglyceride lipase |
| 2.07E-12 | **1.52** | **SOD2** | Superoxide dismutase 2, mitochondrial |
| 1.88E-02 | **1.52** | **TSPAN1** | Tetraspanin 1 |
| 1.10E-24 | **1.52** | **IQGAP2** | IQ motif containing GTPase activating protein 2 |
| 6.66E-14 | **1.52** | **SLC7A11** | Solute carrier family 7 member 11 |
| 3.03E-10 | **1.51** | **RND2** | Rho family GTPase 2 |
| 1.76E-11 | **1.51** | **MIR6883 / PER1** | MicroRNA 6883 / Period circadian clock 1 |
| 2.83E-07 | **1.51** | **GABBR2** | Gamma-aminobutyric acid type B receptor subunit 2 |
| 1.04E-03 | **1.51** | **UBASH3B** | Ubiquitin associated and SH3 domain containing B |
| 2.43E-05 | **1.50** | **EPHA8** | EPH receptor A8 |
| 2.26E-22 | **1.50** | **CHST11** | Carbohydrate sulfotransferase 11 |

Genes are classified according to the fold change with a *P*-value and fold-change cut-off of $P < 0.05$ and FC > 1.5, respectively. FC, fold-change.

**Supplementary Table S7** RNA-Seq transcriptomic analysis predicted cortisol + RU-486 down-regulated genes. Cortisol + RU-486-treated samples compared to untreated control samples.

## Cortisol $10^{-7}$ M + RU-486 $10^{-5}$ M — Down-regulated genes

| *P*-value | FC | Gene name | Description |
|---|---|---|---|
| 6.94E-06 | **-4.32** | **CNN1** | Calponin 1 |
| 6.10E-20 | **-3.92** | **GJD2** | Gap junction protein delta 2 |
| 8.79E-16 | **-3.24** | **C14orf180** | *Chromosome 14 open reading frame 180* |
| 6.48E-06 | **-2.86** | **TGM2** | Transglutaminase 2 |
| 1.54E-09 | **-2.80** | **OMG** | Oligodendrocyte myelin glycoprotein |
| 1.32E-10 | **-2.55** | **RPL3L** | Ribosomal protein L3 like |
| 3.67E-12 | **-2.41** | **KCNAB1** | Potassium voltage-gated channel subfamily A member regulatory beta subunit 1 |
| 1.93E-08 | **-2.37** | **AMTN** | Amelotin |
| 7.01E-33 | **-2.35** | **TAGLN** | Transgelin |
| 1.45E-08 | **-2.34** | **ELF3** | E74 like ETS transcription factor 3 |
| 6.16E-29 | **-2.27** | **GAS2L1P2** | Growth arrest specific 2 like 1 pseudogene 2 |
| 3.59E-08 | **-2.24** | **ADAMTS5** | ADAM metallopeptidase with thrombospondin type 1 motif 5 |
| 5.48E-07 | **-2.21** | **MGP** | Matrix Gla protein |
| 3.49E-07 | **-2.20** | **HHATL** | Hedgehog acyltransferase-like |
| 7.42E-07 | **-2.19** | **ITGA11** | Integrin subunit alpha 11 |
| 4.81E-09 | **-2.16** | **FGD3** | FYVE, RhoGEF and PH domain containing 3 |
| 4.92E-19 | **-2.13** | **ACTA2** | Actin, alpha 2, smooth muscle, aorta |
| 5.82E-11 | **-2.09** | **TNNT2** | Troponin T2, cardiac type |
| 4.58E-03 | **-2.06** | **KRT81** | Keratin 81 |
| 5.94E-08 | **-2.04** | **KIF20A** | Kinesin family member 20A |
| 5.05E-16 | **-2.02** | **CARNS1** | Carnosine synthase 1 |
| 4.15E-07 | **-2.01** | **GFRA2** | GDNF family receptor alpha 2 |
| 2.81E-06 | **-2.01** | **GPR37L1** | G protein-coupled receptor 37 like 1 |
| 8.82E-05 | **-2.00** | **HAPLN3** | Hyaluronan and proteoglycan link protein 3 |
| 1.42E-07 | **-1.97** | **SOX8** | SRY-box 8 |
| 1.21E-06 | **-1.97** | **NTRK3** | Neurotrophic receptor tyrosine kinase 3 |
| 5.82E-03 | **-1.96** | **SEMA3B-AS1** | SEMA3B antisense RNA 1 (head to head) |
| 6.23E-03 | **-1.96** | **INHBA** | Inhibin beta A subunit |
| 1.99E-11 | **-1.94** | **CYP2W1** | Cytochrome P450 family 2 subfamily W member 1 |
| 1.03E-05 | **-1.93** | **RUNX1T1** | RUNX1 translocation partner 1 |
| 1.30E-07 | **-1.92** | **CCNA1** | Cyclin A1 |
| 5.84E-19 | **-1.91** | **FGFR3** | Fibroblast growth factor receptor 3 |
| 2.49E-11 | **-1.91** | **AP1M2** | Adaptor related protein complex 1 mu 2 subunit |
| 5.27E-06 | **-1.89** | **CYP19A1** | Cytochrome P450 family 19 subfamily A member 1 |
| 1.19E-23 | **-1.88** | **ERF** | ETS2 repressor factor |
| 3.09E-07 | **-1.88** | **PPP1R14A** | Protein phosphatase 1 regulatory inhibitor subunit 14A |
| 5.47E-07 | **-1.85** | **ATP6V1B1** | ATPase $H^+$ transporting V1 subunit B1 |
| 1.74E-15 | **-1.85** | **OXTR** | Oxytocin receptor |
| 6.52E-03 | **-1.85** | **ANKRD1** | Ankyrin repeat domain 1 |
| 3.55E-03 | **-1.85** | **CDC20B** | Cell division cycle 20B |
| 4.51E-03 | **-1.84** | **NACAD** | NAC alpha domain containing |
| 4.74E-07 | **-1.84** | **SPC24** | SPC24, NDC80 kinetochore complex component |
| 2.53E-08 | **-1.84** | **RHBDL2** | Rhomboid like 2 |
| 1.67E-10 | **-1.81** | **C5orf46** | Chromosome 5 open reading frame 46 |
| 2.77E-11 | **-1.81** | **BUB1B /** | BUB1 mitotic checkpoint serine/threonine kinase B / |

| | | | |
|---|---|---|---|
| | | **PAK6** | p21 (RAC1) activated kinase 6 |
| 1.02E-13 | **-1.80** | **C10orf54** | *Chromosome 10 open reading frame 54* |
| 7.86E-08 | **-1.79** | **GAS6-AS1** | GAS6 antisense RNA 1 |
| 4.00E-08 | **-1.79** | **DLGAP5** | DLG associated protein 5 |
| 2.51E-03 | **-1.77** | **NEK2** | NIMA related kinase 2 |
| 9.27E-23 | **-1.76** | **MMP11** | Matrix metallopeptidase 11 |
| 3.50E-04 | **-1.75** | **SYT10** | Synaptotagmin 10 |
| 3.78E-06 | **-1.74** | **RGS9** | Regulator of G-protein signaling 9 |
| 4.60E-28 | **-1.74** | **PRSS12** | Protease, serine 12 |
| 2.06E-06 | **-1.74** | **WNT2B** | Wnt family member 2B |
| 7.83E-04 | **-1.74** | **C21orf58** | *Chromosome 21 open reading frame 58* |
| 2.64E-02 | **-1.73** | **MOG** | Myelin oligodendrocyte glycoprotein |
| 5.86E-04 | **-1.73** | **TPPP3** | Tubulin polymerization promoting protein family member 3 |
| 2.44E-04 | **-1.72** | **PLK1** | Polo like kinase 1 |
| 6.81E-05 | **-1.72** | **LUM** | Lumican |
| 1.61E-07 | **-1.72** | **CCNA2** | Cyclin A2 |
| 6.57E-05 | **-1.71** | **KIF4A** | Kinesin family member 4A |
| 2.54E-11 | **-1.69** | **MFSD2A** | Major facilitator superfamily domain containing 2A |
| 2.68E-06 | **-1.69** | **NDST3** | N-deacetylase and N-sulfotransferase 3 |
| 8.82E-13 | **-1.69** | **BMF** | Bcl2 modifying factor |
| 6.37E-05 | **-1.69** | **MKI67** | Marker of proliferation Ki-67 |
| 3.07E-07 | **-1.69** | **VGLL3** | Vestigial like family member 3 |
| 4.34E-13 | **-1.68** | **INHBB** | Inhibin beta B subunit |
| 1.89E-12 | **-1.68** | **ERC2** | ELKS/RAB6-interacting/CAST family member 2 |
| 3.78E-09 | **-1.68** | **HSPB7** | Heat shock protein family B (small) member 7 |
| 1.31E-04 | **-1.67** | **CYP2S1** | Cytochrome P450 family 2 subfamily S member 1 |
| 1.11E-04 | **-1.67** | **ADAMTS15** | ADAM metallopeptidase with thrombospondin type 1 motif 15 |
| 3.35E-12 | **-1.67** | **CAPN6** | Calpain 6 |
| 3.80E-20 | **-1.67** | **ADRA2C** | Adrenoceptor alpha 2C |
| 1.33E-07 | **-1.66** | **PLEKHG4B** | Pleckstrin homology and RhoGEF domain containing G4B |
| 1.04E-04 | **-1.65** | **DEPDC1B** | DEP domain containing 1B |
| 6.47E-07 | **-1.65** | **SEMA3D** | Semaphorin 3D |
| 2.79E-03 | **-1.64** | **SH3PXD2A** | SH3 and PX domains 2A |
| 4.98E-03 | **-1.64** | **COL1A1** | Collagen type I alpha 1 chain |
| 1.16E-02 | **-1.63** | **C1orf61 / MIR9-1** | *Chromosome 1 open reading frame 61* / MicroRNA 9-1 |
| 2.38E-05 | **-1.63** | **CENPF** | Centromere protein F |
| 5.25E-09 | **-1.63** | **FBXO15** | F-box protein 15 |
| 4.47E-10 | **-1.62** | **CDK1** | Cyclin dependent kinase 1 |
| 3.18E-03 | **-1.62** | **FOXM1** | Forkhead box M1 |
| 3.18E-03 | **-1.62** | **ZNF367** | Zinc finger protein 367 |
| 5.85E-03 | **-1.61** | **FAM81B** | Family with sequence similarity 81 member B |
| 1.75E-04 | **-1.61** | **UBE2T** | Ubiquitin conjugating enzyme E2 T |
| 1.55E-04 | **-1.60** | **CASC15** | Cancer susceptibility candidate 15 (non-protein coding) |
| 8.94E-03 | **-1.60** | **SST** | Somatostatin |
| 1.94E-31 | **-1.60** | **CDC42EP1** | CDC42 effector protein 1 |
| 1.85E-10 | **-1.60** | **TPX2** | TPX2, microtubule nucleation factor |
| 7.98E-09 | **-1.59** | **NANOS1** | Nanos C2HC-type zinc finger 1 |
| 4.27E-03 | **-1.59** | **CDCA3** | Cell division cycle associated 3 |
| 3.71E-08 | **-1.59** | **ABCB5** | ATP binding cassette subfamily B member 5 |
| 2.92E-05 | **-1.59** | **PHLDB2 / PLCXD2** | Pleckstrin homology like domain family B member 2 / Phosphatidylinositol specific phospholipase C X domain containing 2 |
| 5.36E-03 | **-1.58** | **CENPM** | Centromere protein M |
| 1.97E-06 | **-1.58** | **ASPM** | Abnormal spindle microtubule assembly |
| 1.99E-06 | **-1.58** | **ANLN** | Anillin actin binding protein |
| 5.04E-03 | **-1.57** | **RGS6** | Regulator of G-protein signaling 6 |

| | | | |
|---|---|---|---|
| 1.97E-04 | **-1.57** | **CDKN2C** | Cyclin dependent kinase inhibitor 2C |
| 1.09E-04 | **-1.57** | **FILIP1** | Filamin A interacting protein 1 |
| 4.42E-10 | **-1.57** | **ENTPD8** | Ectonucleoside triphosphate diphosphohydrolase 8 |
| 3.07E-04 | **-1.56** | **MLXIPL** | MLX interacting protein like |
| 4.35E-19 | **-1.56** | **UNC5B** | Unc-5 netrin receptor B |
| 8.15E-06 | **-1.56** | **MYBL2** | MYB proto-oncogene like 2 |
| 2.82E-06 | **-1.56** | **TOP2A** | Topoisomerase (DNA) II alpha |
| 2.66E-07 | **-1.55** | **MFAP4** | Microfibrillar associated protein 4 |
| 1.58E-12 | **-1.55** | **UNC13D** | Unc-13 homolog D |
| 3.79E-12 | **-1.55** | **CDC42EP2** | CDC42 effector protein 2 |
| 2.48E-06 | **-1.55** | **CA2** | Carbonic anhydrase 2 |
| 3.81E-02 | **-1.54** | **SPC25** | SPC25, NDC80 kinetochore complex component |
| 2.85E-17 | **-1.54** | **CCND2** | Cyclin D2 |
| 4.96E-12 | **-1.54** | **EDN2** | Endothelin 2 |
| 1.57E-05 | **-1.54** | **SERTAD4** | SERTA domain containing 4 |
| 1.97E-02 | **-1.54** | **AQP3** | Aquaporin 3 (Gill blood group) |
| 1.15E-17 | **-1.54** | **ST3GAL4** | ST3 beta-galactoside alpha-2, 3-sialyltransferase 4 |
| 8.78E-03 | **-1.53** | **SMPD3** | Sphingomyelin phosphodiesterase 3 |
| 1.23E-08 | **-1.53** | **DIAPH3** | Diaphanous related formin 3 |
| 7.64E-03 | **-1.53** | **USP43** | Ubiquitin specific peptidase 43 |
| 2.42E-06 | **-1.53** | **THBS2** | Thrombospondin 2 |
| 1.45E-13 | **-1.52** | **COL3A1 / MIR3606** | Collagen type III alpha 1 chain / MicroRNA 3606 |
| 3.23E-04 | **-1.52** | **TMEM235** | Transmembrane protein 235 |
| 5.51E-18 | **-1.52** | **ABCG1** | ATP binding cassette subfamily G member 1 |
| 4.18E-11 | **-1.52** | **PLXNB3 / SRPK3** | Plexin B3 / SRSF protein kinase 3 |
| 3.25E-03 | **-1.52** | **ZNF467** | Zinc finger protein 467 |
| 1.13E-03 | **-1.52** | **CDC20** | Cell division cycle 20 |
| 9.70E-30 | **-1.52** | **MFGE8** | Milk fat globule-EGF factor 8 protein |
| 1.01E-03 | **-1.51** | **C14orf80** | *Chromosome 14 open reading frame 80* |
| 8.23E-15 | **-1.51** | **CA11** | Carbonic anhydrase 11 |
| 6.61E-05 | **-1.51** | **GTSE1** | G2 and S-phase expressed 1 |
| 9.06E-06 | **-1.51** | **GRIK3** | Glutamate ionotropic receptor kainate type subunit 3 |
| 3.14E-06 | **-1.51** | **NTN1** | Netrin 1 |
| 1.46E-24 | **-1.51** | **PYGM** | Phosphorylase, glycogen, muscle |
| 2.35E-03 | **-1.51** | **PCSK2** | Proprotein convertase subtilisin/kexin type 2 |
| 4.14E-05 | **-1.50** | **KIFC1** | Kinesin family member C1 |
| 4.13E-04 | **-1.50** | **SLC25A35** | Solute carrier family 25 member 35 |
| 2.54E-02 | **-1.50** | **LINC01186** | *Long intergenic non-protein coding RNA 1186* |
| 7.91E-24 | **-1.50** | **CA9** | Carbonic anhydrase 9 |

Genes are classified according to the fold change with a *P*-value and fold-change cut-off of $P < 0.05$ and FC < -1.5, respectively. FC, fold-change.

**Supplementary Table S1**. Glucocorticoids hormones levels in iRPE cell culture media. basement membrane matrices. Green rectangles induicate cell culture conditions at th corticosteroids were added to iRPE cells. Red rectangle indicates presence of corticos

| | Cell culture medium | F (nM) | B (nM) |
|---|---|---|---|
| A | **RDMw/oA** (*n* = 5) | 0,00 | 12,88 |
| B | **ECFM** (*n* = 5) | 0,00 | 0,00 |

| | Cell culture media constituent | F (nM) | B (nM) |
|---|---|---|---|
| A | **DMEM GlutaMAX** (*n* = 1) | 0,00 | 0,00 |
| A | **Ham's F-12** (*n* = 1) | 0,00 | 0,00 |
| B | **DMEM no phenol red** (*n* = 5) | 0,00 | 0,00 |
| B | **Charcoal FBS** (*n* = 1) | 0,00 | 0,00 |

| | Basement membrane matrix | F (nM) | B (nM) |
|---|---|---|---|
| N/A | **Matrigel** (*n* = 1) | 0,00 | 0,00 |

F, cortisol; B, corticosterone; S, 11-deoxycortisol; DOC, Deoxycorticosterone; nM, nan differentiation medium without vitamin A; ECFM, experimental corticosteroid-free mediu glucose, GlutaMAX™ Supplement, HEPES medium; Ham's F-12, Ham's F-12 Nutrient DMEM, high glucose, HEPES, no phenol red; Charcoal FBS, Fetal Bovine Serum, cha B, ECFM-related.

, cell culture media constituents and
e time exogenous experimental
sterone in the RDMw/oA medium.

| S (nM) | DOC (nM) |
|---|---|
| 0,00 | 0,00 |
| 0,00 | 0,00 |

| S (nM) | DOC (nM) |
|---|---|
| 0,00 | 0,00 |
| 0,00 | 0,00 |
| 0,00 | 0,00 |
| 0,00 | 0,00 |

| S (nM) | DOC (nM) |
|---|---|
| 0,00 | 0,00 |

omolar; RDMw/oA, retinal
um;DMEM GlutaMAX, DMEM, high
Mix medium; DMEM no phenol red,
rcoal stripped; A, RDMw/oA-related;
